# The Genetics of Differential Gene Expression Related to Fruit Traits in Strawberry (*Fragaria ×ananassa*)

**DOI:** 10.3389/fgene.2019.01317

**Published:** 2020-02-07

**Authors:** Christopher Barbey, Max Hogshead, Anne E. Schwartz, Nadia Mourad, Sujeet Verma, Seonghee Lee, Vance M. Whitaker, Kevin M. Folta

**Affiliations:** ^1^Horticultural Sciences Department, IFAS, University of Florida, Gainesville, FL, United States; ^2^Gulf Coast Research and Education Center, IFAS, University of Florida, Wimauma, FL, United States

**Keywords:** eQTL analysis, pathway analysis, anthocyanins, pectin, transcriptomics, strawberry (*Fragaria ×ananassa* Duch.)

## Abstract

Octoploid strawberry (*Fragaria ×ananassa*) is a major specialty crop under intense annual selection for traits relating to plant vigor and fruit quality. Most functional validation experiments rely on transgenic or transient gene expression assays in the mature receptacle. These findings are not typically translatable to breeding without identifying a natural genetic source of transcript level variation, and developing reliable markers for selection in octoploids. Expression QTL (eQTL) analysis is a genetic/transcriptomic association approach for identifying sequence variants predicting differential expression. This eQTL study analyzed a wide array of mature receptacle-expressed genes, encompassing the majority of total mature receptacle transcript accumulation and almost all strawberry genes described in the literature. These results identified segregating genetic variants associated with the differential expression of hundreds of strawberry genes, many with known interest to breeders. Several of these eQTL pertain to published genes whose expression levels have been demonstrated to influence mature receptacle phenotypes. Many include key genes of the phenylpropanoid pathway, vitamin C, carotenoid, pectin, and receptacle carbohydrate/sugar metabolism. These subgenome-specific genetic markers may allow breeders to select for desired ranges of target gene expression. These results may also guide basic research efforts and facilitate the identification of causal genes underlying trait QTL.

## Introduction

Strawberry is a major specialty crop cultivated worldwide for its sweet and flavorful receptacle, which is referred to commonly as a fruit. Strawberry is under intense breeder selection for new cultivars based on diverse traits. These include receptacle color, firmness, sweetness, yield, flowering time, shipping quality, shelf life, nutrition, flavors, aromas, and disease resistance. The genomics era has provided a dense collection of phenotypically important genes that have been experimentally validated *via* transgenic analysis. However, this basic research often stops short of application, as genetic markers associated with traits are not coincidentally developed for use in breeding. Several resource and technology advances have recently converged to enable high-quality octoploid expression quantitative trait loci (eQTL) analysis. These include an octoploid genome reference (Edger et al., 2019), high-density subgenomic genotyping *via* the IStraw35 platform (Verma et al., 2017), and octoploid reference-based transcriptomics assembly.

eQTL analysis relates genotypic and transcriptomic data to identify segregating genomic regions influencing differential gene expression. Identifying eQTL provides major advantages over pure transcriptomic analysis. The results of an eQTL analysis identify the subset of genes whose differential expression is determined by genotype, the extent of that genetic influence, and markers that may be used for selection of desired gene expression ranges. These selectable markers are potentially useful for application where strawberry phenotypes are known to be influenced by transcript abundance. These include genes which have been characterized *via* transgenic overexpression or silencing in the strawberry receptacle. These eQTL markers may be applied to translate transgenic discoveries into breeding tools. In addition, eQTL controlling transcripts of undetermined function in strawberry can support candidate gene evaluation and trait-based QTL cloning. In one example, simple cross-referencing of trait QTL and eQTL markers identified a causal aroma biosynthesis gene in melon (Galpaz et al., 2018). In strawberry, eQTL experiments helped identify the γ-decalactone biosynthesis gene in the octoploid mature receptacle even while limited to incomplete *de novo* and diploid reference-based RNAseq assemblies (Sánchez-Sevilla et al., 2014). Using the recent subgenome-scale octoploid genome for ‘Camarosa’, 76 mature receptacle-expressed disease resistances genes (R-genes) were identified to be under the control of an eQTL (Barbey et al., 2019).

Most *cis*-eQTL are caused by sequence variants in or near the gene promoter region (Michaelson et al., 2009). As the approximate causal locus of *cis*-QTL is known, the resolution limits of the IStraw35 genotyping array can be measured. The distance from the originating gene locus to the most-correlated subgenomic marker, when sampled across hundreds of *cis*-eQTL, essentially creates a probability distribution for QTL size-resolution in studies under similar conditions. This distribution can be usefully applied to octoploid QTL studies where *a priori* knowledge of the causal variant locus is not known.

In this work, three octoploid strawberry populations were generated from cultivars varying for fruit quality attributes, such as firmness, sweetness, aroma, and flavor compounds (Vance et al., 2011; Whitaker et al., 2011). Mature receptacle transcriptomes from identical developmental stages were generated and compared against genotype. Analyzed transcripts include those with comparatively high accumulation, those representing differentially expressed genes, and a near-complete list of all published octoploid strawberry genes. Data from the octoploid ‘Camarosa’ strawberry gene expression atlas (Sánchez-Sevilla et al., 2017) were used to profile the expression of these genes throughout the plant. Genetic associations were filtered based on false-discovery rate (FDR) adjusted *p*-value, effect size, minor allele frequency, and other criteria. Collectively, these results specify the major genetics-based expression differences between cultivars, and the selectable genetics predicting them. These findings bridge basic and applied biology and provide a means to convert previous molecular research directly into plant breeding efforts.

## Materials and Methods

### Plant Materials

Three strawberry flavor and aroma populations were created from Florida cultivars and also ‘Mara des Bois’ which possesses unique receptacle quality and aroma traits (**Figure S1**). These populations were derived from the crosses ‘Florida Elyana’ × ‘Mara de Bois’ (population 10.113), ‘Mara des Bois’ × ‘Florida Radiance’ (population 13.75), and ‘Strawberry Festival’ × ‘Winter Dawn’ (population 13.76). Mature receptacles were harvested fully ripe from the field during winter growing seasons at the Gulf Coast Research and Education Center (GCREC) in Wimauma, Florida. Populations 13.75 and 13.76 were harvested during the winter of 2014. Population 10.133 was sampled on January 20, February 11, February 25, and March 18, 2011 (Chambers, 2013). Harvest days were selected based on dry weather and moderate temperature, both on the day of harvest and for several days preceding harvest.

### Genotyping of Octoploid Strawberry Lines

The Affymetrix IStraw35 Axiom^®^ SNP array (Verma et al., 2017) was used to genotype 61 individuals consisting of parents and progeny from crosses of ‘Mara de Bois’ × ‘Florida Elyana’, ‘Mara des Bois’ × ‘Florida Radiance’, and ‘Strawberry Festival’ × ‘Winter Dawn’ (**Figure S1**). Sequence variants belonging to the Poly High Resolution (PHR) and No Minor Homozygote (NMH) marker classes were included for association mapping. Mono High Resolution (MHR), Off-Target Variant (OTV), Call Rate Below Threshold (CRBT), and Other marker quality classes were discarded and not used for mapping. Individual marker calls inconsistent with disomic Mendelian inheritance from parental lines were removed. Genetic relatedness was evaluated using the VanRaden method using GAPIT v2 package (Tang et al., 2016) in R (**Figure S2**).

### Transcriptome Analysis

Octoploid mature receptacle transcriptomes from 61 individuals were sequenced *via* Illumina paired-end RNAseq (avg. 65 million 2× 100-bp reads), consisting of parents and progeny from crosses of ‘Florida Elyana’ × ‘Mara de Bois’, ‘Florida Radiance’ × ‘Mara des Bois,’ and ‘Strawberry Festival’ × ‘Winter Dawn.’ Reads were trimmed and mapped to the *Fragaria* ×*ananassa* octoploid ‘Camarosa’ annotated genome (Edger et al., 2019) using CLC Genomic Workbench 11 with a mismatch cost of 2, insertion cost of 3, deletion cost of 3, length fraction of 0.8, similarity fraction of 0.8, and 1 maximum hit per read. Reads which mapped equally well to multiple loci were discarded. RNAseq counts were calculated in Transcripts Per Million (TPM). Transcript levels were normalized *via* the Box-Cox transformation algorithm (Box and Cox, 1964) performed in R-Studio (Racine, 2011) prior to genetic correlation. The BLAST2GO pipeline (Conesa et al., 2005) was used to annotate the full ‘Camarosa’ predicted gene compliment. Raw reads from the strawberry gene expression atlas study (Sánchez-Sevilla et al., 2017) were aligned to the ‘Camarosa’ genome using identical procedures, with biological replicates averaged and compared for tissue-based expression using ClustVis (Metsalu and Vilo, 2015) with default parameters.

### Identification of High-Variance and Highly Expressed Genes

The 2,000 mature receptacle transcripts with the highest coefficient of variation between samples were identified *via* 1-Pearson correlation distance using the heat map clustering algorithm in CLC Genomics Workbench 11 (**Figure S3**). The 2,000 mature receptacle transcripts with the highest total expression were identified by calculating the sum total expression for each ‘Camarosa’ transcript across all samples (**Figure S4**).

### Retrieval of Published Strawberry Gene Sequences

All 607 non-redundant mRNA accessions under the query “*Fragaria ×ananassa*” were retrieved from the public databases collectively housed at NCBI. This list included all transiently modified strawberry genes compiled in a review (Carvalho et al., 2016) as well as other recently characterized genes in strawberry. Of these, 493 accessions contained an annotated coding sequence (CDS). All retrieved sequences not containing a CDS annotation were determined to be misannotated microsatellite sequences and discarded. These extracted coding sequences were compared by BLAST to identify the most identical gene in the octoploid ‘Camarosa’ reference genome, identifying 380 unique putative orthologs. This figure was somewhat smaller than the query size, as many deposited mRNA sequences represent alleles or splicoforms corresponding to a single orthologous gene in the non-haplotype specific cv. ‘Camarosa’ genome. Transcriptome data for these corresponding ‘Camarosa’ genes were used in eQTL analysis.

### Genetic Association of Gene Expression (eQTL)

Genome-wide association was performed *via* a mixed linear model approach using the GAPIT v2 package (Tang et al., 2016) in R. The diploid *Fragaria vesca* physical map was used to orient marker positions, as current genetic maps in octoploid do not include a majority of the available IStraw35 markers. eQTL were evaluated for significance based on the presence of multiple co-locating markers of *p*-value < 0.05 after false discovery rate (FDR) correction for multiple comparisons (Benjamini and Hochberg, 1995). *Cis* vs *trans* eQTL determinations were made by corroborating the known ‘Camarosa’ gene position with the eQTL marker position in the physical map.

## Results

**Figure 1:**
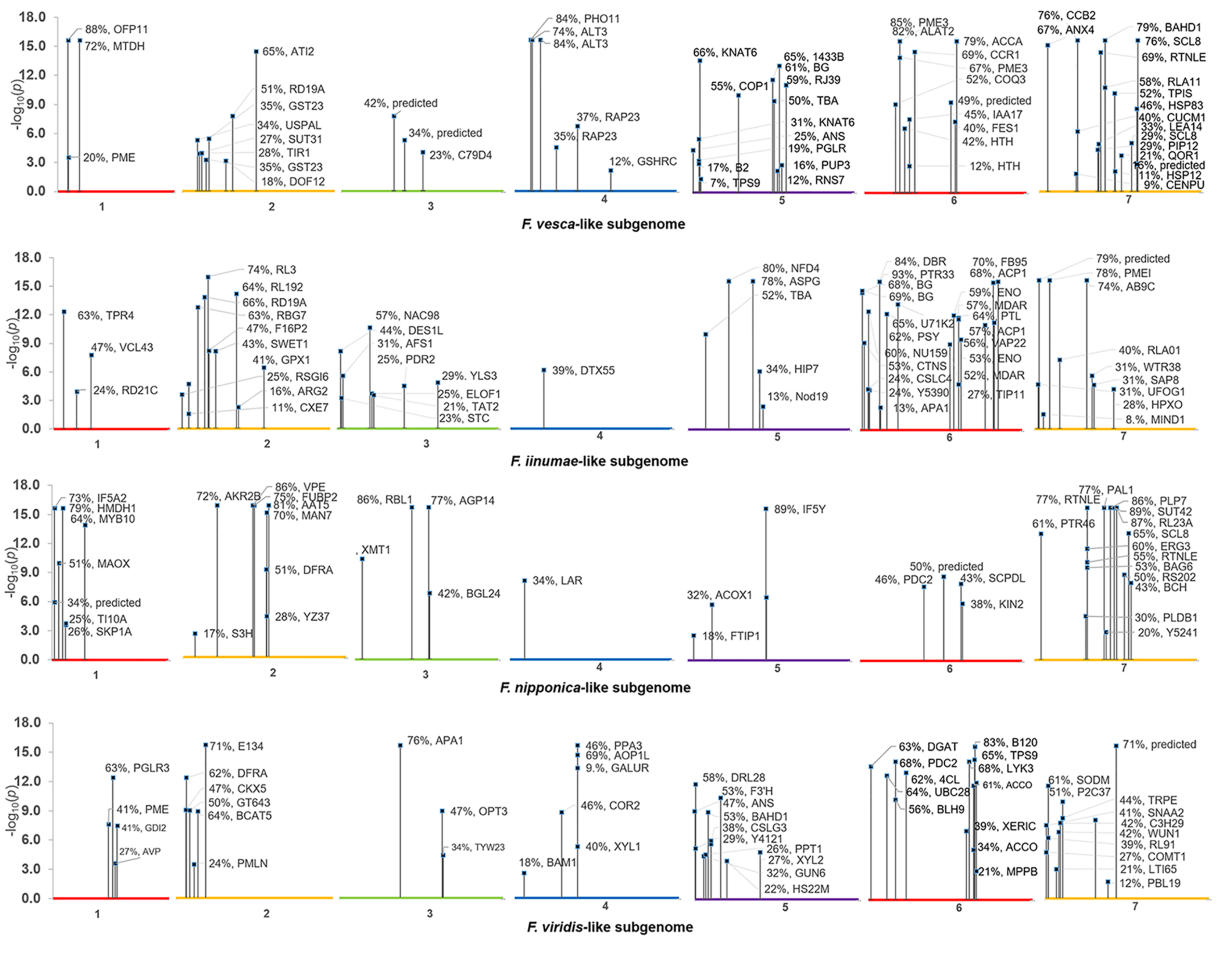
Composite Manhattan plot for octoploid fruit *cis*-eQTL. The ‘Camarosa’ genome position of the most-correlated marker for each *cis*-eQTL is shown with single-marker *p*-value, effect size and BLAST2GO gene annotation.

**Figure 2:**
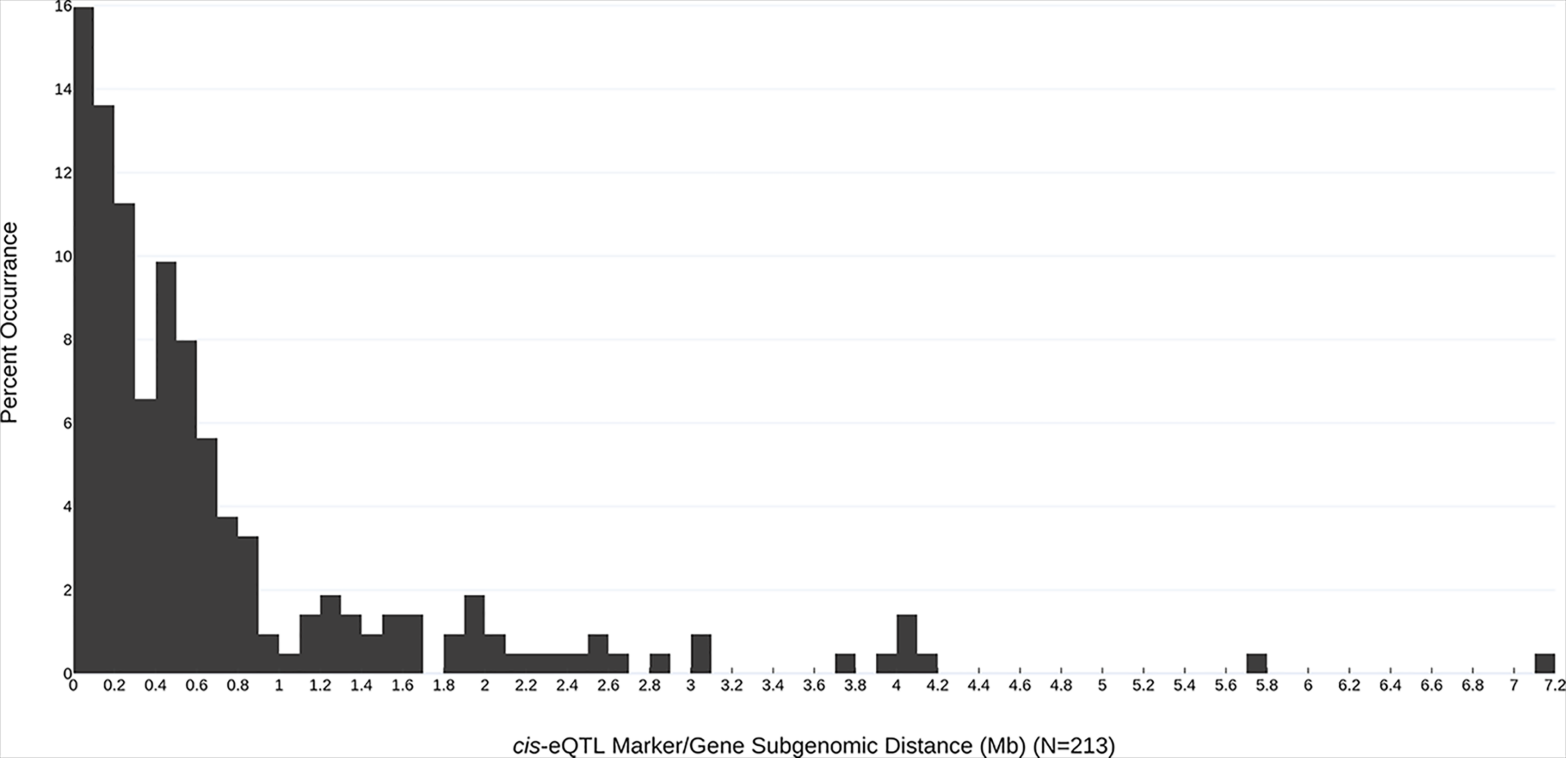
Subgenomic distances (Mb) between the most-correlated *cis*-eQTL marker and the originating gene locus. The frequency of each marker/gene distance observation is indicated (bin size = 0.1 Mb).

In all, 268 robust *cis* and *trans* eQTL were discovered relating to the mature receptacle expression of 224 octoploid strawberry genes (**Table S1**). *cis*-eQTL were found abundantly across all subgenomes (**Figure 1**). A vast majority of the identified eQTL loci were found proximal to the originating gene locus, within 0.42 Mb median distance on the same homoeologous chromosome (0.053% of the ‘Camarosa’ octoploid genome length) (**Table S1**). A frequency plot of *cis*-eQTL (N = 213) marker/gene distances is presented in **Figure 2**. A plurality (16%) of *cis*-eQTL gene/marker distances are located within 0.1 Mb of the originating gene locus. Larger gene/marker distances are progressively rarer until reaching a frequency minimum around 1 Mb. Approximately 90% of gene/marker distances are within this interval. Most eQTL display stepwise changes in transcript accumulation corresponding to allelic dosage, with many displaying near-zero transcript expression in one homozygous state (**Table S1**, **File S1**).

Thirty-five eQTL relate to strawberry alleles known to influence fruit traits *via* transgenic analyses (**Table 1**) or which were experimentally described in strawberry literature (**Table 2**). The magnitude of experimental overexpression/silencing for each gene, and its biological effect in a given cultivar, is shown in comparison with eQTL-associated transcript ranges. In several cases these eQTL naturally replicate transcript accumulation levels observed after transgenic manipulation (**Tables 1** and **2**). These include the published strawberry mature receptacle transcription factors *FanMYB10* (Medina-Puche et al., 2014; Kadomura-Ishikawa et al., 2015; Medina-Puche et al., 2015) *FanEOBII* (Medina-Puche et al., 2015), *FanSnRK2.6* (Han et al., 2015), *FanSLC8* (Pillet et al., 2015), and the phenylpropanoid-modulating genes *FanCCR* (Yeh et al., 2014)*, FanF′3H* (Miyawaki et al., 2012)*, FanGT1* (Griesser et al., 2008), and *FanFra a3* (Muñoz et al., 2010). Boxplots showing mature receptacle transcript ranges stratified by marker genotype (AA, AB, or BB) are provided for all genes, together with ANOVA omnibus *p* values and *post hoc* significances (**File S1**).

**Table 1:** eQTL pertaining to transgenically characterized *F ×ananassa* genes (cv. Camarosa exact putative ortholog).

Gene	Upregulation	Cultivar	Effect caused by overexpression	Cv. Camarosa Gene	eQTL TPM range	Marker (*cis*)
*FanCCR* (Cinnamoyl-CoA Reductase)	no increased expression	Elsanta and Calypso*	altered content of phenolic acid derivatives	maker-Fvb6-1-augustus-gene-164.26	500-2800	AX-166507442
*FanEOBII* (Emission of Benzoid II)	2500% to 6500%	Elsanta	increased eugenol content; increased transcript levels of FanEGS2	maker-Fvb6-2-snap-gene-289.57	0-30	AX-166525602
*FanFra a3* (Fruit Allergen a 3)	not shown	Elsanta	no effect	augustus_masked-Fvb4-3-processed-gene-199.4	0-8	AX-166513822
*FanMYB10* (Myeloblastosis 10)	1.7-fold	Sachinoka	increased anthocyanin content; altered expression of anthocyanin biosynthesis genes	maker-Fvb1-3-augustus-gene-144.30	3-18	AX-123434353
*FanSnRK2.6* (Sucrose Nonfermenting1-Related Protein Kinase 2.6)	4-fold	Benihoppe	ripening delay and temperature insensitivity; increased firmness; decreased anthocyanin content; altered transcription of aroma and cell-wall genes.	maker-Fvb2-2-augustus-gene-185.33	0.25-4.5	AX-166521262
**Gene**	**Silencing (%)**	**Cultivar**	**Effect caused by silencing**	**Cv. Camarosa eQTL Gene**	**eQTL TPM range**	**Marker (*cis*)**
*FanCCR* (Cinnamoyl-CoA Reductase)	83.3	Elsanta and Calypso*	no effect	maker-Fvb6-1-augustus-gene-164.26	500-2800	AX-166507442
*FanEOBII* (Emission of Benzoid II)	88.3–99.5	Elsanta	decreased eugenol content; down-regulation of two eugenol-related genes, FanCAD1 and FanEGS2.	maker-Fvb6-2-snap-gene-289.57	0-31	AX-166525602
*FanF’3H* (Flavonoid 3′-Hydroxylase)	approx. 70	Sachinoka	mild reduction in fruit color	augustus_masked-Fvb5-2-processed-gene-78.0	1-7.5	AX-89893608
*FanFra a3* (Fruit Allergen a 3)	60	Elsanta	altered phenylpropanoid pathway precursor and anthocyanin levels; altered transcript levels of FanPAL and FanCHS genes	augustus_masked-Fvb4-3-processed-gene-199.4	0-8	AX-166513822
*FanGT1* (Glycosyltransferase 1)	85	Elsanta	mild reduction in color; reduced anthocyanin content; increased flavan-3-ol content	maker-Fvb7-3-augustus-gene-14.53	5-52	AX-166517042
*FanMYB10* (Myeloblastosis 10)	90	Elsanta	decreased anthocyanin and eugenol content; decreased transcript accumulation of ripening-related TFs and FanEOBII	maker-Fvb1-3-augustus-gene-144.30	3-18	AX-123434353
	80	Sachinoka	decreased anthocyanin levels; altered transcript levels of flavonoid biosynthesis pathway-related genes			
*FanSCL8* (Scarecrow-Like Protein 8)	67 to 93	Strawberry Festival	altered transcript accumulation of flavonoid biosynthesis-related genes	augustus_masked-Fvb7-2-processed-gene-277.8	1-45	AX-166508726
*FanSnRK2.6* (Sucrose Nonfermenting1-Related Protein Kinase 2.6)	approx. 90	Benihoppe	acceleration of fruit ripening; increased anthocyanin content; decreased firmness; altered transcript accumulation of pigment, aroma, and cell-wall metabolism genes	maker-Fvb2-2-augustus-gene-185.33	0.25-4.5	AX-166521262

*Transgenic background: FanCHS silenced. Portions of this table are derived from a review by Carvalho et al. (2016).

**Table 2:** eQTL pertaining to a published *F ×ananassa* gene (Camarosa’ paralog or homoeolog).

Gene	GenBank no.	Cv. Camarosa eQTL Gene	eQTL TPM range	Marker
*FanGALUR* (D-galacturonate reductase)	AF039182	maker-Fvb4-1-augustus-gene-196.31	500-2600	AX-166505923
*FanACP1* (Acyl carrier chloroplastic)	AF041386	maker-Fvb6-3-augustus-gene-389.35 (cis)	1-47	AX-123614270
		(trans)	10-2600	AX-166525307
*FanPDC2* (Pyruvate decarboxylase 2)	AF193791	maker-Fvb6-2-augustus-gene-209.38	500-3000	AX-166508206
*FanCOBRA* (glycosylphosphatidylinositol-anchored protein)	AY642687	maker-Fvb5-2-snap-gene-76.47 (cis)	5-45	AX-166524220
		(trans)	5-45	AX-123361263
*FanGT643* (Glycosyltransferase family 64)	AY679583	snap_masked-Fvb2-3-processed-gene-49.22	0.75-2.5	AX-166507157
*FanPLDB1* (Phospholipase D beta 1)	AY679584	maker-Fvb7-1-augustus-gene-162.30 (cis)	2-9	AX-166526588
		(trans)	2-9	AX-123365640
*FanQOR1* (Quinone oxidoreductase 1)	AY679595	maker-Fvb7-2-augustus-gene-257.57	2-25	AX-166516995
*FanYJNA* (Uncharacterized AAA domain-containing)	AY679604	maker-Fvb7-4-augustus-gene-17.32	0.25-4	AX-166518372
*FanXERIC* (RING-type E3 ubiquitin transferase)	AY679613	augustus_masked-Fvb6-4-processed-gene-297.11 (cis)	0.25-7.5	AX-166526717
		(trans)	0.5-7.5	AX-89787062
*FanAKR* (Aldo/keto reductase)	AY703448	maker-Fvb4-1-augustus-gene-141.33	10000-25000	AX-123367100
*FanACCO* (ACC oxidase)	AY706156	maker-Fvb6-4-augustus-gene-306.55	70-1400	AX-166516039
*FanLEA45* (Late embryogenesis abundant 4-5)	DQ011163	maker-Fvb6-1-augustus-gene-201.55 (cis)	125-1200	AX-166524532
		(trans)	120-900	AX-166518183
*FanPPA3* (Purple acid phosphatase 3)	DQ074726	maker-Fvb4-1-snap-gene-183.52 (cis)	2-90	AX-166505923
		(trans)	2-90	AX-166505413
*FanF16P2* (D-fructose-1,6-bisphosphate 1-phosphohydrolase)	EU185335	maker-Fvb2-4-snap-gene-100.35	2.5-15	AX-166503535
*FanPS*Y (Phytoene chloroplastic)	FJ784889	maker-Fvb6-3-augustus-gene-80.43 (cis)	10-230	AX-166515961
		(trans)	10-250	AX-89914629
*FanZD*S (Zeta-carotene desaturase)	FJ795343	maker-Fvb6-2-augustus-gene-256.63 (cis)	5-55	AX-123357007
		(trans)	5-41	AX-166516136
*FanNBS1* (TIR-NBS-LRR type protein)	HQ845018	maker-Fvb1-2-augustus-gene-63.28	5-62	AX-89789432
*FanNCED1* (9-cis-epoxycarotenoid dioxygenase 1)	JN006161	augustus_masked-Fvb3-3-proces sed-gene-13.7	100-200	AX-166518894
FanRD21C (Probable cysteine protease)	JN979371	maker-Fvb1-2-augustus-gene-106.27 (cis)	100-260	AX-166517617
		(trans)	0-175	AX-89853113
*FanMDAR* (Monodehydroascorbate reductase)	JQ320104	maker-Fvb6-3-augustus-gene-273.47 (cis)	50-250	AX-123356923
		(trans)	50-150	AX-166513999
*FanGR* (Glutathione reductase)	JQ339738	maker-Fvb4-3-augustus-gene-315.32	0-0.50	AX-166508304
*FanFT1* (FT-like protein)	JQ364958	maker-Fvb6-2-augustus-gene-271.50 (cis)	0-0.50	AX-123363588
		(trans)	200-3900	AX-123364123
*FanANS* (Anthocyanidin synthase)	JQ923457	maker-Fvb5-1-augustus-gene-7.57	0.50-9	AX-89831030
*FanLAR* (Leucoanthocyanidin reductase)	JX134096	maker-Fvb4-2-augustus-gene-44.51	25-120	AX-166526717
*FanTIR1* (Transport inhibitor response 1)	JX292971	maker-Fvb2-2-augustus-gene-51.44	50-200	AX-89910815
*FanG6PDH* (Glucose-6-phosphate dehydrogenase cytoplasmic)	KC433888	maker-Fvb6-4-augustus-gene-13.60	18-40	AX-166511756
*FanGPX* (Glutathione peroxidase 2)	KC433890	maker-Fvb2-4-snap-gene-265.134	18-40	AX-166511756
*FanMnSOD* (Mn-superoxide dismutase)	KC433893	snap_masked-Fvb7-4-processed-gene-40.42	150-400	AX-123363180
*FanDFR* (Dihydroflavonol 4-reductase)	KC894054	maker-Fvb2-1-augustus-gene-255.45	50-250	AX-166511816
*FanBCH1* (β-carotene hydroxylase 1)	KC967656	maker-Fvb7-1-augustus-gene-290.59	0-1.3	AX-166508808
*FanLFY1* (LEAFY-like protein 1)	KF006322	maker-Fvb3-4-augustus-gene-275.43	0-1.8	AX-166513103
*FanCERK1* (chitin elicitor receptor kinase 1-like protein)	KT224458	snap_masked-Fvb6-4-processed-gene-308.25 (cis)	1-14	AX-123366408
		(trans)	1-14	AX-123365571
*FanMRLK4*7 (FERONIA-like receptor kinase)	KX374343	augustus_masked-Fvb6-4-processed-gene-67.11	4-29	AX-166508268
*FanCOP1* (Constitutive photomorphogenesis 1)	KX583676	maker-Fvb5-1-snap-gene-145.25	1-3.75	AX-123367149
*FanPHO11* (Phosphate transporter PHO1 homolog 1)	KY190225	maker-Fvb4-3-snap-gene-46.51	0-1.75	AX-123363868
*FanGT2D* (trihelix transcription factor)	KY368685	maker-Fvb6-3-augustus-gene-283.340	0-2.2	AX-89868974
*FanPIP12* (Plasma membrane intrinsic protein subtype 1 aquaporin)	KY453775	maker-Fvb7-2-augustus-gene-182.44	0-65	AX-123359604
*FanJAZ1* (jasmonate ZIM-domain protein)	MF511104	maker-Fvb1-3-augustus-gene-43.42	0-5.5	AX-123365102
*FanRJ39* (uncharacterized protein)		maker-Fvb5-1-snap-gene-288.58	125-900	AX-123358407

**Figure 3:**
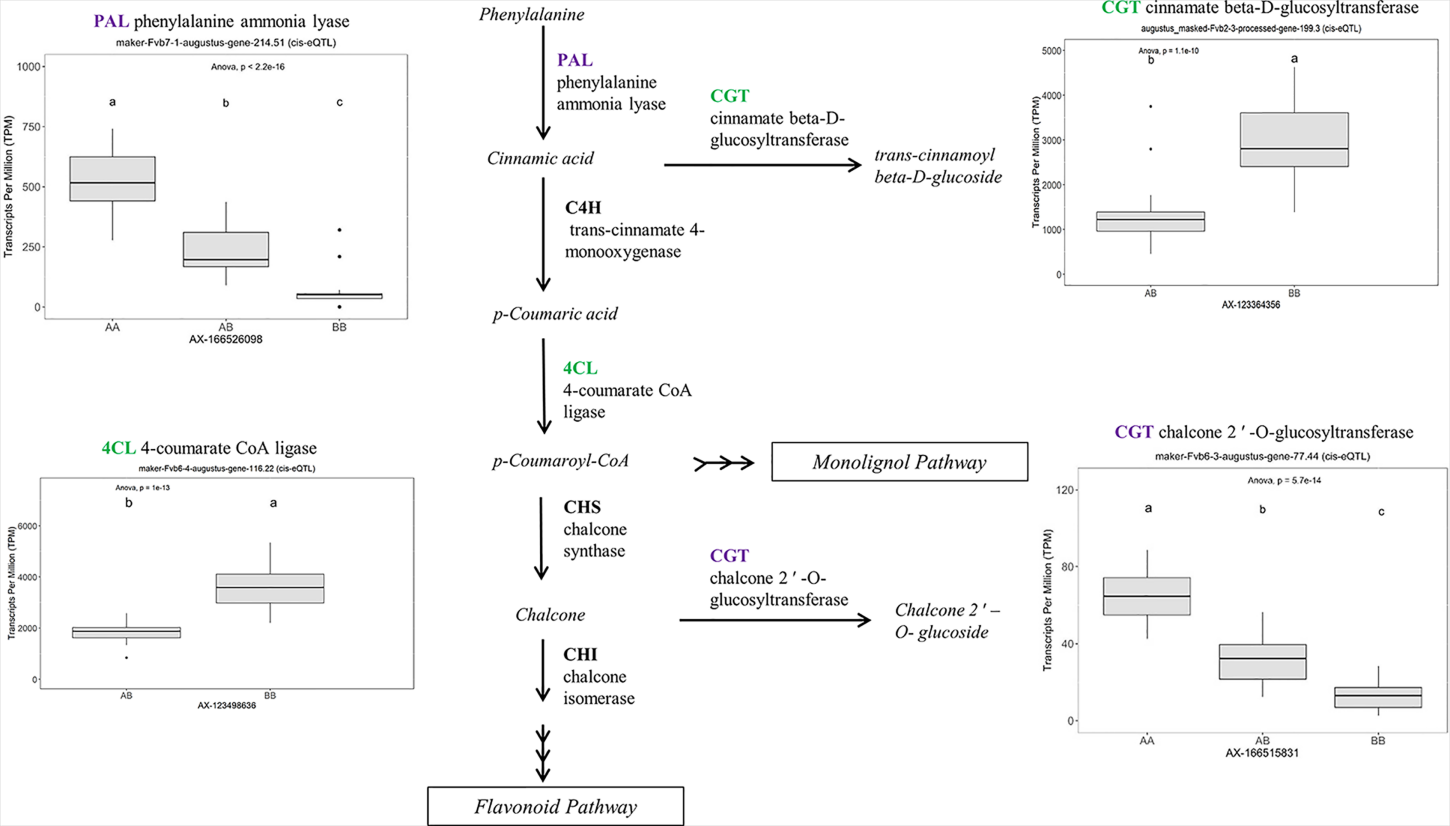
eQTL controlling transcript accumulation of key genes in the strawberry phenylpropanoid pathway (PPP). Marker effect sizes are indicated by boxplots stratified by allelic state (AA, AB, or BB) and shown with ANOVA *p*-values. eQTL genes based on the ‘Camarosa’ genome are indicated as either possessing the highest sequence identity to the published sequence (purple) or not (green). Letters represent statistically separable means *via* Tukey’s HSD post hoc test (p < 0.05).

**Figure 4:**
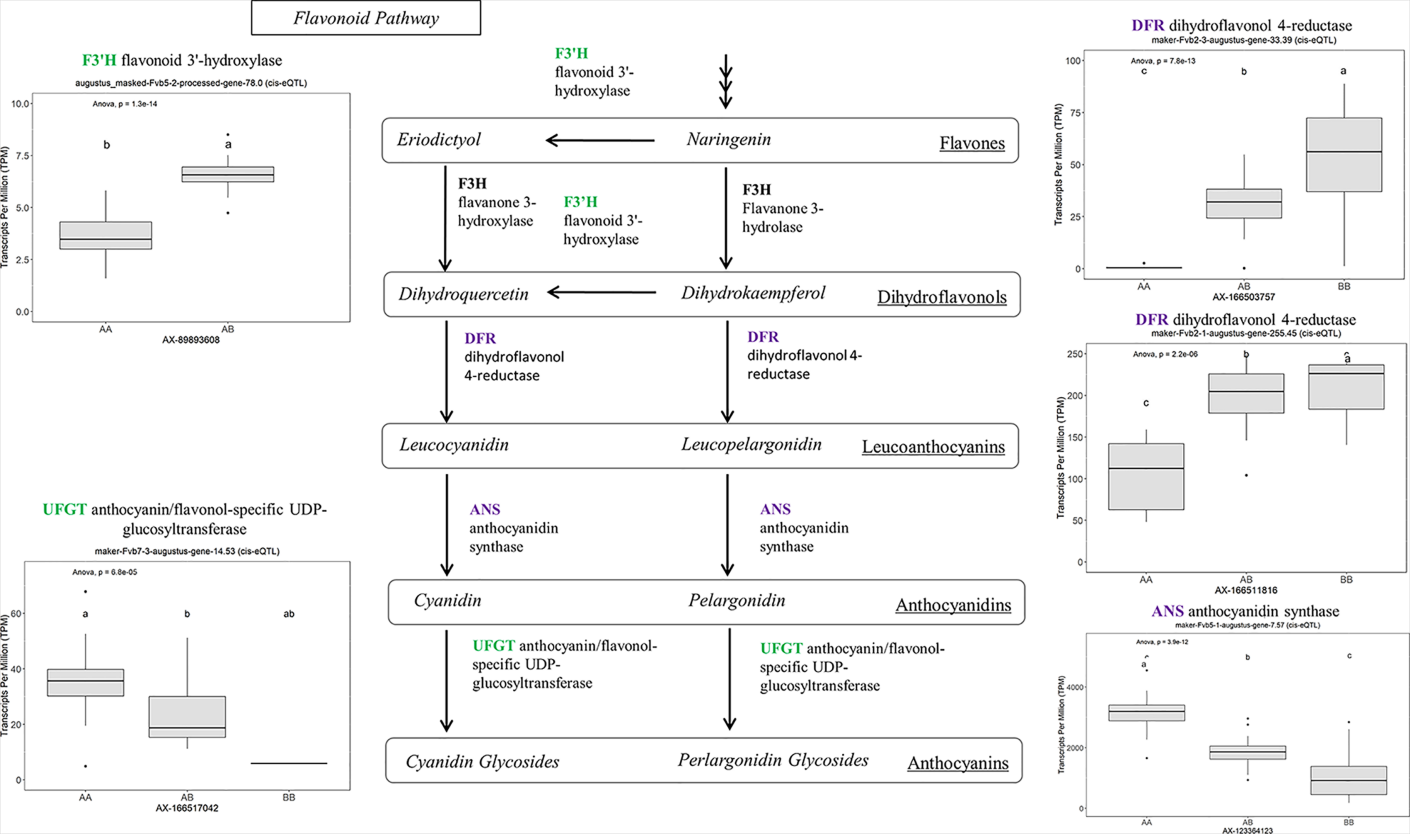
eQTL controlling transcript accumulation of key genes in the flavonoid pathway. Marker effect sizes are indicated by boxplots stratified by allelic state (AA, AB, or BB) and shown with ANOVA *p*-values. eQTL genes based on the ‘Camarosa’ genome are indicated as either possessing the highest sequence identity to the published sequence (purple) or not (green). Letters represent statistically separable means *via* Tukey’s HSD post hoc test (p < 0.05).

**Figure 5:**
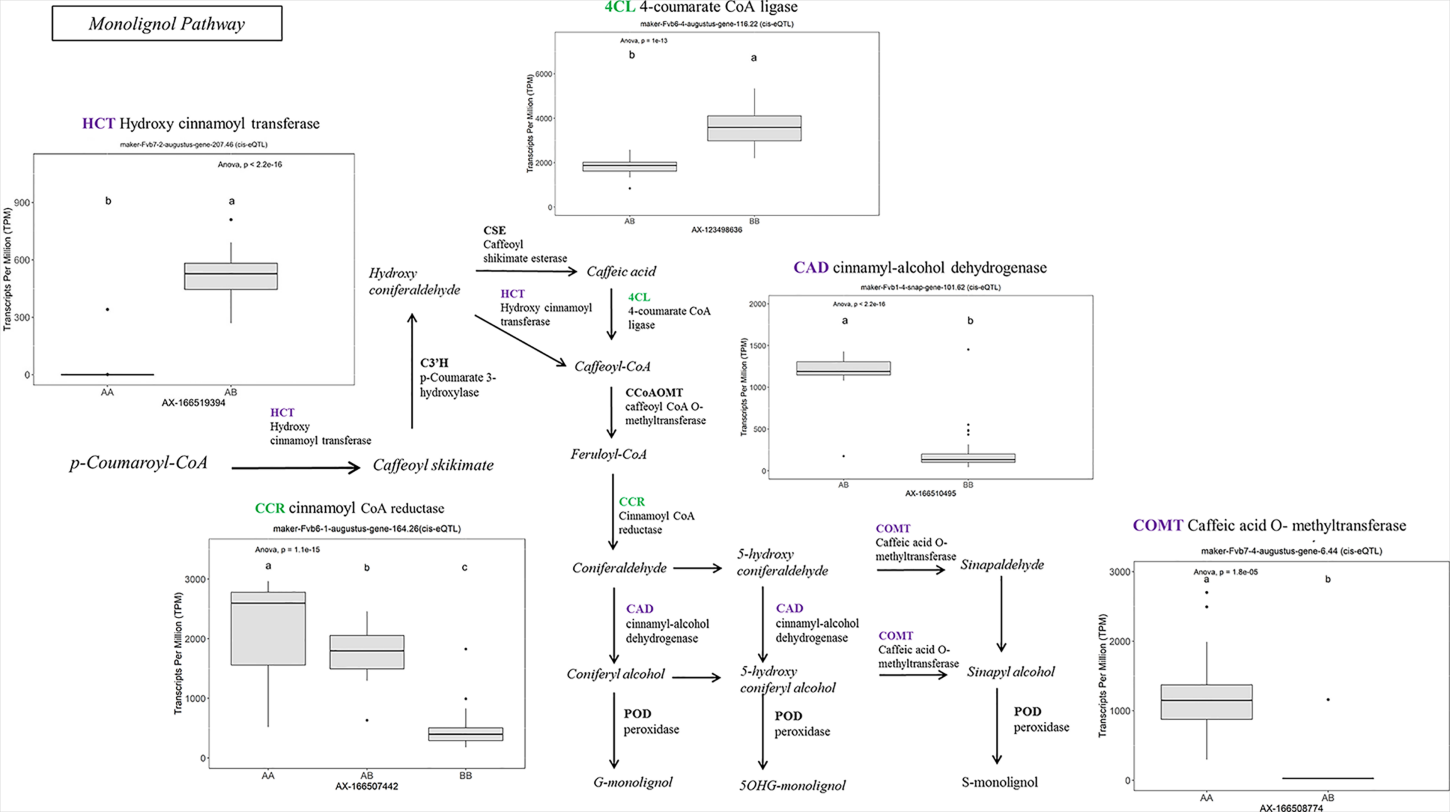
eQTL controlling monolignol pathway gene expression. Marker effect sizes are indicated by box plots stratified by allelic state (AA, AB, or BB) and shown with ANOVA *p*-values. eQTL genes based on the ‘Camarosa’ genome are indicated as either possessing the highest sequence identity to the published sequence (purple) or not (green). Letters represent statistically separable means *via* Tukey’s HSD post hoc test (p < 0.05).

**Figure 6:**
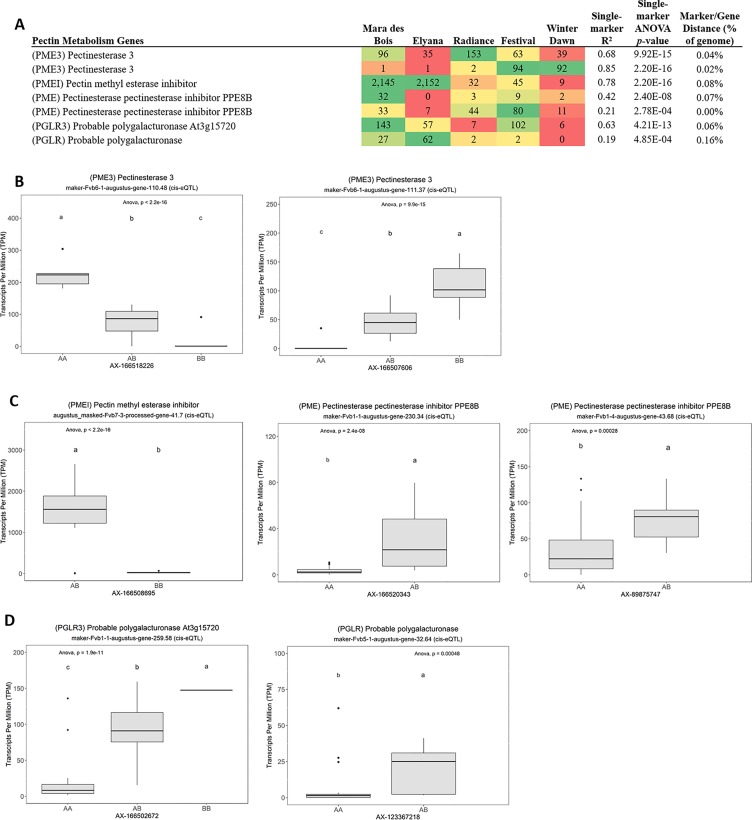
eQTL pertaining to strawberry pectin metabolism. **(A)** Transcript accumulation in parental lines, GWAS-derived FDR *p* values and narrow-sense heritability estimates, single-marker R^2^ and *p* values are shown. Phenotype distributions based on allelic state are shown for **(B)** pectin esterases, **(C)** pectin esterase inhibitors, and **(D)** polygalacturonases. Letters represent statistically separable means *via* Tukey’s HSD post hoc test (p < 0.05).

Many of the remaining eQTL-associated genes were further investigated due to their collective participation in mature receptacle quality pathways. These include key genes relevant to phenylpropanoid metabolism (**Figure 3**), flavonoid biosynthesis (**Figure 4**), monolignol biosynthesis (**Figure 5**), and pectin metabolism (**Figure 6**). For fruit phenylpropanoid metabolism, eQTL were discovered for *PHENYLALANINE AMMONIA LYASE, 4-COUMARATE CoA LIGASE, CINNAMATE β -D-GLUCOSYLTRANSFERASE, and CHALCONE 2′-O-GLUCOSYLTRANSFERASE* (**Figure 2**). Relating to fruit flavonoid biosynthesis, eQTL were discovered for *FLAVONOID 3′-HYDROXYLASE, ANTHOCYANIN SYNTHASE, ANTHOCYANIN/FLAVONOL-SPECIFIC UDP-GLUCOSYLTRANSFERASE*, and *DIHYDROFLAVONOL 4-REDUCTASE* (two homoeologs) (**Figure 4**). For fruit monolignol biosynthesis, eQTL were discovered for *HYDROXYCINNAMOYL TRANSFERASE, CINNAMOYL CoA REDUCTASE, 4-COUMARATE CoA LIGASE, ALCOHOL DEHYDROGENASE*, and *CAFFEIC ACID O-METHYLTRANSFERASE* (**Figure 5**). These eQTL-associated genes are outlined in context with their pathways, and boxplots demonstrate transcript distribution ranges distributed by marker genotype and with supporting statistics. For fruit pectin metabolism, eQTL were discovered for *PECTIN ESTERASE 3* (two non-homoelogs), *PECTIN METHYLESTERASE INHIBITOR* (two homoeologs and one non-homoeolog), and *POLYGALACTURANASE* (two non-homoeologs) (**Figure 6**). Pectin metabolism-related mature receptacle transcript expression values are shown across parental cultivars (**Figure 6A**) with boxplots of TPM stratified by marker genotype (**Figure 6B**). Genome-wide Manhattan plots for these genes are provided in **File S3**, demonstrating multiple significant markers at each locus.

Other eQTL are highlighted for genes whose transcript levels are known in strawberry to influence sugar/carbohydrate metabolism, **L**-ascorbic acid content, and carotenoid metabolism (**Table 3**). Large mature receptacle transcript abundance differences are dependent upon allele dosage for genes *D-GALACTURONATE REDUCTASE, PHYTOENE CHLOROPLASTIC*, bidirectional sugar transporter SWEET1 and many others. A complete list all 268 eQTL and supporting statistics are presented in **Table S1**, including mean +/- SD transcript values for each marker genotype, minor allele, and minor allele frequencies, transcript variance explained *via* single-marker analysis (omnibus R^2^), narrow-sense heritability estimates for mature receptacle transcript accumulation (h^2^) with FDR adjusted *p-*values, phase (*cis* or *trans*), physical distance between the originating gene and the *cis-*eQTL, and citations for published genes. Raw transcript abundances in mature receptacles across the eQTL populations (**Table S2**) and in various ‘Camarosa’ tissues (Sánchez-Sevilla et al., 2017) (**Table S3**) are provided. Complete IStraw35 genotypes for all individuals is provided in **File S2**.

**Table 3:** eQTL pertaining to fruit quality pathway genes.

Fruit Quality Genes	TPM (AA genotype)	TPM (AB genotype)	TPM (BB genotype)	Minor Allele	Minor Allele Frequency	*p*-value (FDR adjusted)	Estimated h^2^	Single marker R^2^	Single-marker *p*-value
**L-Ascorbic Acid Metabolism**									
(GALUR) D-galacturonate reductase	597.5 ± 98.1	1218.4 ± 602.6	1969.4 ± 323.4	A	0.29	6.9E-04	0.71	0.10	2.2E-03
(MDAR) Monodehydroascorbate reductase	135.6 ± 37.4	78.9 ± 33.9	18.4 ± 40.2	B	0.24	2.8E-04	0.68	0.53	8.9E-10
**Carotenoid Metabolism**									
(PSY) Phytoene chloroplastic	36.9 ± 39.0	129.3 ± 36.2	222.9 ± NA	B	0.33	1.8E-03	0.60	0.63	5.5E-13
(ZDS) Zeta-carotene chloroplastic chromoplastic	28.4 ± 12.4	16.4 ± 5.9	7.7 ± 4.2	A	0.42	3.5E-03	0.91	0.50	2.0E-09
(BCH) Beta-carotene 3- chloroplastic	–	.6 ± .4	.1 ± .1	A	0.30	4.02E-02	0.92	0.43	1.16E-08
**Monolignol Pathway**									
(BAHD1) BAHD acyltransferase At5g47980	7.8 ± 50.3	587.5 ± 250.8	–	B	0.48	2.3E-05	1	0.79	2.2E-16
(CCR1) Cinnamoyl- reductase 1	2864.5 ± 1483.0	1755.4 ± 425.8	455.3 ± 294.3	A	0.23	2.5E-02	0.51	0.69	2.6E-15
(MTDH) Probable mannitol dehydrogenase	–	1150 ± 325.9	197.2 ± 217.3	B	0.26	8.4E-04	0.37	0.72	2.2E-16
**Sugar/Carbohydrate Metabolism**									
(ENO) Enolase	–	124.8 ± 72.3	6.6 ± 21.6	A	0.29	3.8E-04	0.73	0.59	7.7E-13
(E134) Endo-1,3 1,4-beta-D-glucanase	20.1 ± 12.6	75.4 ± 27.3	167.3 ± 13.8	B	0.36	4.1E-04	0.95	0.71	3.7E-16
(PGLR3) Probable polygalacturonase At3g15720	18.5 ± 30.3	90.5 ± 33.3	182.6 ± 49.9	B	0.31	5.2E-04	0.18	0.63	4.2E-13
(MAN7) Mannan endo-1,4-beta-mannosidase 7	582.7 ± 170.1	322.6 ± 95.2	103.6 ± 100.3	A	0.46	5.8E-04	0.49	0.70	1.2E-15
(AGP14) Arabinogalactan peptide 14	–	479.6 ± 139.0	78.5 ± 78.8	A	0.43	8.0E-04	0.99	0.78	2.2E-16
(SWET1) Bidirectional sugar transporter SWEET1	9.0 ± 35.0	91.4 ± 61.2	–	B	0.19	8.1E-04	0.82	0.43	1.2E-08
(XYL1) Alpha-xylosidase 1	45.5 ± 39.5	4.5 ± 6.8	.5 ± NA	A	0.31	8.3E-04	0.82	0.40	5.1E-06
(XYL2) Beta-xylosidase alpha-L-arabinofuranosidase 2	.2 ± .6	52.6 ± 49.5	–	B	0.33	2.4E-03	0.53	0.28	1.6E-05
(MTDH) Probable mannitol dehydrogenase	–	1150 ± 325.9	197.2 ± 217.3	A	0.20	8.4E-04	0.37	0.72	2.2E-16
(BGL24) Beta-glucosidase 24	52.5 ± 23.2	21.9 ± 24.0	1.9 ± 1.5	A	0.45	1.3E-03	0.76	0.42	1.5E-07
(GUN6) Endoglucanase 6	230.7 ± 88.6	711.9 ± 436.2	651.1 ± 237.6	B	0.40	2.2E-03	1	0.32	4.1E-05
(GBA2) Non-lysosomal glucosylceramidase	–	42.6 ± 26.3	11.2 ± 5.5	A	0.17	4.3E-03	0.29	0.48	9.4E-10
(XYL1) Alpha-xylosidase 1	45.5 ± 39.5	4.5 ± 6.8	.5 ± NA	A	0.31	8.3E-04	0.82	0.40	5.1E-06
(XYL2) Beta-xylosidase alpha-L-arabinofuranosidase 2	.2 ± .6	52.6 ± 49.5	–	B	0.33	2.4E-03	0.53	0.28	1.6E-05
(GPAT3) Probable glycerol-3-phosphate acyltransferase 3	13.5 ± 14.4	54.5 ± 34.0	–	B	0.28	5.4E-03	0.86	0.38	3.0E-07
(BAM1) Beta-amylase chloroplastic	89 ± 39.7	72.9 ± 25.8	29.7 ± 7.4	B	0.19	3.4E-02	0.77	0.19	2.8E-03
(STC) Sugar carrier protein C	73.1 ± 66.9	55.8 ± 43.5	13.5 ± 12.6	A	0.47	4.0E-02	0.75	0.23	6.5E-04
(CSLG3) Cellulose synthase G3	.6 ± 14.6	44.5 ± 20.6	76.7 ± 23.4	A	0.32	4.9E-02	0.82	0.38	9.9E-07
(CSLC4) Cellulose synthase C4	–	165 ± 51.5	100.4 ± 48.8	A	0.25	6.6E-03	0.58	0.25	5.1E-05
trans	–	165.0 ± 51.5	100.4 ± 48.8	A	0.25	6.6E-03		0.25	5.1E-05
(DGAT) Diacylglycerol O-acyltransferase	.5 ± .6	559.5 ± 252.9	–	B	0.35	1.3E-05	1	0.64	2.3E-14
(GLYK) D-glycerate 3-kinase, chloroplastic	97.5 ± 40.1	53.0 ± 11.2	9.8 ± 1.7	B	0.31	9.6E-03	0.45	0.54	1.9E-10
	84.3 ± 39.5	24.1 ± 20.6	–	B	0.12	9.6E-03		0.34	1.0E-06
(PDC2) Pyruvate decarboxylase 2	71.1 ± 33.3	1019.2 ± 527.0	2012.6 ± 510.1	A	0.48	6.9E-04	0.97	0.69	7.2E-15

**Figure 7:**
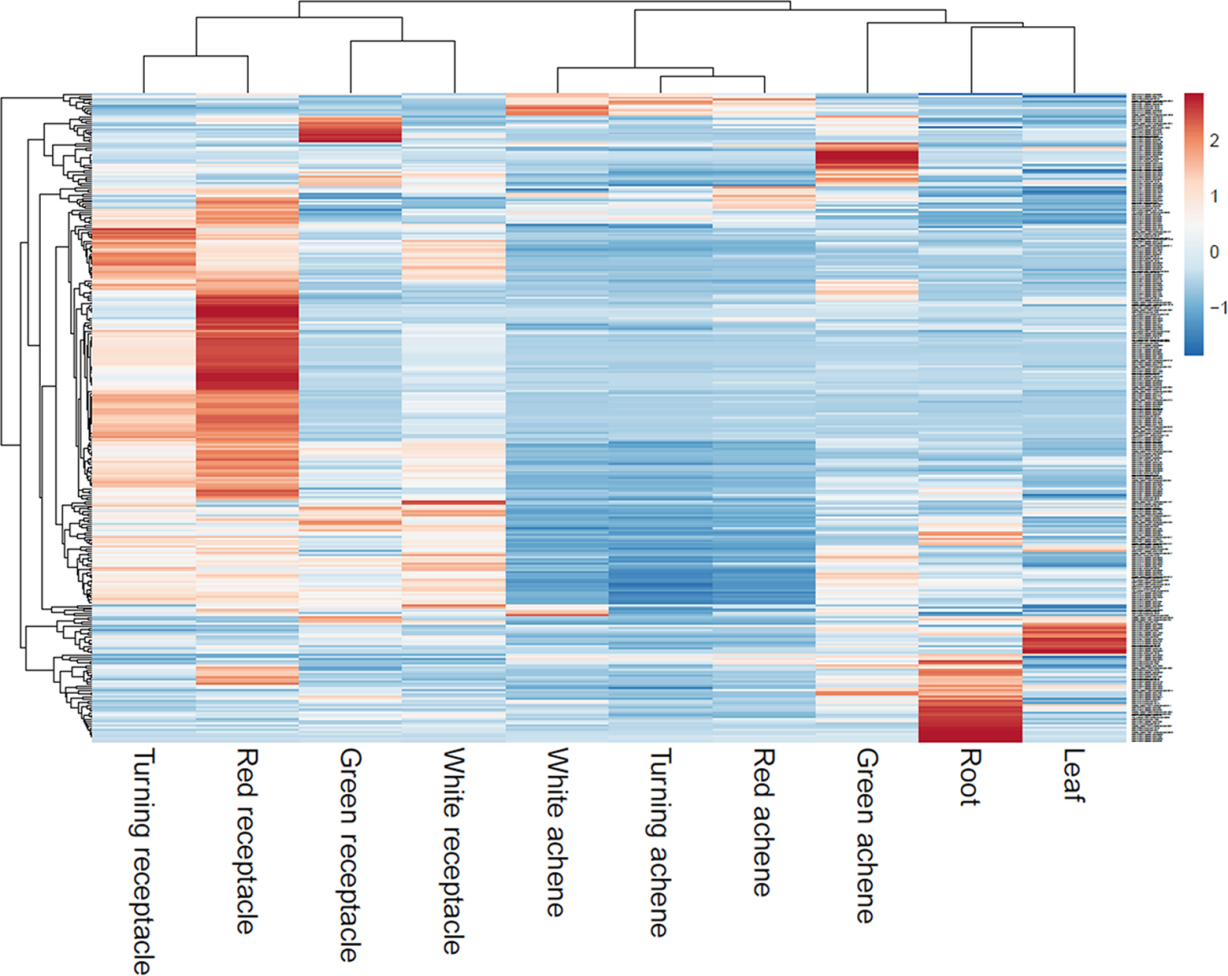
Transcript accumulation of fruit eQTL genes across various tissues. Scaled heatmap of gene expression reveals fruit eQTL genes are predominantly or exclusively expressed in the later fruit stages.

Reassembly of raw RNAseq data from various tissues of octoploid ‘Camarosa’ (Sánchez-Sevilla et al., 2017) determined that a majority of eQTL-associated transcripts predominate in the mature receptacle, and are upregulated with ripening (**Figure 7**). As an external test of the predictive power of each eQTL, the unused ‘Camarosa’ IStraw35 genotype and six mature receptacle transcriptome replicates (Sánchez-Sevilla et al., 2017) were tested against the eQTL-population transcript distributions for each marker genotype (AA, AB, or BB). Mean ‘Camarosa’ TPM fell within the 95% prediction interval for its marker genotype in 240 of 268 cases (**Table S1**).

## Discussion

This work identified eQTL genetic markers associated with differential mature receptacle transcript accumulation between strawberry genotypes. Most of the identified eQTL are *cis*-variants proximal to the originating gene locus in the ‘Camarosa’ genome and show stepwise increases in transcript accumulation according to allelic dosage, with many having near-zero TPM in one homozygous state. Loci demonstrating this behavior could correspond to gene presence/absence variants (PAVs) between the cultivars used as parents in this study. Gene PAVs is a major driver of agronomic trait variation in *brassica napus*, with nearly 40% of genes showing PAVs in the pangenome (Hurgobin et al., 2018). Gene PAVs are caused mainly by homeologous exchange (Hurgobin et al., 2018), which has extensively shaped the octoploid strawberry genome (Edger et al., 2019). It seems possible that PAVs could also be a major driver of diversity in octoploid strawberry. Discovery of octoploid eQTL, including those representing PAVs, is limited by the single reference genome available for octoploid transcriptome assembly. This eQTL study hints at the influence of gene PAVs in octoploid cultivar diversity. These eQTL can be immediately leveraged for basic biological investigation, and in some cases genetic selection for several strawberry traits.

### Pectin Metabolism

Pectin metabolism is a central feature of ripening-associated sweetening and softening. Pectin metabolism is mainly regulated by differential expression of pectin methylesterases and methylesterase inhibitors (Di Matteo et al., 2005). Several eQTL were identified for these genes, including three pectin methylesterase inhibitors (PMEI), two homoeologs of PECTIN METHYLESTERASE 3 (*PME3*), and two non-homologous POLYGALACTURONASE (*PG*) genes. The eQTL effect size is very strong for the PMEI gene “augustus_masked-Fvb7-3-processed-gene-41.7” (baseline average 29 TPM), where single segregating allele leads to expression increases in excess of 1,500 TPM (>50-fold increase) (R^2^ = 0.78, single-marker *p*-value 2.2e-16). This large difference is present among modern cultivars. High PMEI varieties include ‘Mara des Bois’ and ‘Florida Elyana’, while low-expression varieties include ‘Florida Radiance’, ‘Winter Dawn’, and ‘Strawberry Festival’. Transgenic analysis can be used to isolate a possible phenotypic effect of this highly variable gene, and determine an ideal allelic state for variety improvement.

### Phenylpropanoid Pathway

The phenylpropanoid pathway (PPP) influences many attributes of the strawberry receptacle, and many commercial strawberry breeding priorities are related to phenylpropanoid metabolism. These attributes include firmness and texture, flavor, color, ripening, quantitative disease resistance, shelf-life, and other facets of fruit quality (Dixon et al., 1996; Vogt, 2010; Peled-Zehavi et al., 2015). Several strawberry PPP genes have been characterized using transient expression analysis in the receptacle (Carvalho et al., 2016). Previous RNAseq-based network analyses in strawberry mature receptacles identified that PPP transcripts tend to be highly abundant and broadly variable between cultivars (Pillet et al., 2015). It is expected that PPP-associated transcript levels should vary in populations arising from crosses of strawberry cultivars with contrasting fruit qualities. This eQTL analysis advances previous findings in the strawberry PPP by identifying the specific octoploid subgenomic alleles which are variably expressed due to genetics, and the selectable sequence variants which predict them.

One eQTL in this category is the major transcription factor *FanMYB10* (EU15516, maker-Fvb1-3-augustus-gene-144.30) (R^2^ = 0.64, single-marker *p*-value 1.3e-14), which regulates flavonoid and phenylpropanoid metabolism (Medina-Puche et al., 2014). This gene has been studied in strawberry using transgenesis (Kadomura-Ishikawa et al., 2015), but natural variation has not previously been identified. This analysis identifies that the cultivars ‘Florida Radiance,’ ‘Strawberry Festival,’ and ‘Winterdawn’ have 5-to-8-fold greater *FanMYB10* transcript levels compared to ‘Mara des Bois’ and ‘Florida Elyana,’ and that these differences are heritable (h^2^ = 0.93). Relatively modest transient silencing (80-90% of normal) substantially decreased anthocyanin content, whereas relatively modest overexpression (170% of normal) increased anthocyanin content (Kadomura-Ishikawa et al., 2015). The eQTL for *FanMYB10* expression naturally approximates the expression level changes achieved through transgenesis. Genetic selection for this eQTL, and others related to the PPP, could lead to more efficient breeding methods for modified anthocyanin content and other PPP-related metabolites.

A robust eQTL was found for a putative *HYDROXYCINNAMOYL TRANSFERASE* (*FanHCT*, maker-Fvb7-2-augustus-gene-207.46) (R^2^ = 0.79, FDR-adjusted *p*-value 0.000023). Hydroxycinnamoyl transferases function in the PPP to generate diverse substrates for Cinnamoyl CoA reductase (CCR) proteins. The candidate *FanHCT* is among the most abundantly accumulating acyltransferases transcripts in the mature receptacle (averaging about 1:200 total transcripts). However, expression of this major transcript is exclusive to ‘Camarosa’, ‘Florida Radiance’, and segregating progeny (h^2^ = 1.0). Heterologous downregulation of *HCT* expression led to enrichment of H-lignins and improved cell wall saccharification in alfalfa (Jackson et al., 2008), a key process in strawberry receptacle ripening. Several other eQTL were found for other genes in the PPP, including *UDP-GLUCOSE : CINNAMATE GLUCOSYLTRANSFERASE*, an enzyme upstream of HCT.

### Vitamin and Nutrient-Associated Transcripts

Both *cis* and *trans* eQTL were identified for *D-GALACTURONIC ACID REDUCTASE* (*FanGalUR*, AF039182; FDR-adjusted p-value 0.0007). The expression of the *FanGalUR* transcript is heritable (h^2^ = 0.71) and shows substantial expression variation (500-2,000 TPM range determined by genotype). Previous research in strawberry demonstrated that L-ascorbic acid content is limited by *FanGalUR* transcript abundance (Agius et al., 2003). This limitation has also been confirmed in *F. chiloensis, F. virginiana*, and *F. moschata*, and experimentally validated in species outside of the *Fragaria* genus. Transgenic overexpression of the strawberry *FaGalUR* increased L-ascorbic acid content in *Arabidopsis thaliana* (Agius et al., 2003), *Lactuca sativa L*. (Lim et al., 2008), *Solanum lycopersicum* (Lim et al., 2016), and *Solanum tuberosum* (Hemavathi et al., 2009). It is therefore likely that selecting for increased *FanGalUR* transcript in strawberry will lead to increased L-ascorbic acid content.

L-ascorbic acid levels are influenced by additional factors including metabolite degradation (Cruz-Rus et al., 2011). The gene *MONODEHYDROASCORBATE REDUCTASE* (*MDAR*) is involved in oxidative stress tolerance and is described as a key component of fruit L-ascorbic acid repair (Cruz-Rus et al., 2011). Both *cis* and *trans-*eQTL were discovered for a published strawberry *FanMDAR* allele (JQ320104) (h^2^ = 0.68, FDR-adjusted *p-*value 0.00028), of which the *cis*-eQTL accounts for 53% of a 3-fold transcript variation (single-marker *p*-value 8.87e-10). It is possible this heritable fold-change difference contributes to L-ascorbic acid maintenance. This hypothesis could be quickly tested *post hoc* by examining this new genetic variant in strawberry lines with previously existing L-ascorbic acid data. Additional eQTL were found for the vitamin C antioxidant-associated genes *MN-SUPEROXIDE DISMUTASE* (*FanSODM*), *GLUTATHIONE PEROXIDASE* (*FanGPX*), and *GLUTATHIONE REDUCTASE* (*FanGR*) (Erkan et al., 2008) (**Table S2**).

### Carotenoids

Strawberry carotenoids provide fruit color and photoprotection, and are essential human nutrients (Ruiz-Sola and Rodríguez-Concepción, 2012). *Cis*-eQTL were discovered for published alleles of strawberry *PHYTOENE SYNTHASE* (*FanPSY*, FJ784889) (h^2^ = 0.60, FDR-adjusted *p*-value 0.0018) and *Ζ-CAROTENE DESATURASE* (*FanZDS*, FJ795343) (h^2^ = 0.91, FDR-adjusted *p*-value 0.0034). Single-marker analysis accounts for 62% and 50% of the observed mature receptacle differential expression between genotypes, respectively. *PSY* is a common control point for substrate flux into the carotenoid pathway in several plants (Fraser et al., 2007) and is often correlated with the upregulation of *ZDS* transcripts (Fanciullino et al., 2008). In strawberry fruit, Zhu et al., (2015) noted that *FanPSY* and *FanZDS* transcript accumulation varies between cultivars, and were modestly correlated with carotenoid levels. To assess the impact of *FanPSY* and *FanZDS*-related carotenoid content, these eQTL markers may be used to screen for seedlings that will abundantly express these genes in the fruit. A *cis*-eQTL accounting for 43% of a β-carotene hydroxylase (*FanBCH*) transcript accumulation variance was also discovered, however total accumulation was low (h^2^ = 0.92, single-marker *p*-value 1.2e-08).

### Fruit Ripening Transcription Factors

The strawberry receptacle ripening process is mainly determined by genetic factors (Perkins‐Veazie, 2010). Several eQTL were found for fruit-based transcription factors associated with the ripening process (**Figure 6**), including the negative regulator *FanSnRK2.6*, whose natural expression decreases with fruit ripening (Han et al., 2015). The phenotypic effects of *FanSnRK2.6* expression have been studied *via* transgenesis. Transgenic overexpression of *FanSnRK2.6* (400% of normal) in octoploid receptacles arrested ripening, while silencing (10% of normal) accelerated ripening (Han et al., 2015). The *cis*-QTL for *FanSnRK2.6* is associated with a ~5-fold difference in mature receptacle transcript accumulation between cultivars. As this is a similar range to that demonstrated by transgenesis, it is possible that eQTL marker selection could produce similar phenotypes to that observed by transgenesis. However, the influence of *FanSnRK2.6* is likely greatest in the developing receptacle, and it is unknown if this eQTL is predictive at earlier receptacle stages.

An eQTL was found for the transcription factor *EMISSION OF BENZENOID II* (*FanEOBII, KM099230*). This transcription factor has been experimentally characterized in *Fragaria ×ananassa* using transient overexpression (Medina-Puche et al., 2015). Transient overexpression of *FanEOBII* in the receptacle increased levels of eugenol, a desirable volatile organic compound (Medina-Puche et al., 2015). Ripening-related transcript accumulation of *FanEOBII* is elicited by *FanMYB10*, a phenylpropanoid pathway transcription factor whose eQTL was previously discussed.

An eQTL was also discovered for the flavonoid-associated transcription factor *SCARECROW-LIKE 8* (*FanSCL8, F. vesca- gene13212*). *FaSCL8* was identified as a flavonoid pathway regulator using transcriptome network correlation analysis, and experimentally shown to regulate accumulation of several flavonoid-associated transcripts (Pillet et al., 2015). Additional eQTL were found for one orthologous and one paralogous copy of *FanSCL8*. These gene copies have different expression patterns (Table S1), suggesting non-redundant functions.

### Bridging to Application in Strawberry Breeding

eQTL analysis is a tool for evaluating gene expression using genetics. These concrete genetic differences can be used to explore gene function, and serve as a bridge to marker/trait association. The biochemistry and genetics underlying many important traits in strawberry has been detailed in the literature, though few of these discoveries have been translated into practical markers for breeding. It is frequently the case that informative molecular research does not describe a source of beneficial genetics which can be selected by breeders. This eQTL analysis identified natural genetic variants influencing transcript variation, analogous to transgenic expression levels. These markers may also be used for targeted basic research aimed at genes/genetics/transcripts which are highly variable between cultivars. As these eQTL markers are derived from the widely used IStraw35 SNP array platform, eQTL markers can be easily cross-referenced with trait QTL experiments *in silico*. This approach can be used to help identify the causal basis of trait QTL in cases where differential expression contributes to traits. This approach can also be used *post-hoc* to rapidly test existing trait QTL.

These eQTL results highlight the shortcomings of transcriptomics-only driven candidate gene discovery. With RNAseq data alone, it is typically indiscernible whether differential expression is due to genetics, environment or stochastic effects. This genetic association study establishes that most *Fragaria ×ananassa* fruit transcripts are probably not influenced by differential genetics under normal growth conditions. Even among the biased set of 2,000 fruit genes with the highest transcriptional variance, only 8% were rigorously associated with segregating genetics (**Table S1**). As myriad genetic and environmental interactions can influence transcript accumulation, further work is warranted to widen the scope of this foundational analysis. Though multiple populations from different seasons were used in this analysis, these results pertain only to mature strawberry receptacle in normal field conditions, and the modest number of transcriptomes (61) is a limitation for estimating heritability and R^2^. Future RNAseq experiments performed in octoploid strawberry are encouraged to utilize low-cost IStraw-based genotyping to facilitate expression-QTL analysis.

## Author’s Note

eQTL analysis in octoploid strawberry uncovered genetic variants determining the differential expression of key fruit genes, including published genes where transcript-level variation is known to govern important traits.

## Data Availability Statement

The datasets generated for this study can be found in the Raw short read RNAseq data from fruit transcriptomes are available from the NCBI Short Read Archive under project SRP039356 (http://www.ncbi.nlm.nih.gov/sra/?term=SRP039356). Raw short read RNAseq data from the ‘Camarosa’ gene expression atlas (Sánchez-Sevilla et al., 2017) are available at the European Nucleotide Archive (https://www.ebi.ac.uk/ena) with the study reference PRJEB12420.

## Author Contributions

CB conceived and led the research experiment. MH, NM, and CB performed gDNA isolation and genotyping data filtering. AS, MH, NM, and CB evaluated eQTL candidates. AS, MH, and CB performed results collection, organization, and single-marker analysis. SV provided guidance in genotyping and QTL mapping. KF, SL, and VW contributed to project oversight and manuscript editing. CB and KF composed the manuscript. All authors read and approved the final manuscript.

## Funding

This work was supported by grants from the Florida Department of Agriculture and Consumer Services and the UF/IFAS Plant Breeding Graduate Initiative (VW and KF).

## Conflict of Interest

The authors declare that the research was conducted in the absence of any commercial or financial relationships that could be construed as a potential conflict of interest.
