# Supplementary material for: The Genetics of Differential Gene Expression Related to Fruit Traits in Strawberry (Fragaria ×ananassa)
Source: Front Genet. 2020 Feb 7;10:1317. doi: 10.3389/fgene.2019.01317 (PMC7025477; doi:10.3389/fgene.2019.01317)
Supplement: Supplementary file 1 [file DataSheet_1.pdf]

File S1. Boxplots for mature fruit transcript ranged stratified by marker genotype (AA, AB, or BB) for all genes together with ANOVA omnibus p values and post hoc significances.

(PHO11) Phosphate transporter
maker-Fvb4-3-snap-gene-46.51(cis-eQTL)

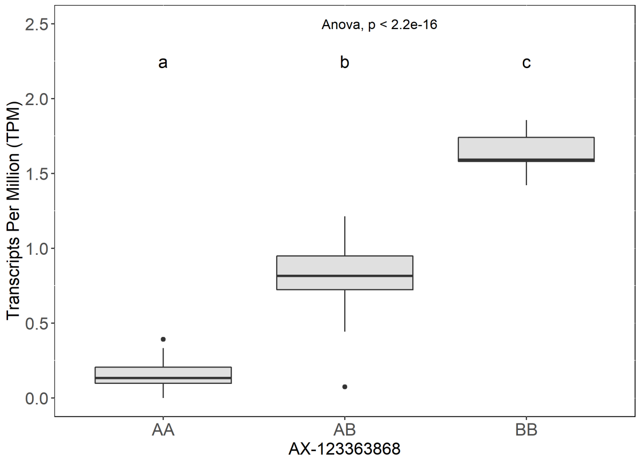

(SCL8) Scarecrow 8
augustus_masked-Fvb7-2-processed-gene-277.8 (cis-eQTL)

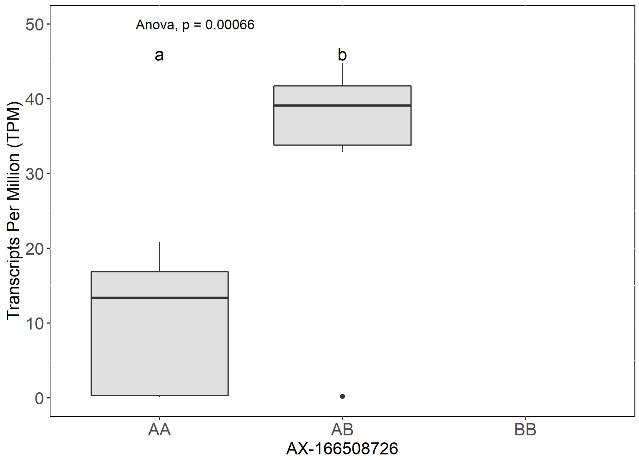

(CCR1) Cinnamoyl- reductase 1
maker-Fvb6-1-augustus-gene-164.26(cis-eQTL)

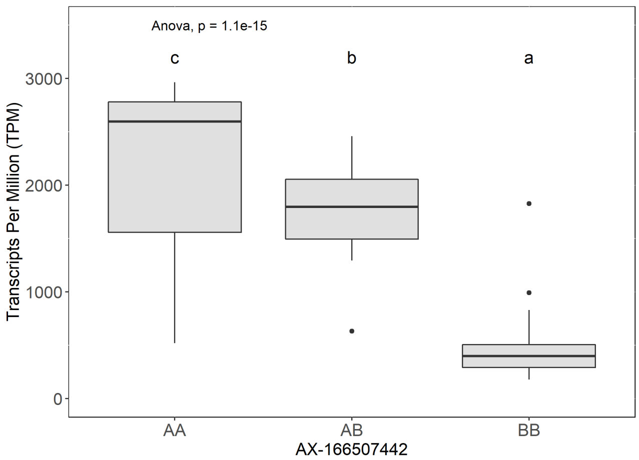

(PSY) Phytoene chloroplastic
maker-Fvb6-3-augustus-gene-80.43 (cis-eQTL)

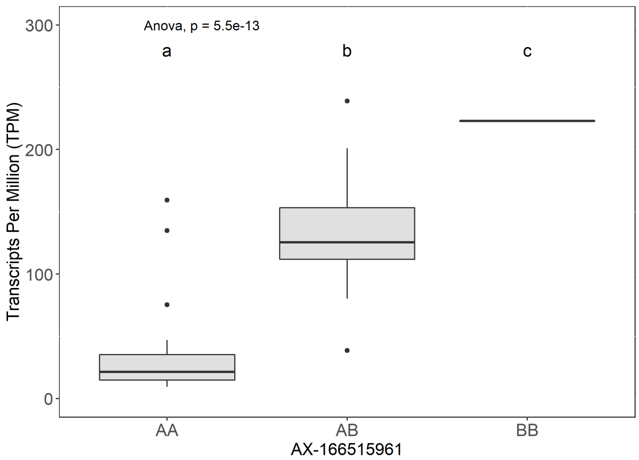

(SODM) Superoxide dismutase
snap_masked-Fvb7-4-processed-gene-40.42 (cis-eQTL)

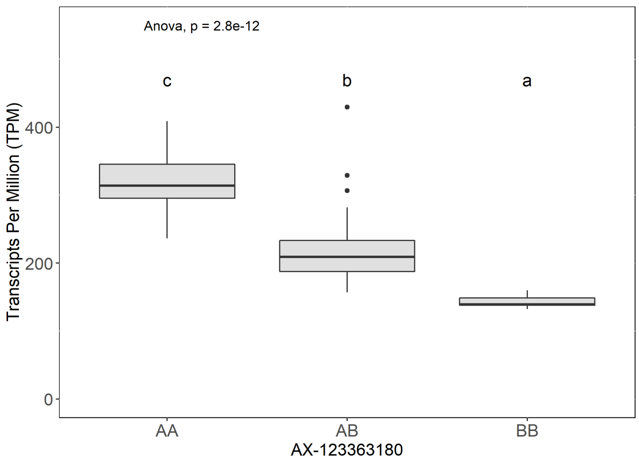

(RJ39) Uncharacterized protein RJ39
maker-Fvb5-1-snap-gene-288.58(cis-eQTL)

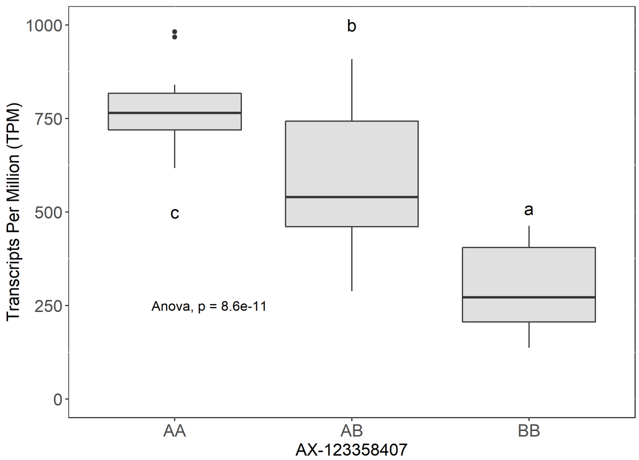

(MYB10) Transcription factor MYB10

maker-Fvb1-3-augustus-gene-144.30 (cis-eQTL)

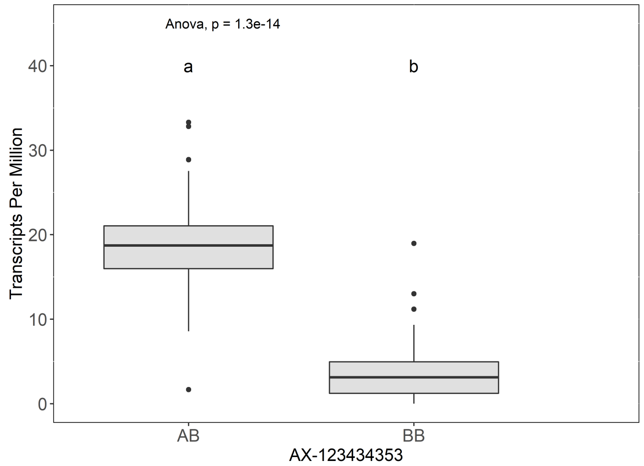

(PTL) Trihelix transcription factor PETAL LOSS

maker-Fvb6-3-augustus-gene-283.34 (cis-eQTL)

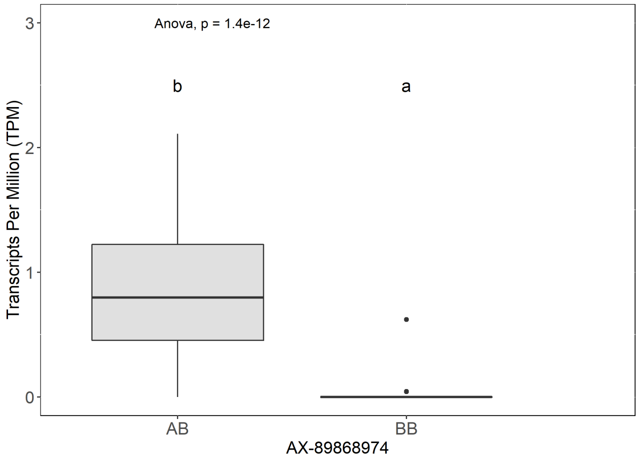

(COP1) E3 ubiquitin- ligase,Constitutive photomorphogenesis 1

maker-Fvb5-1-snap-gene-145.25(cis-eQTL)

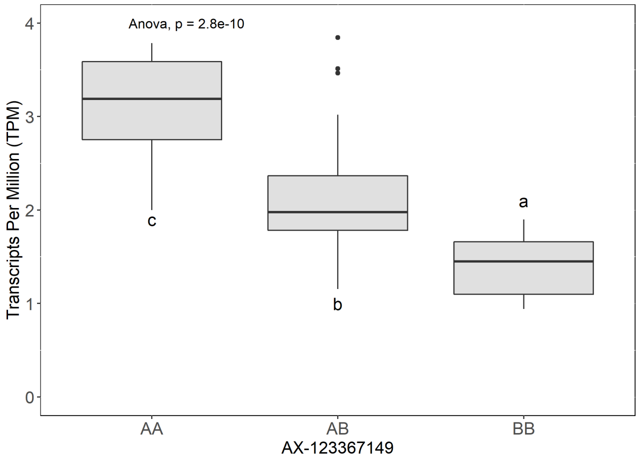

(F3PH) Flavonoid 3 -monooxygenase

augustus_masked-Fvb5-2-processed-gene-78.0 (cis-eQTL)

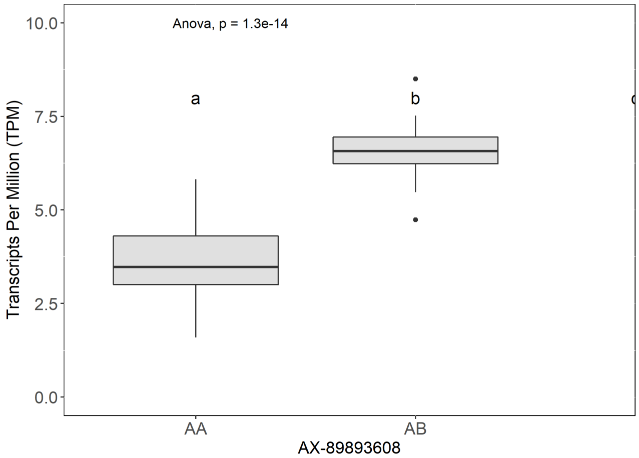

(DFRA) Dihydroflavonol 4-reductase

maker-Fvb2-1-augustus-gene-255.45 (cis-eQTL)

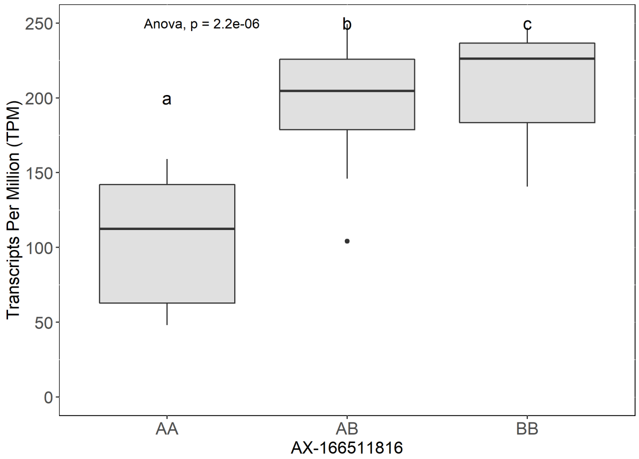

(F16P2) D-fructose-1,6-bisphosphate 1-phosphohydrolase

maker-Fvb2-4-snap-gene-100.35 (cis-eQTL)

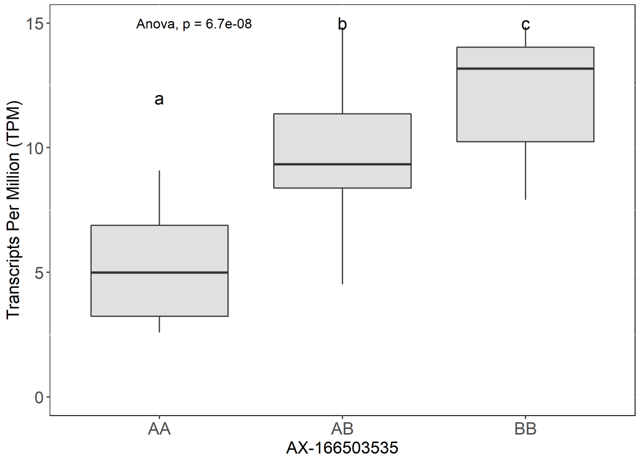

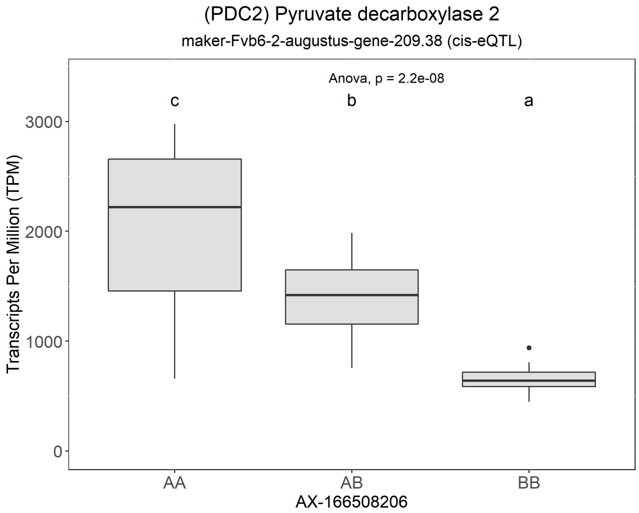
(PDC2) Pyruvate decarboxylase 2
maker-Fvb6-2-augustus-gene-209.38 (cis-eQTL)
Anova, p = 2.2e-08
c
b
a
Transcripts Per Million (TPM)
0
1000
2000
3000
AA
AB
BB
AX-166508206

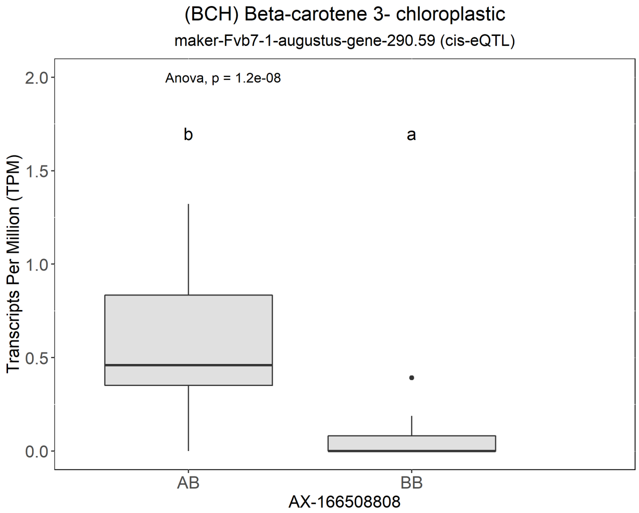
(BCH) Beta-carotene 3- chloroplastic
maker-Fvb7-1-augustus-gene-290.59 (cis-eQTL)
Anova, p = 1.2e-08
b
a
Transcripts Per Million (TPM)
0.0
0.5
1.0
1.5
2.0
AB
BB
AX-166508808

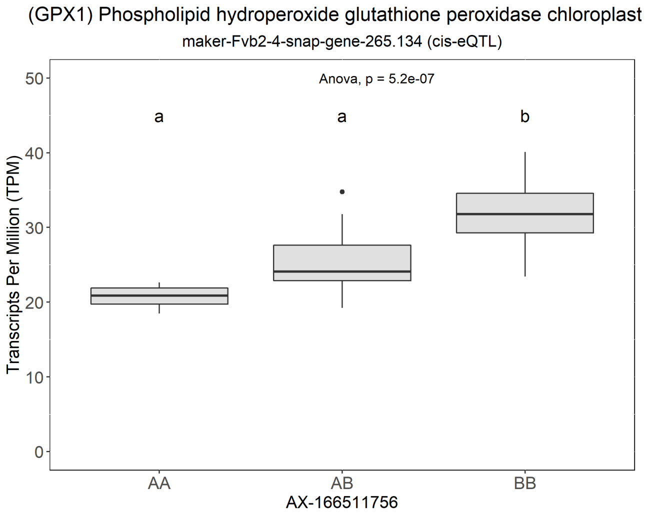
(GPX1) Phospholipid hydroperoxide glutathione peroxidase chloroplast
maker-Fvb2-4-snap-gene-265.134 (cis-eQTL)
Anova, p = 5.2e-07
a
a
b
Transcripts Per Million (TPM)
0
10
20
30
40
50
AA
AB
BB
AX-166511756

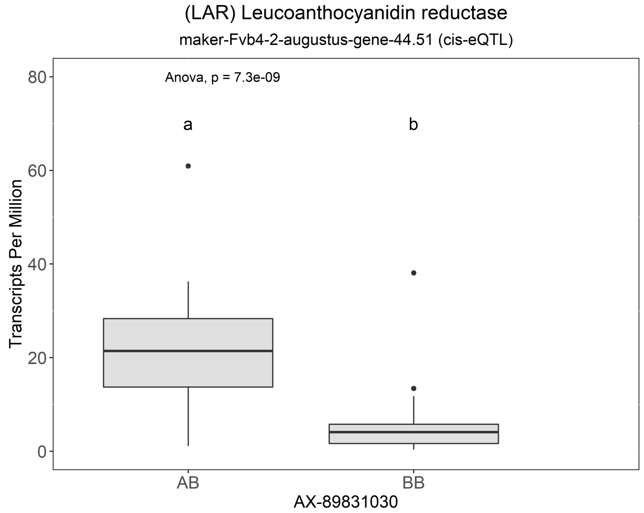
(LAR) Leucoanthocyanidin reductase
maker-Fvb4-2-augustus-gene-44.51 (cis-eQTL)
Anova, p = 7.3e-09
a
b
Transcripts Per Million
0
20
40
60
80
AB
BB
AX-89831030

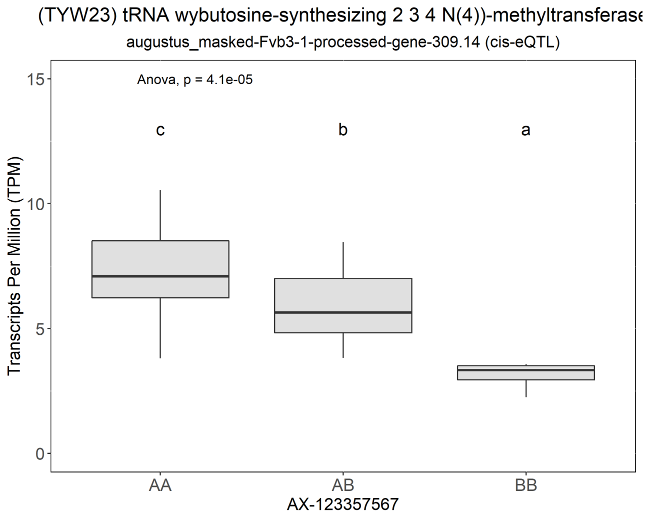
(TYW23) tRNA wybutosine-synthesizing 2 3 4 N(4))-methyltransferase
augustus_masked-Fvb3-1-processed-gene-309.14 (cis-eQTL)
Anova, p = 4.1e-05
c
b
a
Transcripts Per Million (TPM)
0
5
10
15
AA
AB
BB
AX-123357567

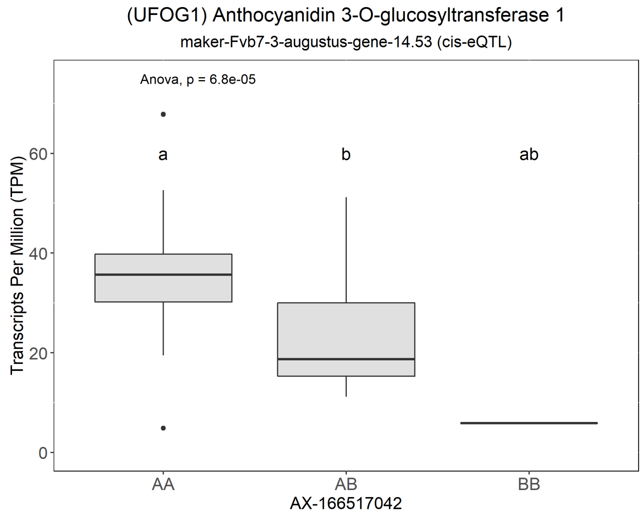
(UFOG1) Anthocyanidin 3-O-glucosyltransferase 1
maker-Fvb7-3-augustus-gene-14.53 (cis-eQTL)
Anova, p = 6.8e-05
a
b
ab
Transcripts Per Million (TPM)
0
20
40
60
AA
AB
BB
AX-166517042

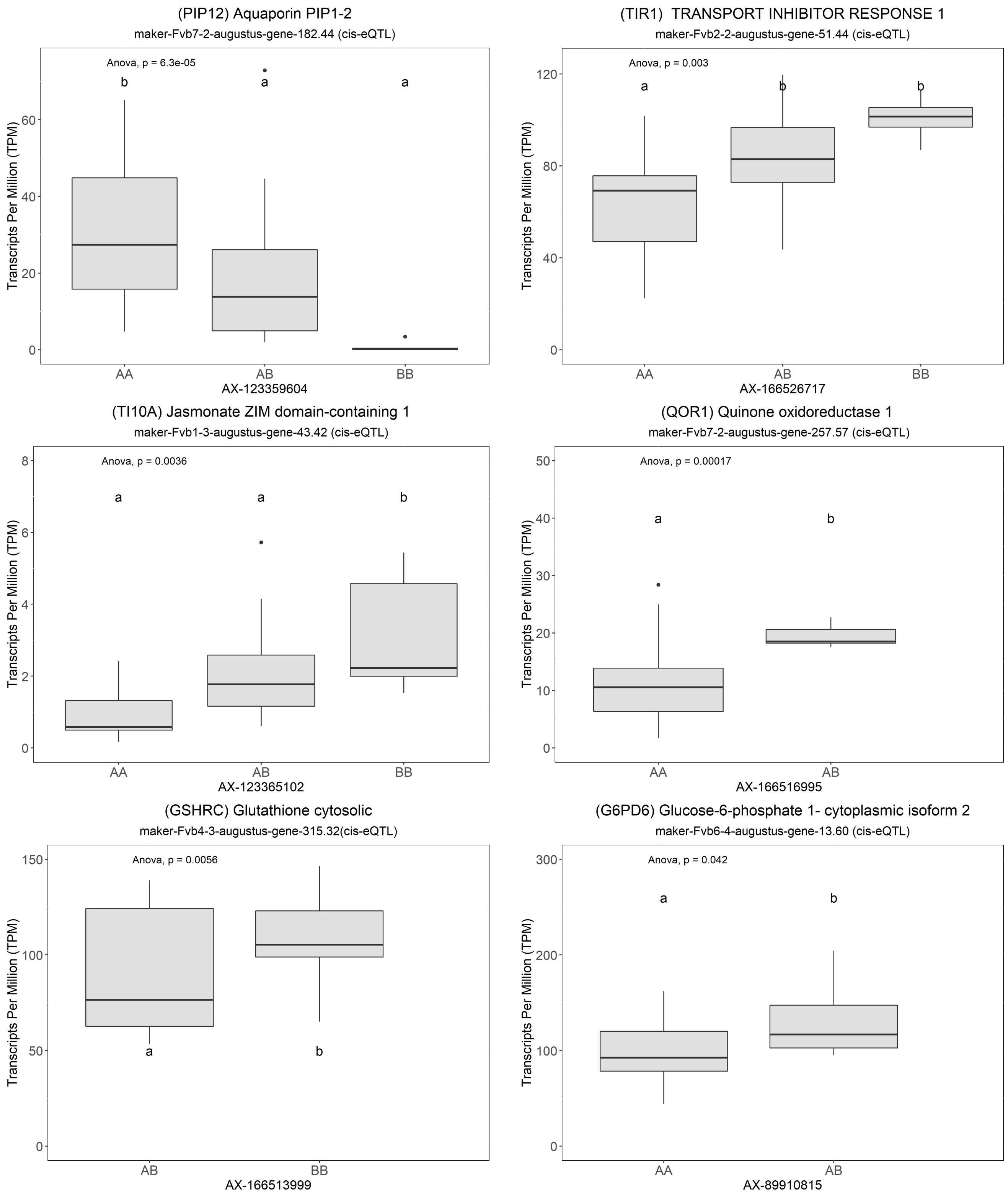

(PIP12) Aquaporin PIP1-2
maker-Fvb7-2-augustus-gene-182.44 (cis-eQTL)
Anova, p = 6.3e-05
b
a
a
Transcripts Per Million (TPM)
0
20
40
60
AA
AB
BB
AX-123359604
(TIR1) TRANSPORT INHIBITOR RESPONSE 1
maker-Fvb2-2-augustus-gene-51.44 (cis-eQTL)
Anova, p = 0.003
a
b
b
Transcripts Per Million (TPM)
0
40
80
120
AA
AB
BB
AX-166526717
(TI10A) Jasmonate ZIM domain-containing 1
maker-Fvb1-3-augustus-gene-43.42 (cis-eQTL)
Anova, p = 0.0036
a
a
b
Transcripts Per Million (TPM)
0
2
4
6
8
AA
AB
BB
AX-123365102
(QOR1) Quinone oxidoreductase 1
maker-Fvb7-2-augustus-gene-257.57 (cis-eQTL)
Anova, p = 0.00017
a
b
Transcripts Per Million (TPM)
0
10
20
30
40
50
AA
AB
AX-166516995
(GSHRC) Glutathione cytosolic
maker-Fvb4-3-augustus-gene-315.32(cis-eQTL)
Anova, p = 0.0056
a
b
Transcripts Per Million (TPM)
0
50
100
150
AB
BB
AX-166513999
(G6PD6) Glucose-6-phosphate 1- cytoplasmic isoform 2
maker-Fvb6-4-augustus-gene-13.60 (cis-eQTL)
Anova, p = 0.042
a
b
Transcripts Per Million (TPM)
0
100
200
300
AA
AB
AX-89910815

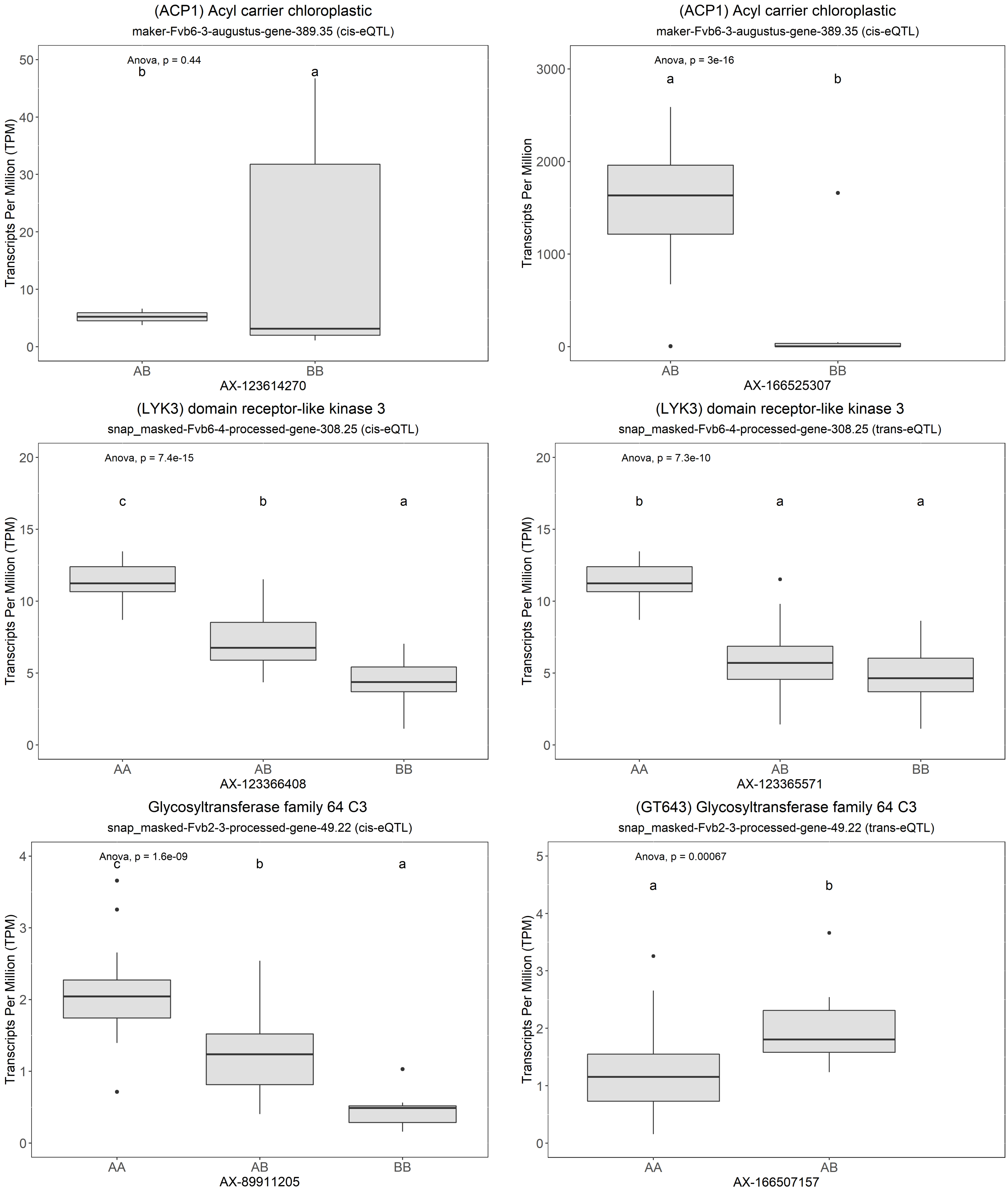

(ACP1) Acyl carrier chloroplastic
maker-Fvb6-3-augustus-gene-389.35 (cis-eQTL)
Anova, p = 0.44
Transcripts Per Million (TPM)
AX-123614270
(ACP1) Acyl carrier chloroplastic
maker-Fvb6-3-augustus-gene-389.35 (cis-eQTL)
Anova, p = 3e-16
Transcripts Per Million
AX-166525307
(LYK3) domain receptor-like kinase 3
snap_masked-Fvb6-4-processed-gene-308.25 (cis-eQTL)
Anova, p = 7.4e-15
Transcripts Per Million (TPM)
AX-123366408
(LYK3) domain receptor-like kinase 3
snap_masked-Fvb6-4-processed-gene-308.25 (trans-eQTL)
Anova, p = 7.3e-10
Transcripts Per Million (TPM)
AX-123365571
Glycosyltransferase family 64 C3
snap_masked-Fvb2-3-processed-gene-49.22 (cis-eQTL)
Anova, p = 1.6e-09
Transcripts Per Million (TPM)
AX-89911205
(GT643) Glycosyltransferase family 64 C3
snap_masked-Fvb2-3-processed-gene-49.22 (trans-eQTL)
Anova, p = 0.00067
Transcripts Per Million (TPM)
AX-166507157

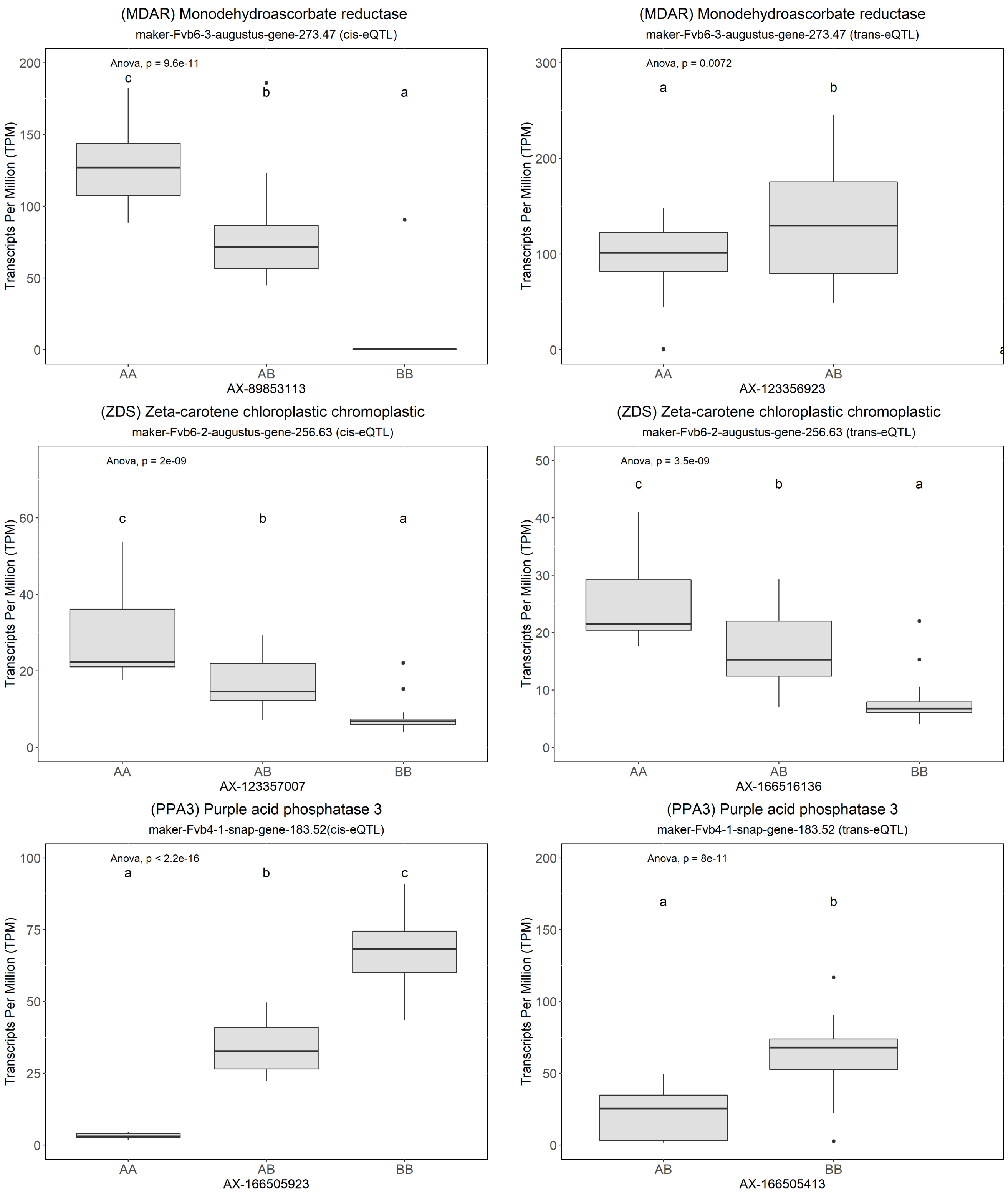
(MDAR) Monodehydroascorbate reductase
maker-Fvb6-3-augustus-gene-273.47 (cis-eQTL)
Anova, p = 9.6e-11
c
b
a
Transcripts Per Million (TPM)
0
50
100
150
200
AA
AB
BB
AX-89853113
(MDAR) Monodehydroascorbate reductase
maker-Fvb6-3-augustus-gene-273.47 (trans-eQTL)
Anova, p = 0.0072
a
b
Transcripts Per Million (TPM)
0
100
200
300
AA
AB
AX-123356923
(ZDS) Zeta-carotene chloroplastic chromoplastic
maker-Fvb6-2-augustus-gene-256.63 (cis-eQTL)
Anova, p = 2e-09
c
b
a
Transcripts Per Million (TPM)
0
20
40
60
AA
AB
BB
AX-123357007
(ZDS) Zeta-carotene chloroplastic chromoplastic
maker-Fvb6-2-augustus-gene-256.63 (trans-eQTL)
Anova, p = 3.5e-09
c
b
a
Transcripts Per Million (TPM)
0
10
20
30
40
50
AA
AB
BB
AX-166516136
(PPA3) Purple acid phosphatase 3
maker-Fvb4-1-snap-gene-183.52(cis-eQTL)
Anova, p < 2.2e-16
a
b
c
Transcripts Per Million (TPM)
0
25
50
75
100
AA
AB
BB
AX-166505923
(PPA3) Purple acid phosphatase 3
maker-Fvb4-1-snap-gene-183.52 (trans-eQTL)
Anova, p = 8e-11
a
b
Transcripts Per Million (TPM)
0
50
100
150
200
AB
BB
AX-166505413

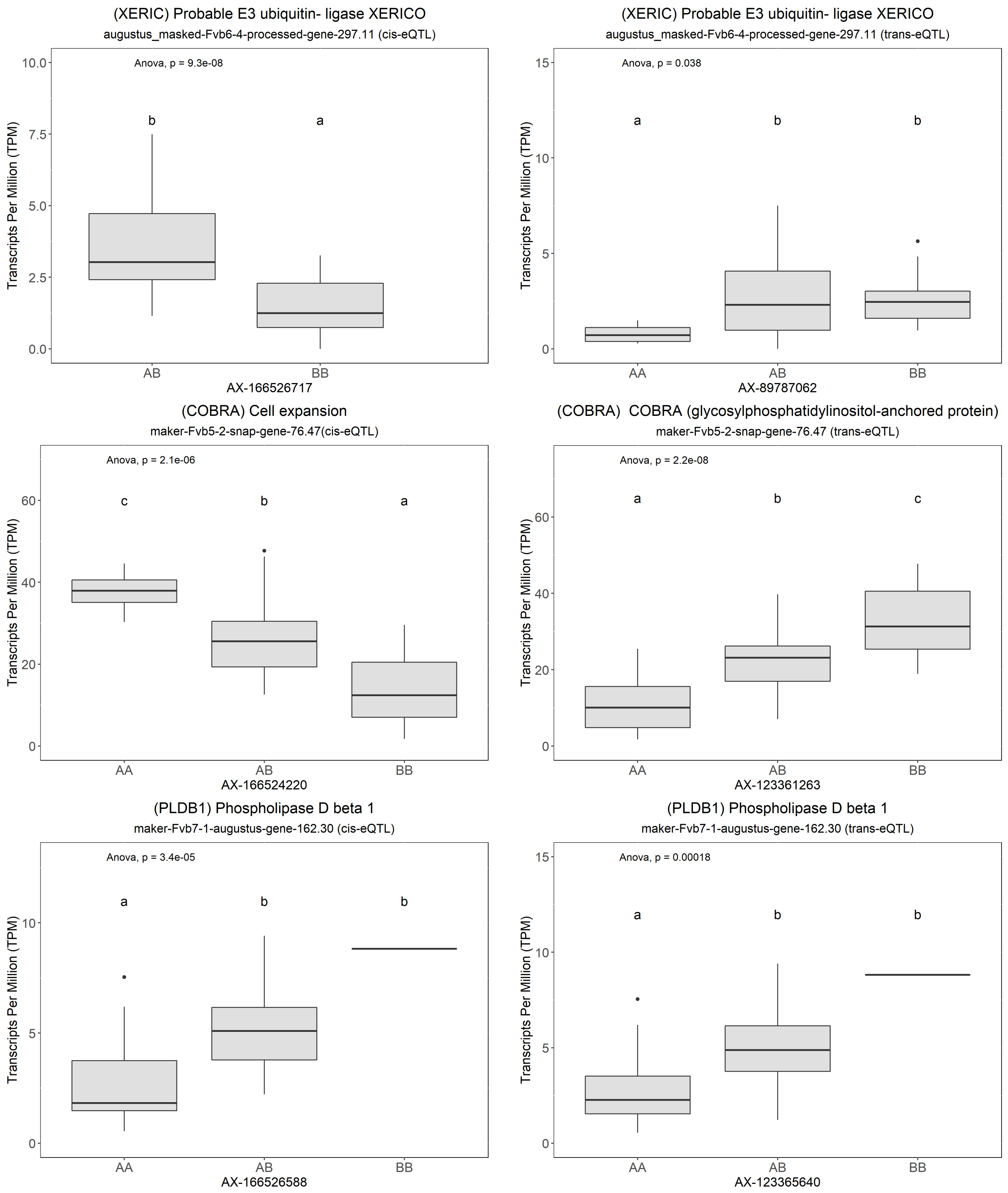
(XERIC) Probable E3 ubiquitin- ligase XERICO
augustus_masked-Fvb6-4-processed-gene-297.11 (cis-eQTL)
Anova, p = 9.3e-08
b
a
Transcripts Per Million (TPM)
10.0
7.5
5.0
2.5
0.0
AB
BB
AX-166526717
(XERIC) Probable E3 ubiquitin- ligase XERICO
augustus_masked-Fvb6-4-processed-gene-297.11 (trans-eQTL)
Anova, p = 0.038
a
b
b
Transcripts Per Million (TPM)
15
10
5
0
AA
AB
BB
AX-89787062
(COBRA) Cell expansion
maker-Fvb5-2-snap-gene-76.47(cis-eQTL)
Anova, p = 2.1e-06
c
b
a
Transcripts Per Million (TPM)
60
40
20
0
AA
AB
BB
AX-166524220
(COBRA) COBRA (glycosylphosphatidylinositol-anchored protein)
maker-Fvb5-2-snap-gene-76.47 (trans-eQTL)
Anova, p = 2.2e-08
a
b
c
Transcripts Per Million (TPM)
60
40
20
0
AA
AB
BB
AX-123361263
(PLDB1) Phospholipase D beta 1
maker-Fvb7-1-augustus-gene-162.30 (cis-eQTL)
Anova, p = 3.4e-05
a
b
b
Transcripts Per Million (TPM)
10
5
0
AA
AB
BB
AX-166526588
(PLDB1) Phospholipase D beta 1
maker-Fvb7-1-augustus-gene-162.30 (trans-eQTL)
Anova, p = 0.00018
a
b
b
Transcripts Per Million (TPM)
15
10
5
0
AA
AB
BB
AX-123365640

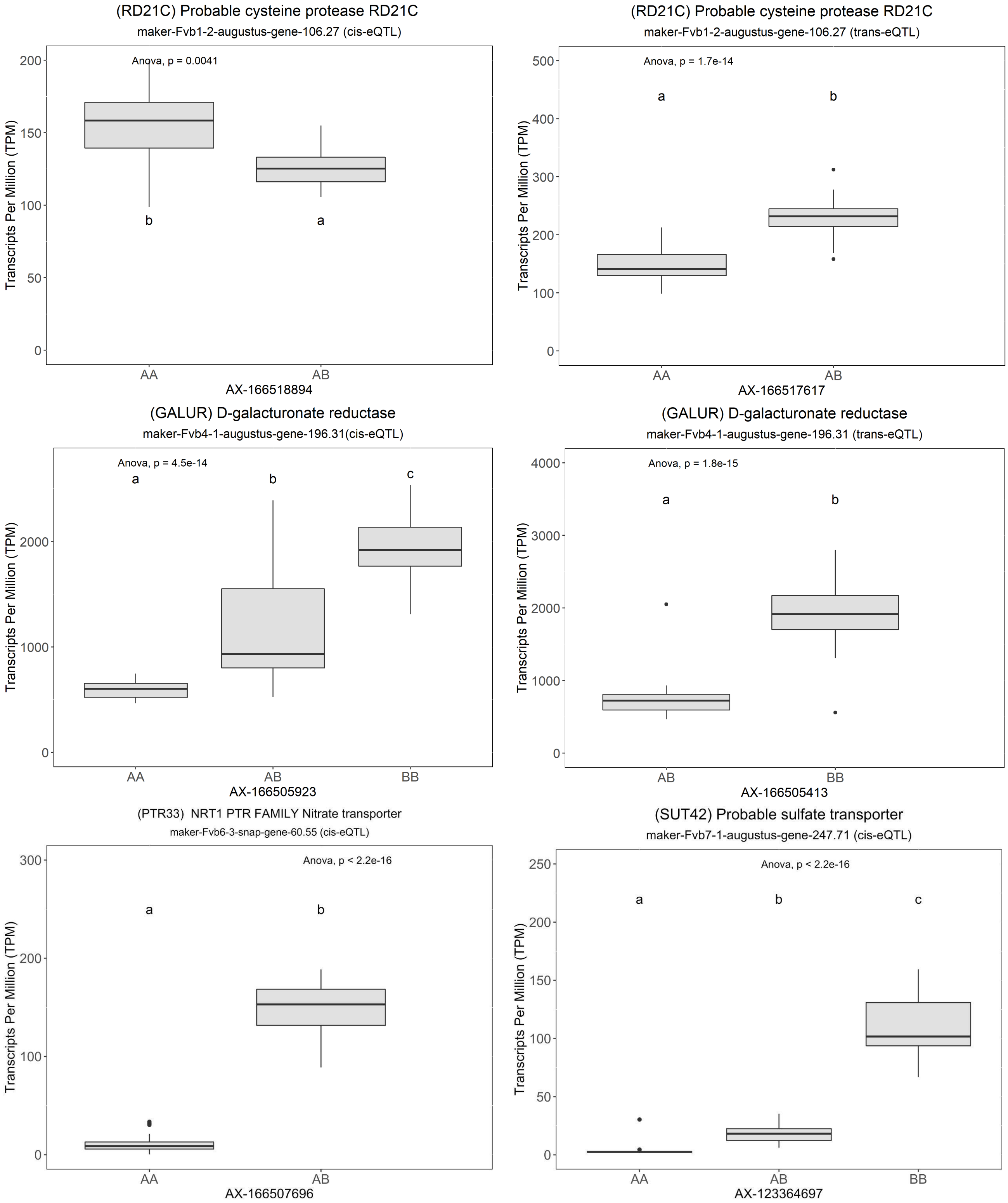
(RD21C) Probable cysteine protease RD21C
maker-Fvb1-2-augustus-gene-106.27 (cis-eQTL)
Anova, p = 0.0041
Transcripts Per Million (TPM)
0
50
100
150
200
b
a
AA
AB
AX-166518894
(RD21C) Probable cysteine protease RD21C
maker-Fvb1-2-augustus-gene-106.27 (trans-eQTL)
Anova, p = 1.7e-14
Transcripts Per Million (TPM)
0
100
200
300
400
500
a
b
AA
AB
AX-166517617
(GALUR) D-galacturonate reductase
maker-Fvb4-1-augustus-gene-196.31(cis-eQTL)
Anova, p = 4.5e-14
Transcripts Per Million (TPM)
0
1000
2000
a
b
c
AA
AB
BB
AX-166505923
(GALUR) D-galacturonate reductase
maker-Fvb4-1-augustus-gene-196.31 (trans-eQTL)
Anova, p = 1.8e-15
Transcripts Per Million (TPM)
0
1000
2000
3000
4000
a
b
AB
BB
AX-166505413
(PTR33) NRT1 PTR FAMILY Nitrate transporter
maker-Fvb6-3-snap-gene-60.55 (cis-eQTL)
Anova, p < 2.2e-16
Transcripts Per Million (TPM)
0
100
200
300
a
b
AA
AB
AX-166507696
(SUT42) Probable sulfate transporter
maker-Fvb7-1-augustus-gene-247.71 (cis-eQTL)
Anova, p < 2.2e-16
Transcripts Per Million (TPM)
0
50
100
150
200
250
a
b
c
AA
AB
BB
AX-123364697

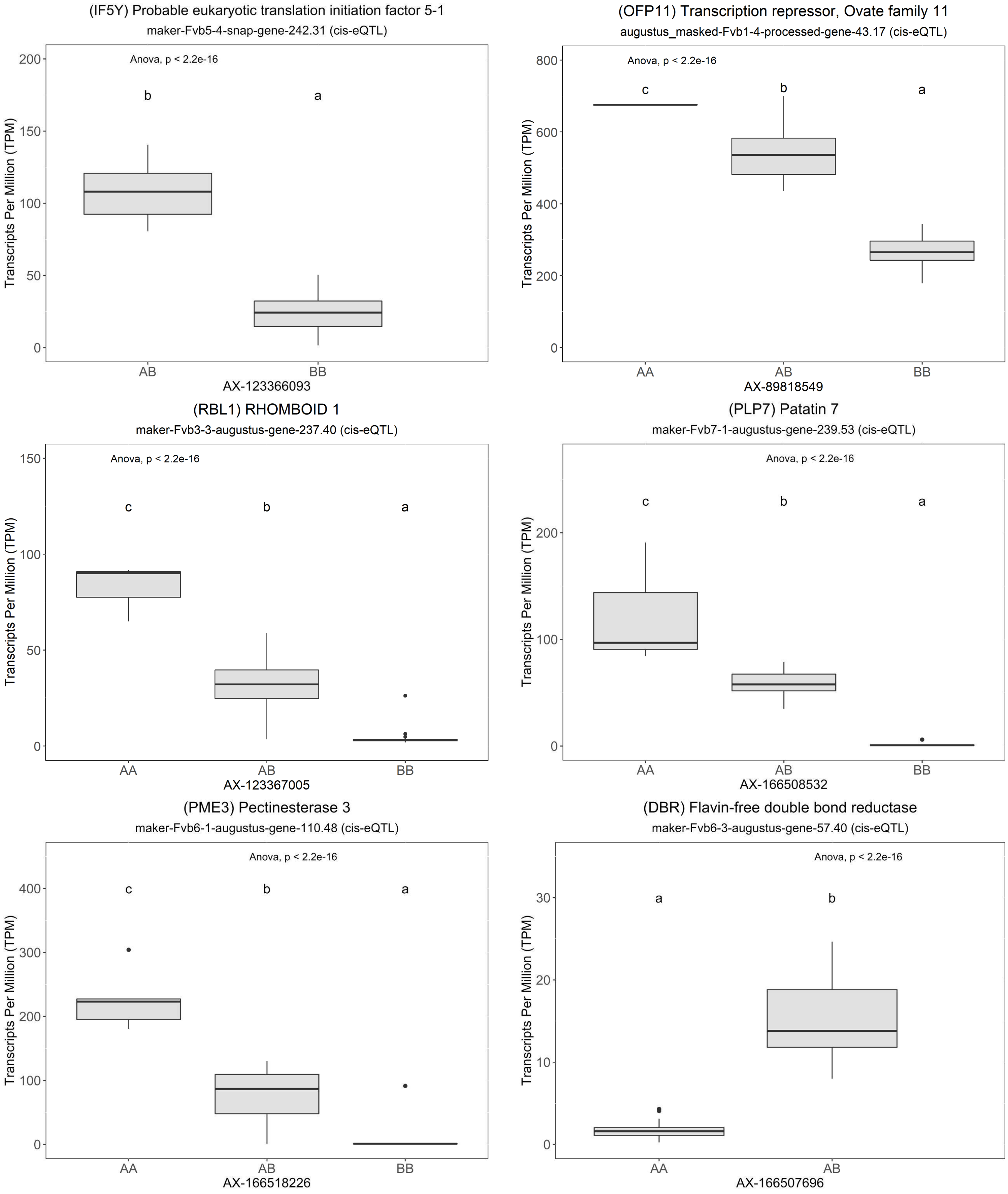
(IF5Y) Probable eukaryotic translation initiation factor 5-1
maker-Fvb5-4-snap-gene-242.31 (cis-eQTL)
Anova, p < 2.2e-16
b
a
Transcripts Per Million (TPM)
0
50
100
150
200
AB
BB
AX-123366093
(OFP11) Transcription repressor, Ovate family 11
augustus_masked-Fvb1-4-processed-gene-43.17 (cis-eQTL)
Anova, p < 2.2e-16
c
b
a
Transcripts Per Million (TPM)
0
200
400
600
800
AA
AB
BB
AX-89818549
(RBL1) RHOMBOID 1
maker-Fvb3-3-augustus-gene-237.40 (cis-eQTL)
Anova, p < 2.2e-16
c
b
a
Transcripts Per Million (TPM)
0
50
100
150
AA
AB
BB
AX-123367005
(PLP7) Patatin 7
maker-Fvb7-1-augustus-gene-239.53 (cis-eQTL)
Anova, p < 2.2e-16
c
b
a
Transcripts Per Million (TPM)
0
100
200
AA
AB
BB
AX-166508532
(PME3) Pectinesterase 3
maker-Fvb6-1-augustus-gene-110.48 (cis-eQTL)
Anova, p < 2.2e-16
c
b
a
Transcripts Per Million (TPM)
0
100
200
300
400
AA
AB
BB
AX-166518226
(DBR) Flavin-free double bond reductase
maker-Fvb6-3-augustus-gene-57.40 (cis-eQTL)
Anova, p < 2.2e-16
a
b
Transcripts Per Million (TPM)
0
10
20
30
AA
AB
AX-166507696

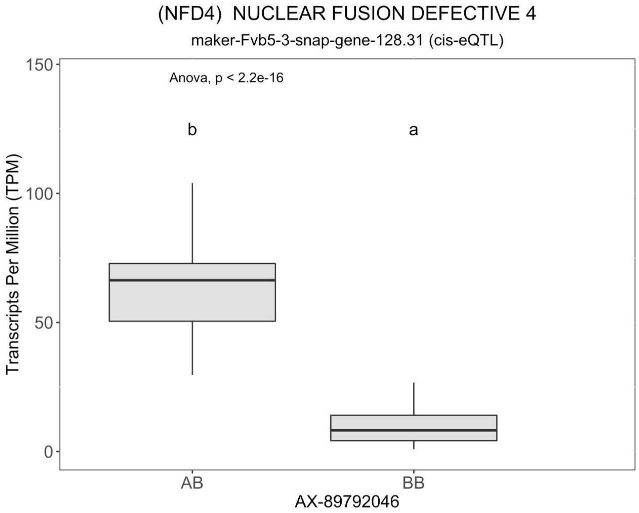
(NFD4) NUCLEAR FUSION DEFECTIVE 4
maker-Fvb5-3-snap-gene-128.31 (cis-eQTL)
Anova, p < 2.2e-16
b
a
Transcripts Per Million (TPM)
150
100
50
0
AB
BB
AX-89792046

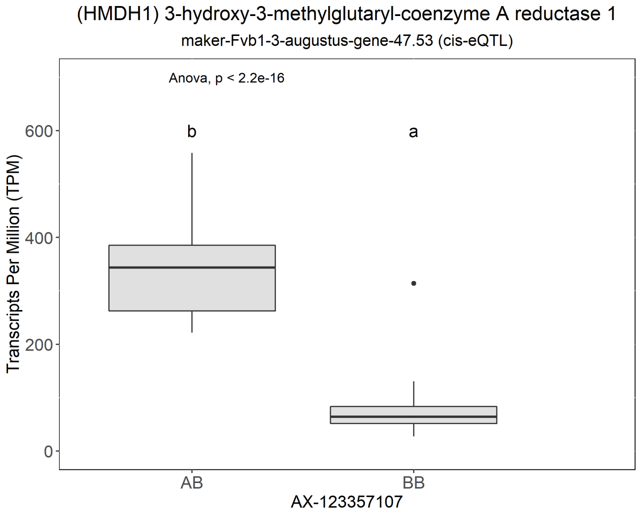
(HMDH1) 3-hydroxy-3-methylglutaryl-coenzyme A reductase 1
maker-Fvb1-3-augustus-gene-47.53 (cis-eQTL)
Anova, p < 2.2e-16
b
a
Transcripts Per Million (TPM)
600
400
200
0
AB
BB
AX-123357107

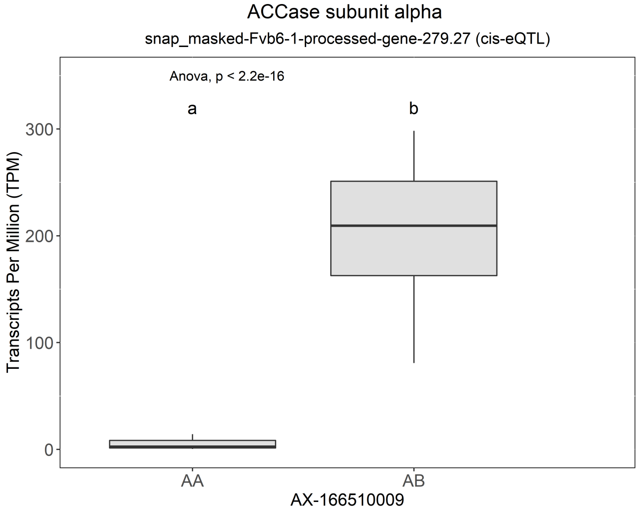
ACCase subunit alpha
snap_masked-Fvb6-1-processed-gene-279.27 (cis-eQTL)
Anova, p < 2.2e-16
a
b
Transcripts Per Million (TPM)
300
200
100
0
AA
AB
AX-166510009

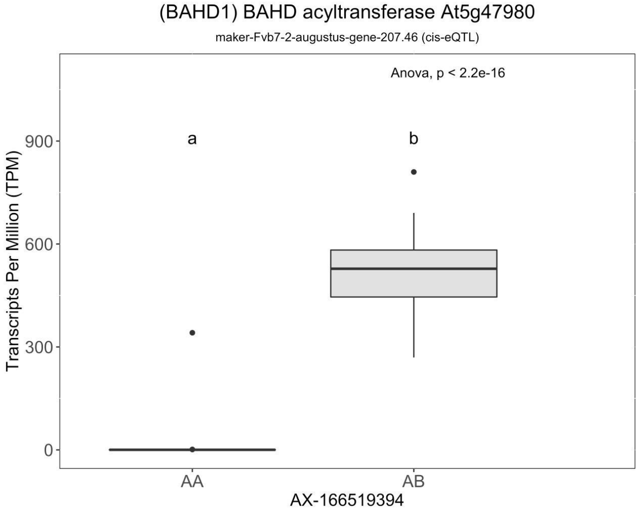
(BAHD1) BAHD acyltransferase At5g47980
maker-Fvb7-2-augustus-gene-207.46 (cis-eQTL)
Anova, p < 2.2e-16
a
b
Transcripts Per Million (TPM)
900
600
300
0
AA
AB
AX-166519394

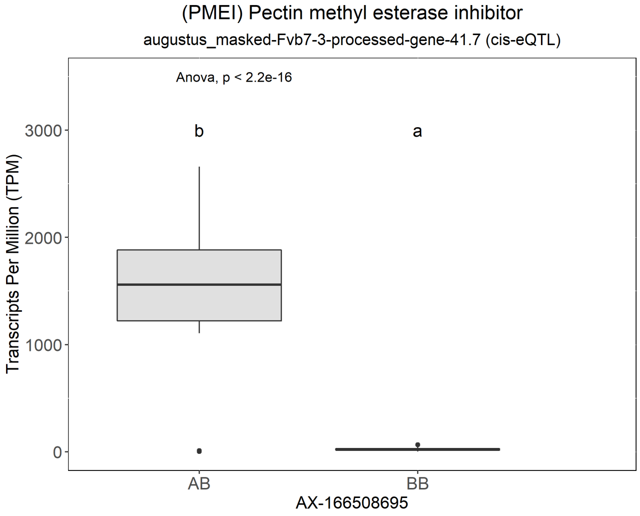
(PMEI) Pectin methyl esterase inhibitor
augustus_masked-Fvb7-3-processed-gene-41.7 (cis-eQTL)
Anova, p < 2.2e-16
b
a
Transcripts Per Million (TPM)
3000
2000
1000
0
AB
BB
AX-166508695

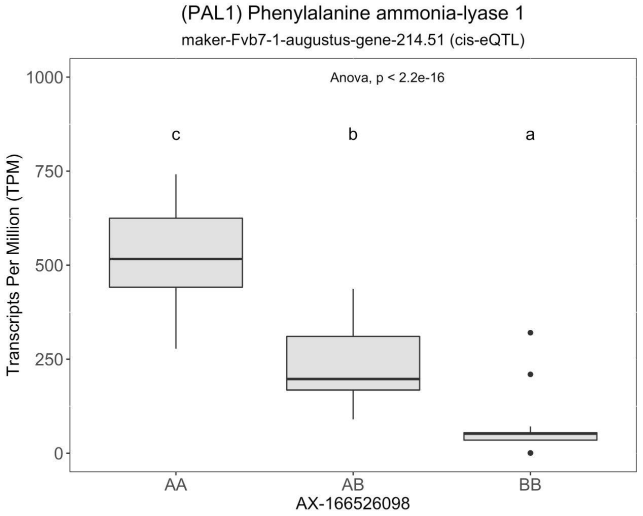
(PAL1) Phenylalanine ammonia-lyase 1
maker-Fvb7-1-augustus-gene-214.51 (cis-eQTL)
Anova, p < 2.2e-16
c
b
a
Transcripts Per Million (TPM)
1000
750
500
250
0
AA
AB
BB
AX-166526098

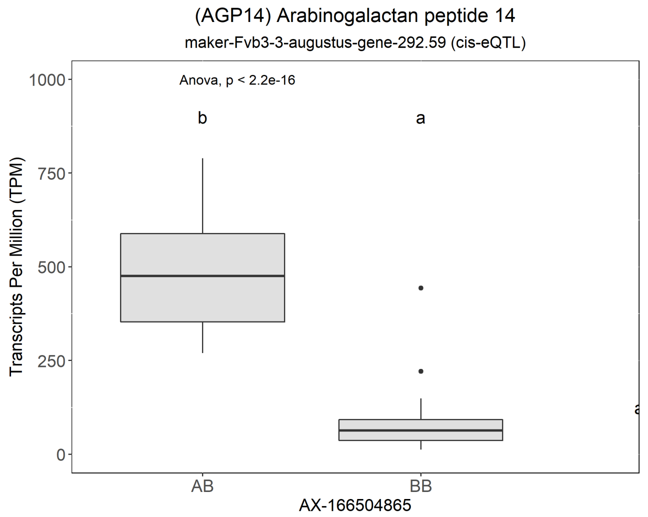
(AGP14) Arabinogalactan peptide 14
maker-Fvb3-3-augustus-gene-292.59 (cis-eQTL)
Anova, p < 2.2e-16
b
a
Transcripts Per Million (TPM)
0
250
500
750
1000
AB
BB
AX-166504865

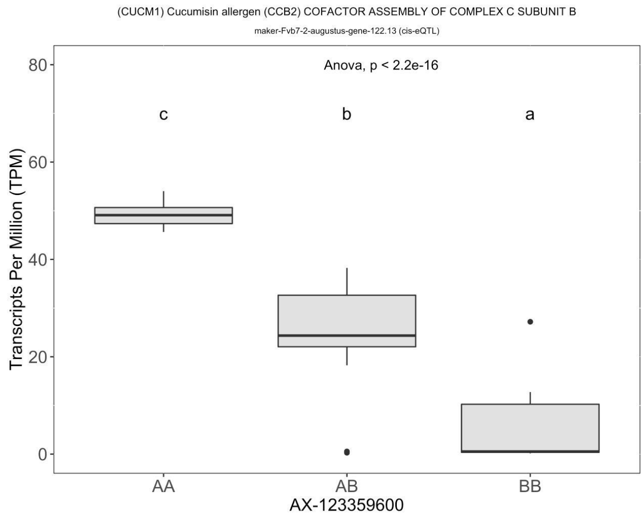
(CUCM1) Cucumisin allergen (CCB2) COFACTOR ASSEMBLY OF COMPLEX C SUBUNIT B
maker-Fvb7-2-augustus-gene-122.13 (cis-eQTL)
Anova, p < 2.2e-16
c
b
a
Transcripts Per Million (TPM)
0
20
40
60
80
AA
AB
BB
AX-123359600

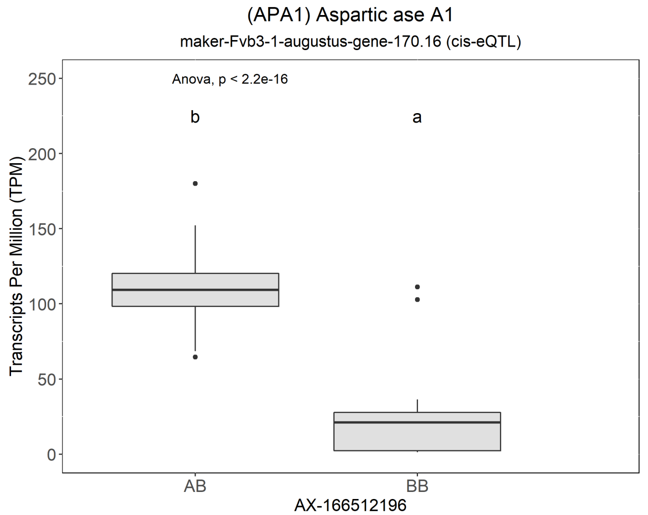
(APA1) Aspartic ase A1
maker-Fvb3-1-augustus-gene-170.16 (cis-eQTL)
Anova, p < 2.2e-16
b
a
Transcripts Per Million (TPM)
0
50
100
150
200
250
AB
BB
AX-166512196

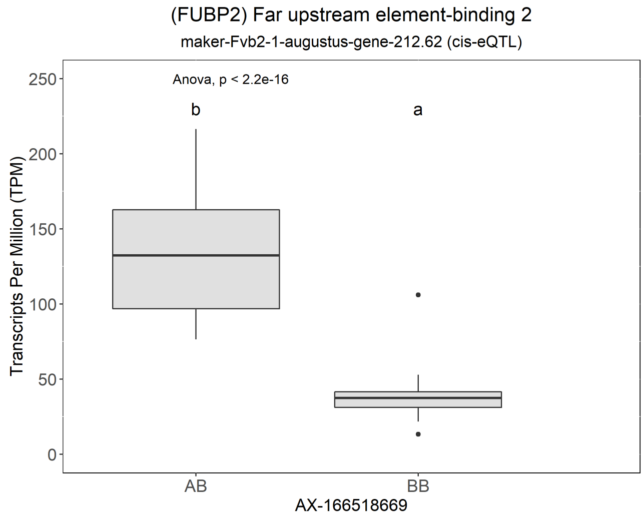
(FUBP2) Far upstream element-binding 2
maker-Fvb2-1-augustus-gene-212.62 (cis-eQTL)
Anova, p < 2.2e-16
b
a
Transcripts Per Million (TPM)
0
50
100
150
200
250
AB
BB
AX-166518669

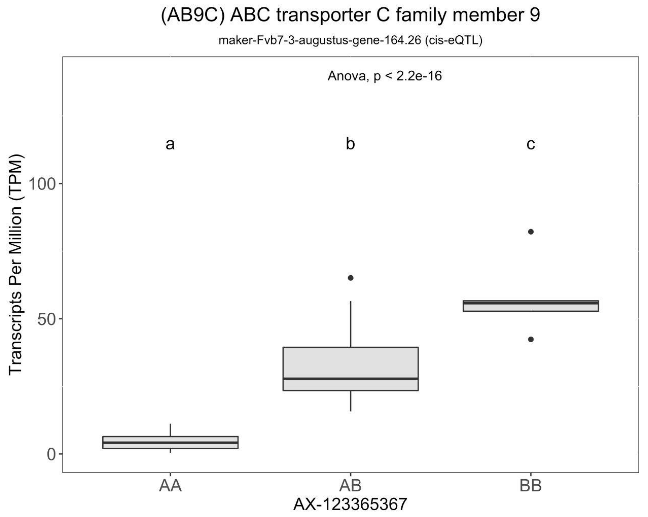
(AB9C) ABC transporter C family member 9
maker-Fvb7-3-augustus-gene-164.26 (cis-eQTL)
Anova, p < 2.2e-16
a
b
c
Transcripts Per Million (TPM)
0
50
100
AA
AB
BB
AX-123365367

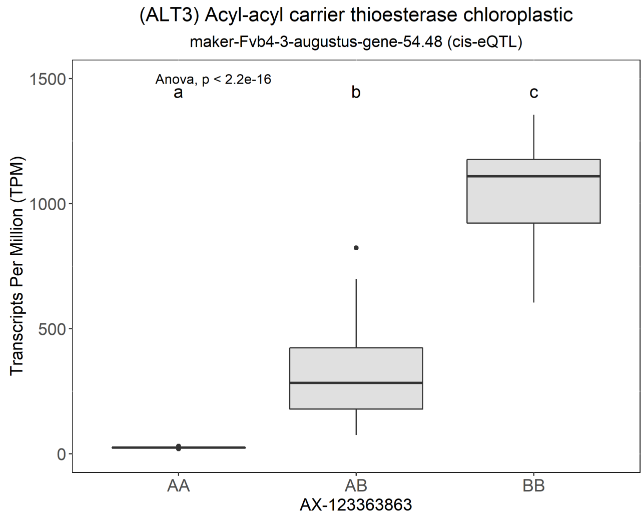
(ALT3) Acyl-acyl carrier thioesterase chloroplastic
maker-Fvb4-3-augustus-gene-54.48 (cis-eQTL)
Anova, p < 2.2e-16
a
b
c
Transcripts Per Million (TPM)
0
500
1000
1500
AA
AB
BB
AX-123363863

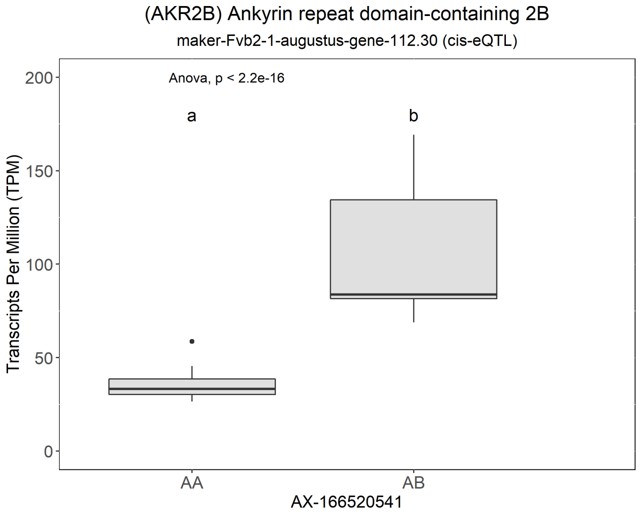
(AKR2B) Ankyrin repeat domain-containing 2B
maker-Fvb2-1-augustus-gene-112.30 (cis-eQTL)
Anova, p < 2.2e-16
a
b
Transcripts Per Million (TPM)
0
50
100
150
200
AA
AB
AX-166520541

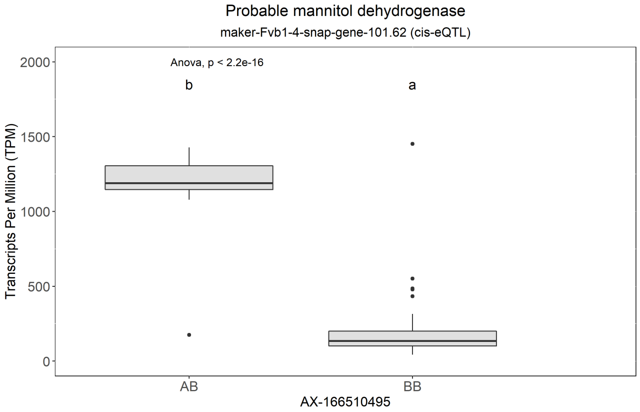
Probable mannitol dehydrogenase
maker-Fvb1-4-snap-gene-101.62 (cis-eQTL)
Anova, p < 2.2e-16
b
a
Transcripts Per Million (TPM)
0
500
1000
1500
2000
AB
BB
AX-166510495

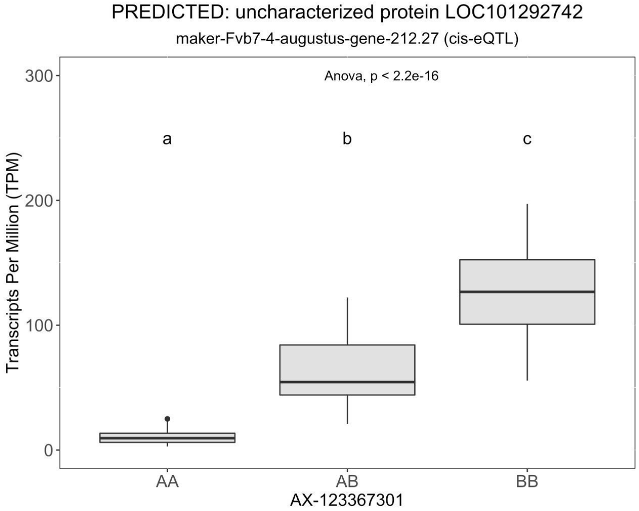
PREDICTED: uncharacterized protein LOC101292742
maker-Fvb7-4-augustus-gene-212.27 (cis-eQTL)
Anova, p < 2.2e-16
a
b
c
Transcripts Per Million (TPM)
0
100
200
300
AA
AB
BB
AX-123367301

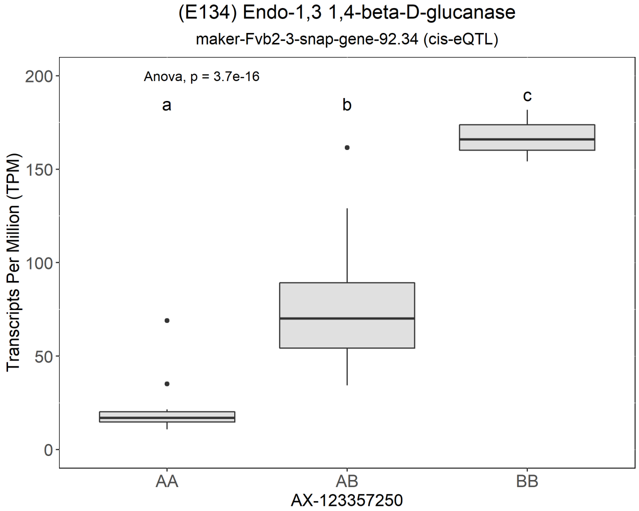
(E134) Endo-1,3 1,4-beta-D-glucanase
maker-Fvb2-3-snap-gene-92.34 (cis-eQTL)
Anova, p = 3.7e-16
a
b
c
Transcripts Per Million (TPM)
0
50
100
150
200
AA
AB
BB
AX-123357250

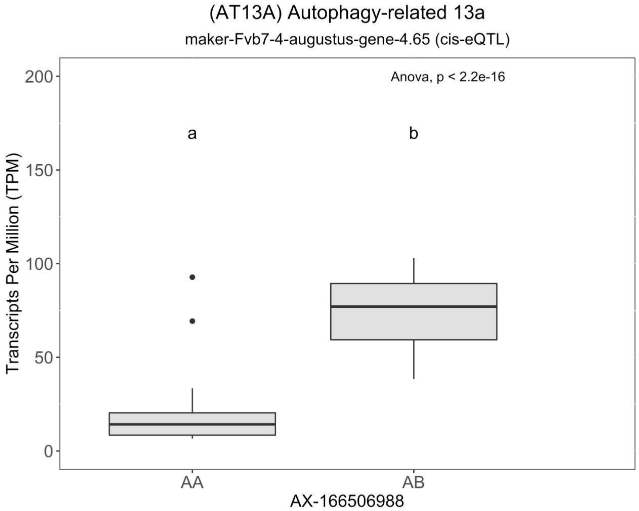
(AT13A) Autophagy-related 13a
maker-Fvb7-4-augustus-gene-4.65 (cis-eQTL)
Anova, p < 2.2e-16
a
b
Transcripts Per Million (TPM)
0
50
100
150
200
AA
AB
AX-166506988

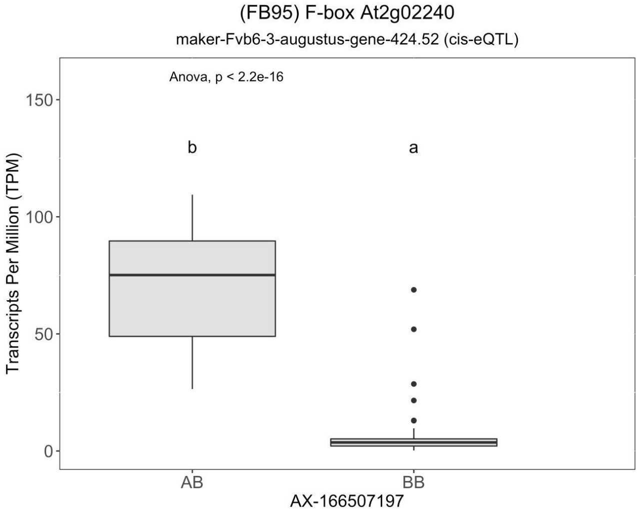
(FB95) F-box At2g02240
maker-Fvb6-3-augustus-gene-424.52 (cis-eQTL)
Anova, p < 2.2e-16
b
a
Transcripts Per Million (TPM)
0
50
100
150
AB
BB
AX-166507197

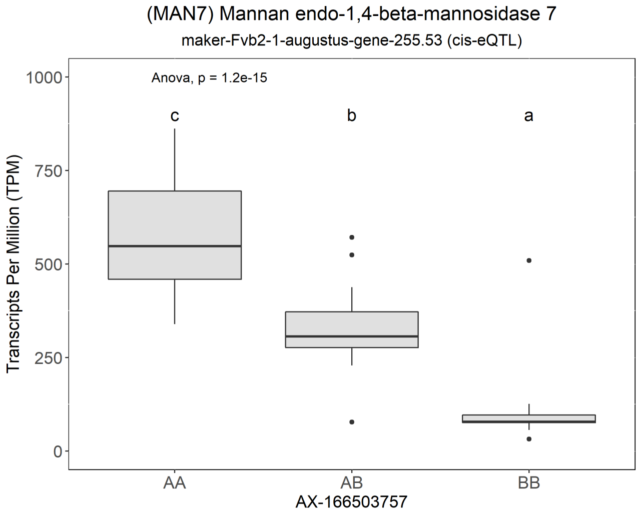
(MAN7) Mannan endo-1,4-beta-mannosidase 7
maker-Fvb2-1-augustus-gene-255.53 (cis-eQTL)
Anova, p = 1.2e-15
c
b
a
Transcripts Per Million (TPM)
1000
750
500
250
0
AA
AB
BB
AX-166503757

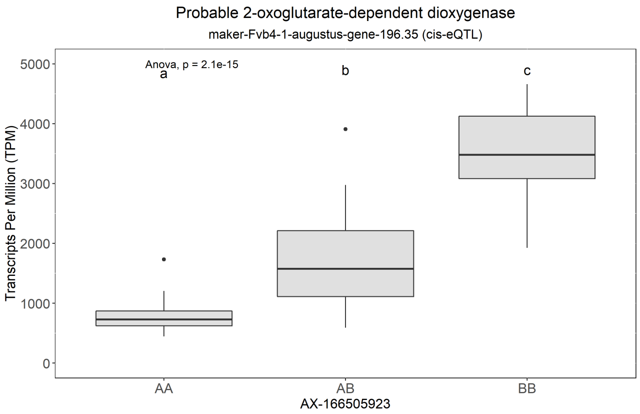
Probable 2-oxoglutarate-dependent dioxygenase
maker-Fvb4-1-augustus-gene-196.35 (cis-eQTL)
Anova, p = 2.1e-15
a
b
c
Transcripts Per Million (TPM)
5000
4000
3000
2000
1000
0
AA
AB
BB
AX-166505923

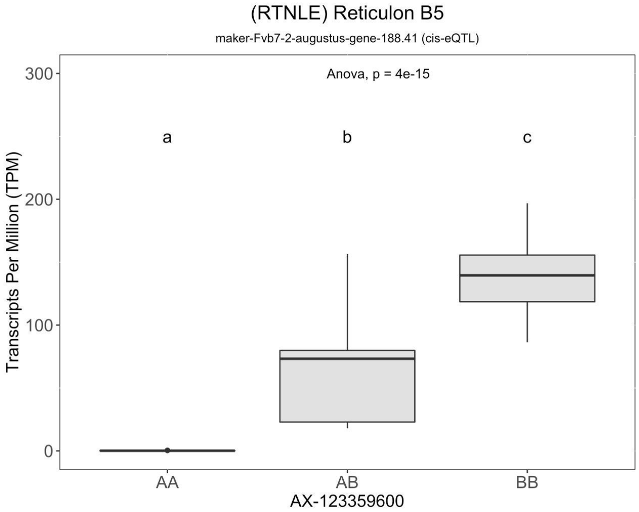
(RTNLE) Reticulon B5
maker-Fvb7-2-augustus-gene-188.41 (cis-eQTL)
Anova, p = 4e-15
a
b
c
Transcripts Per Million (TPM)
300
200
100
0
AA
AB
BB
AX-123359600

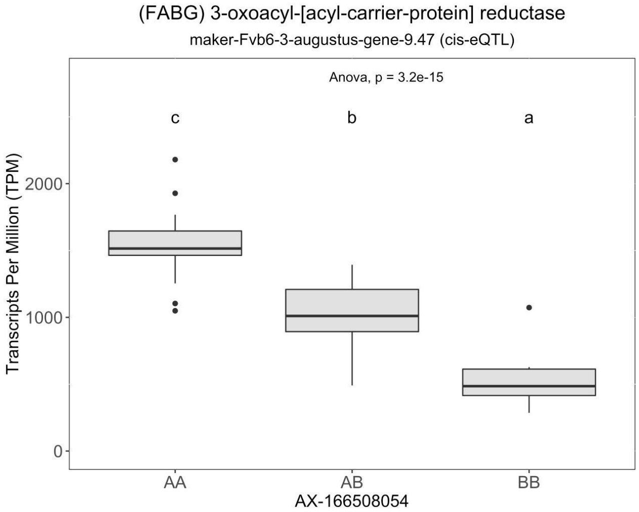
(FABG) 3-oxoacyl-[acyl-carrier-protein] reductase
maker-Fvb6-3-augustus-gene-9.47 (cis-eQTL)
Anova, p = 3.2e-15
c
b
a
Transcripts Per Million (TPM)
2000
1000
0
AA
AB
BB
AX-166508054

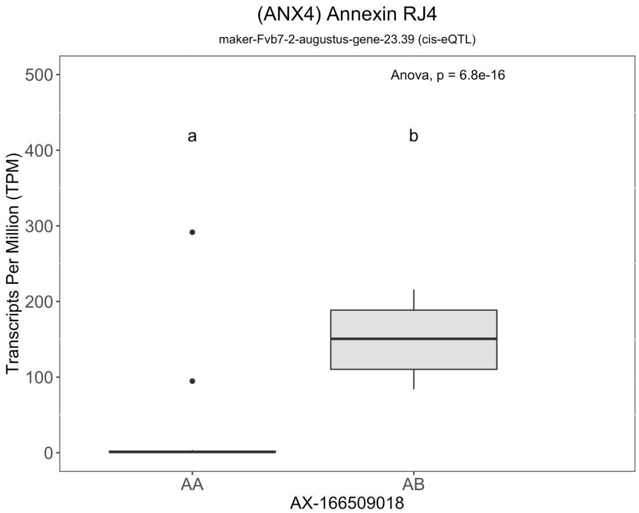
(ANX4) Annexin RJ4
maker-Fvb7-2-augustus-gene-23.39 (cis-eQTL)
Anova, p = 6.8e-16
a
b
Transcripts Per Million (TPM)
500
400
300
200
100
0
AA
AB
AX-166509018

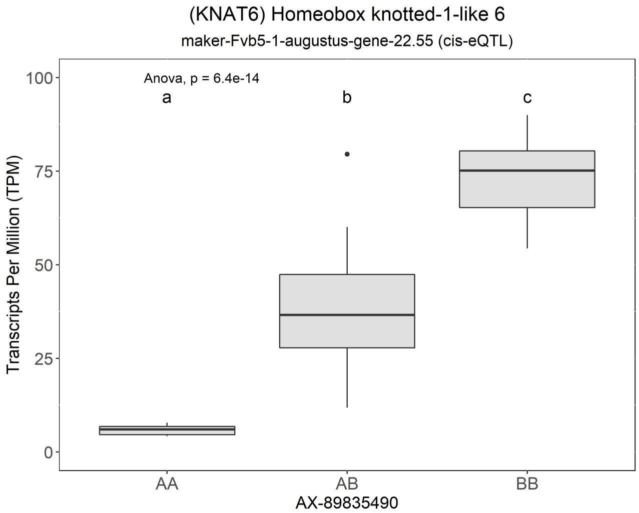
(KNAT6) Homeobox knotted-1-like 6
maker-Fvb5-1-augustus-gene-22.55 (cis-eQTL)
Anova, p = 6.4e-14
a
b
c
Transcripts Per Million (TPM)
100
75
50
25
0
AA
AB
BB
AX-89835490

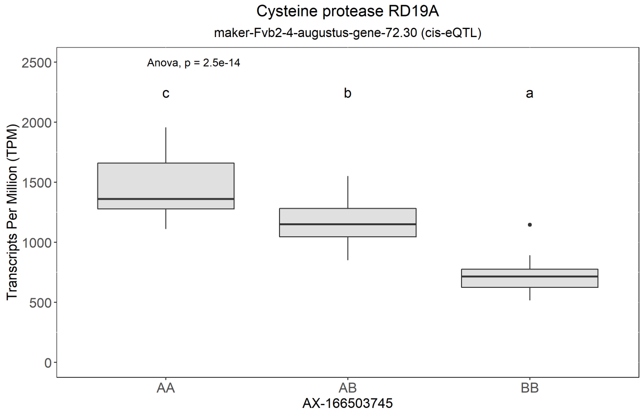
Cysteine protease RD19A
maker-Fvb2-4-augustus-gene-72.30 (cis-eQTL)
Anova, p = 2.5e-14
c
b
a
Transcripts Per Million (TPM)
2500
2000
1500
1000
500
0
AA
AB
BB
AX-166503745

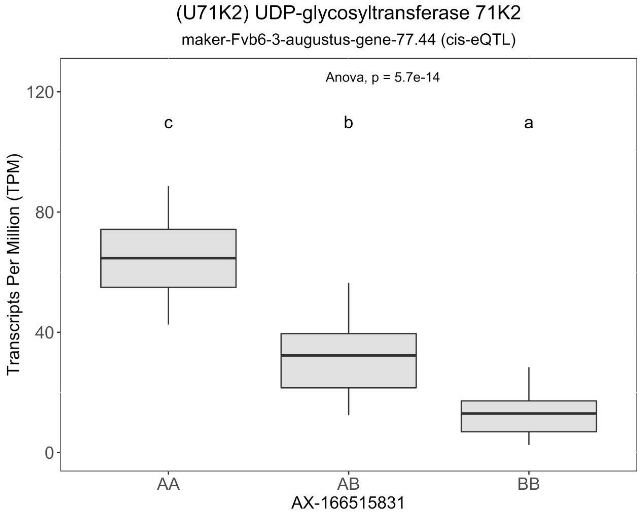
(U71K2) UDP-glycosyltransferase 71K2
maker-Fvb6-3-augustus-gene-77.44 (cis-eQTL)
Anova, p = 5.7e-14
c
b
a
Transcripts Per Million (TPM)
120
80
40
0
AA
AB
BB
AX-166515831

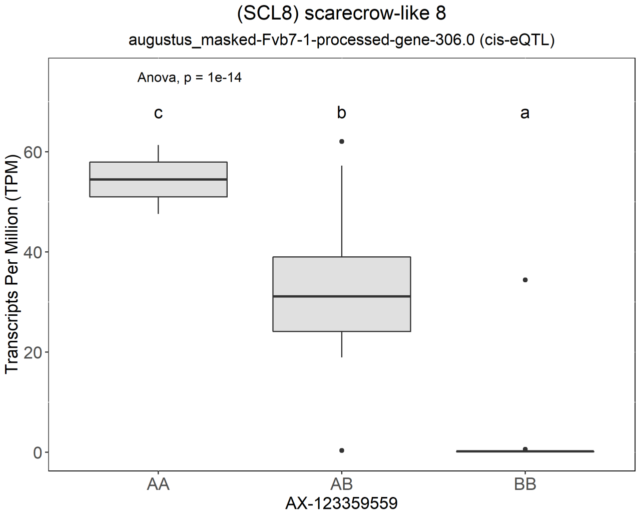
(SCL8) scarecrow-like 8
augustus_masked-Fvb7-1-processed-gene-306.0 (cis-eQTL)
Anova, p = 1e-14
c
b
a
Transcripts Per Million (TPM)
60
40
20
0
AA
AB
BB
AX-123359559

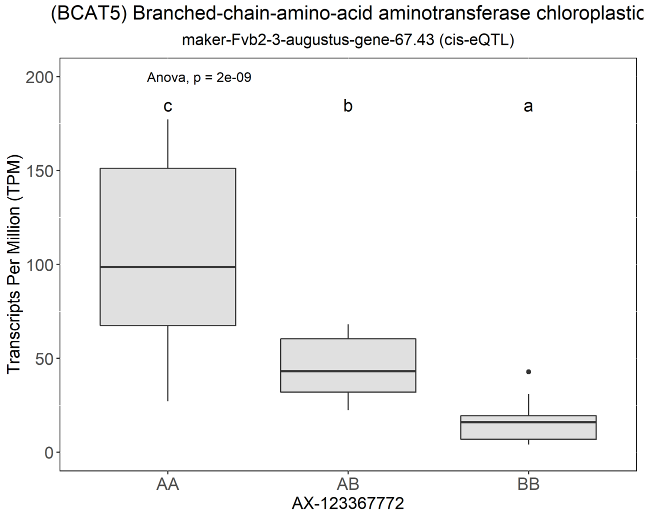
(BCAT5) Branched-chain-amino-acid aminotransferase chloroplastic
maker-Fvb2-3-augustus-gene-67.43 (cis-eQTL)
Anova, p = 2e-09
c
b
a
Transcripts Per Million (TPM)
200
150
100
50
0
AA
AB
BB
AX-123367772

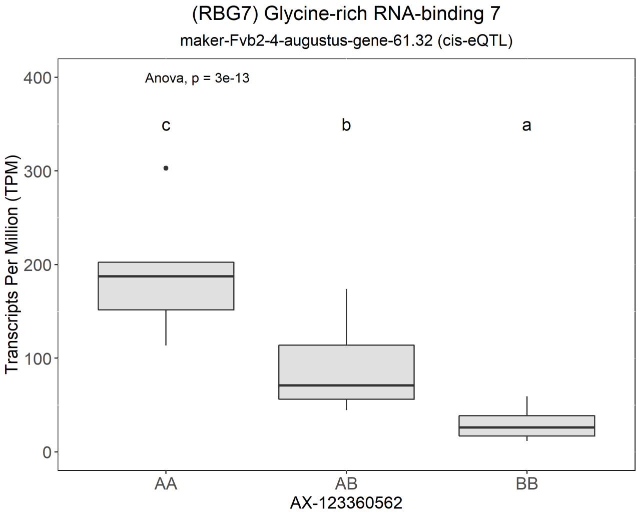
(RBG7) Glycine-rich RNA-binding 7
maker-Fvb2-4-augustus-gene-61.32 (cis-eQTL)
Anova, p = 3e-13
c
b
a
Transcripts Per Million (TPM)
400
300
200
100
0
AA
AB
BB
AX-123360562

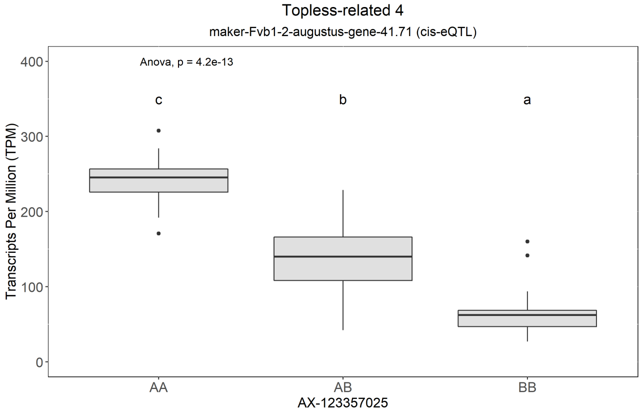
Topless-related 4
maker-Fvb1-2-augustus-gene-41.71 (cis-eQTL)
Anova, p = 4.2e-13
c
b
a
Transcripts Per Million (TPM)
400
300
200
100
0
AA
AB
BB
AX-123357025

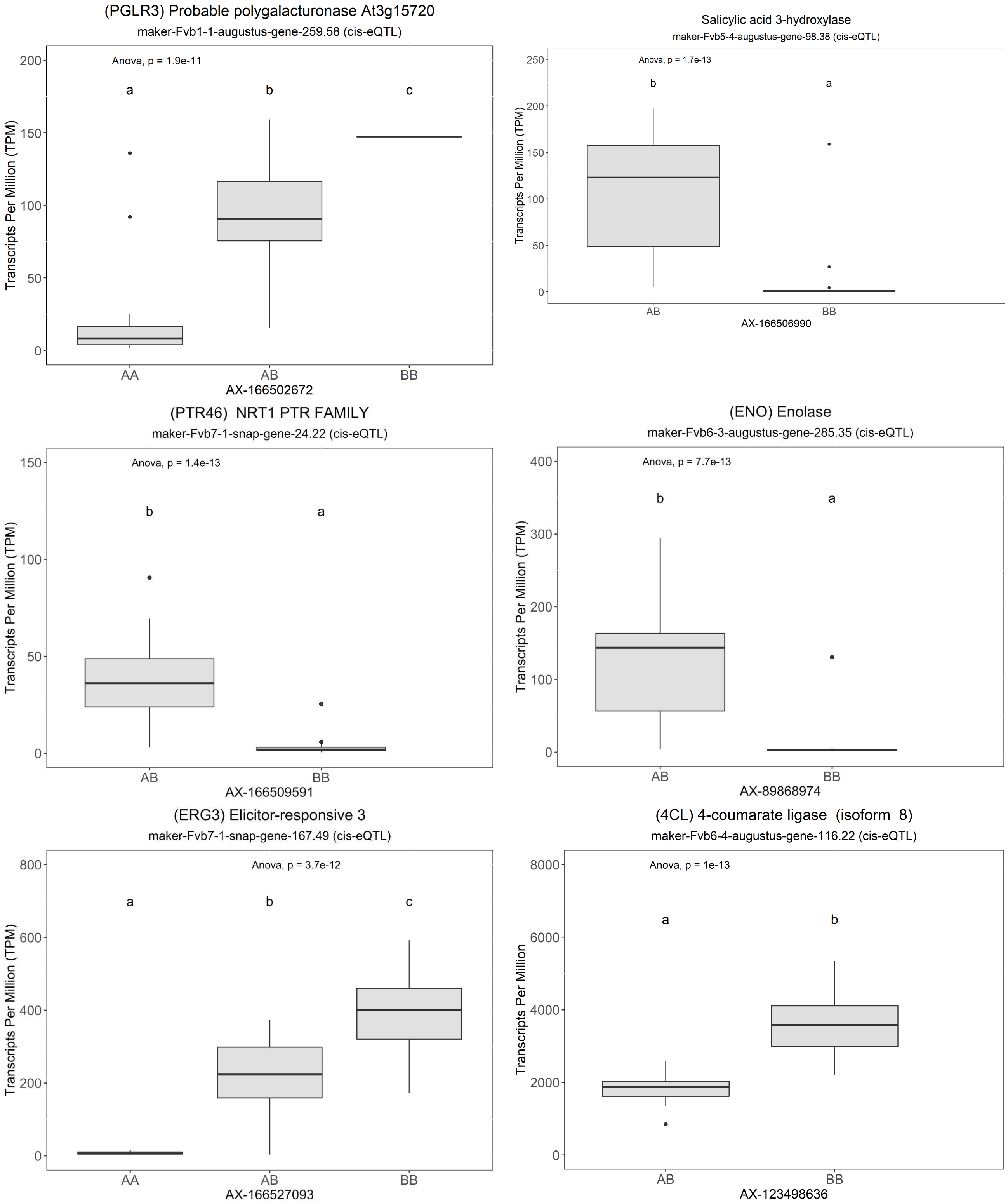

(PGLR3) Probable polygalacturonase At3g15720
maker-Fvb1-1-augustus-gene-259.58 (cis-eQTL)
Anova, p = 1.9e-11
a
b
c
Transcripts Per Million (TPM)
0
50
100
150
200
AA
AB
BB
AX-166502672
Salicylic acid 3-hydroxylase
maker-Fvb5-4-augustus-gene-98.38 (cis-eQTL)
Anova, p = 1.7e-13
b
a
Transcripts Per Million (TPM)
0
50
100
150
200
250
AB
BB
AX-166506990
(PTR46) NRT1 PTR FAMILY
maker-Fvb7-1-snap-gene-24.22 (cis-eQTL)
Anova, p = 1.4e-13
b
a
Transcripts Per Million (TPM)
0
50
100
150
AB
BB
AX-166509591
(ENO) Enolase
maker-Fvb6-3-augustus-gene-285.35 (cis-eQTL)
Anova, p = 7.7e-13
b
a
Transcripts Per Million (TPM)
0
100
200
300
400
AB
BB
AX-89868974
(ERG3) Elicitor-responsive 3
maker-Fvb7-1-snap-gene-167.49 (cis-eQTL)
Anova, p = 3.7e-12
a
b
c
Transcripts Per Million (TPM)
0
200
400
600
800
AA
AB
BB
AX-166527093
(4CL) 4-coumarate ligase (isoform 8)
maker-Fvb6-4-augustus-gene-116.22 (cis-eQTL)
Anova, p = 1e-13
a
b
Transcripts Per Million
0
2000
4000
6000
8000
AB
BB
AX-123498636

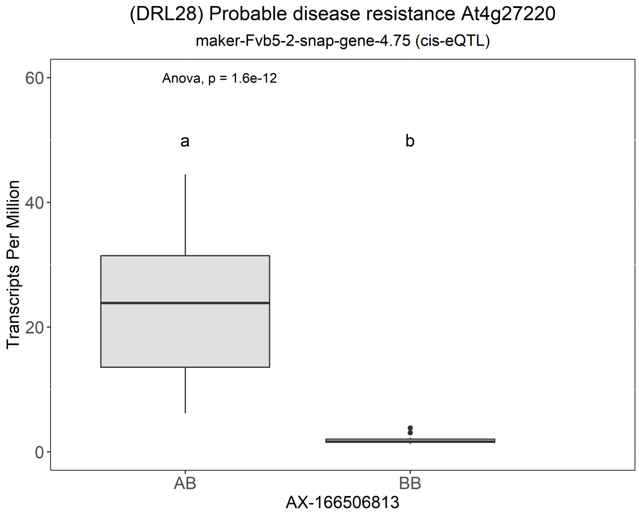
(DRL28) Probable disease resistance At4g27220
maker-Fvb5-2-snap-gene-4.75 (cis-eQTL)
Anova, p = 1.6e-12
a
b
Transcripts Per Million
0
20
40
60
AB
BB
AX-166506813

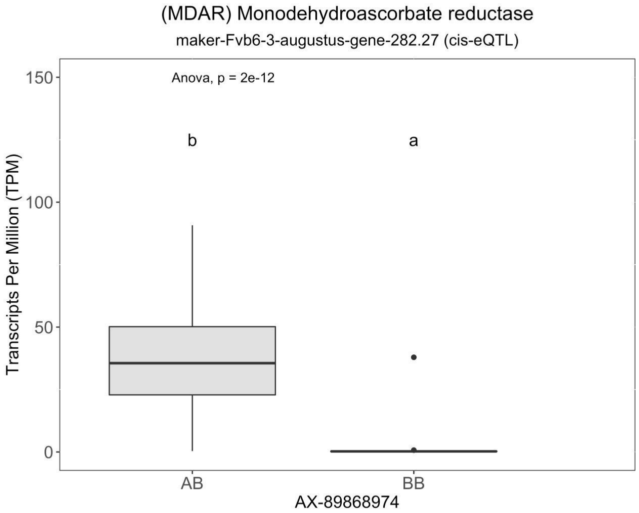
(MDAR) Monodehydroascorbate reductase
maker-Fvb6-3-augustus-gene-282.27 (cis-eQTL)
Anova, p = 2e-12
b
a
Transcripts Per Million (TPM)
0
50
100
150
AB
BB
AX-89868974

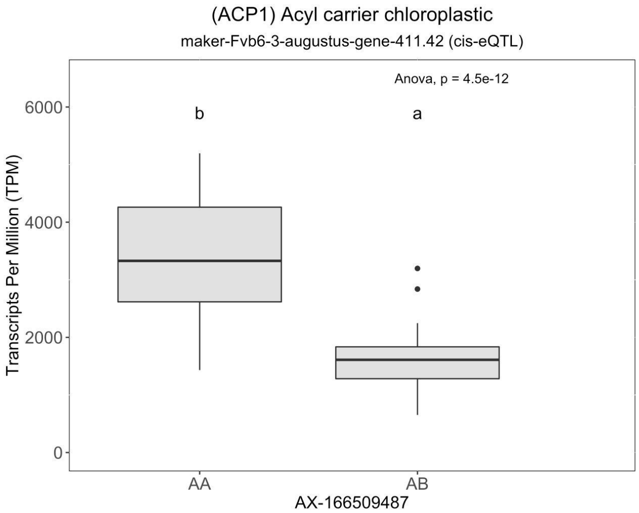
(ACP1) Acyl carrier chloroplastic
maker-Fvb6-3-augustus-gene-411.42 (cis-eQTL)
Anova, p = 4.5e-12
b
a
Transcripts Per Million (TPM)
0
2000
4000
6000
AA
AB
AX-166509487

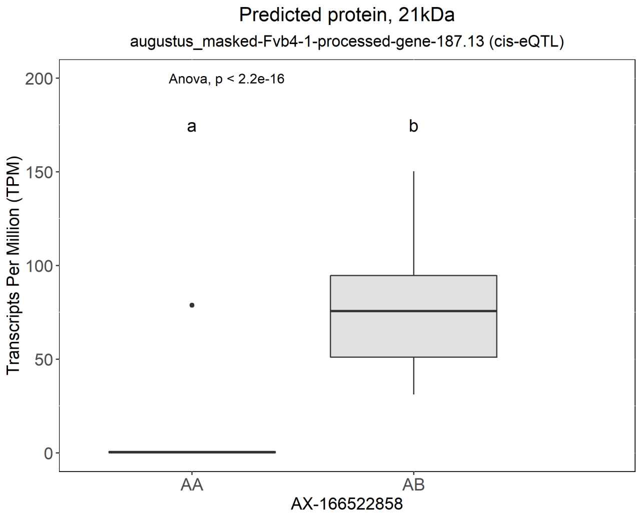
Predicted protein, 21kDa
augustus_masked-Fvb4-1-processed-gene-187.13 (cis-eQTL)
Anova, p < 2.2e-16
a
b
Transcripts Per Million (TPM)
0
50
100
150
200
AA
AB
AX-166522858

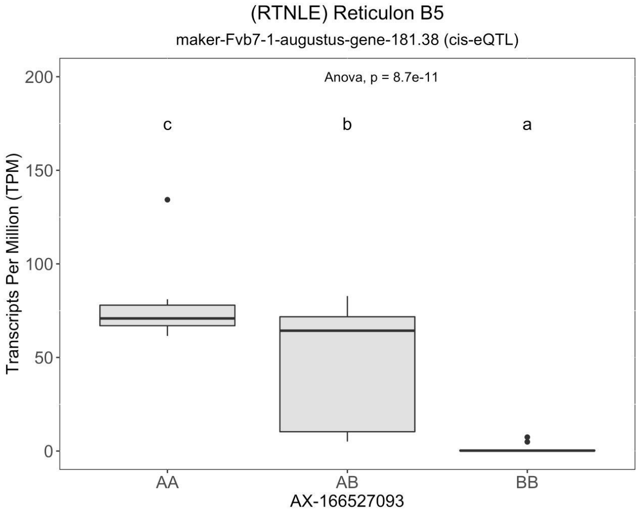
(RTNLE) Reticulon B5
maker-Fvb7-1-augustus-gene-181.38 (cis-eQTL)
Anova, p = 8.7e-11
c
b
a
Transcripts Per Million (TPM)
0
50
100
150
200
AA
AB
BB
AX-166527093

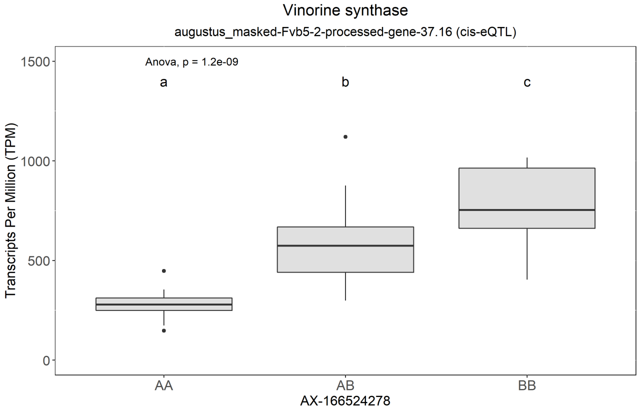
Vinorine synthase
augustus_masked-Fvb5-2-processed-gene-37.16 (cis-eQTL)
Anova, p = 1.2e-09
a
b
c
Transcripts Per Million (TPM)
0
500
1000
1500
AA
AB
BB
AX-166524278

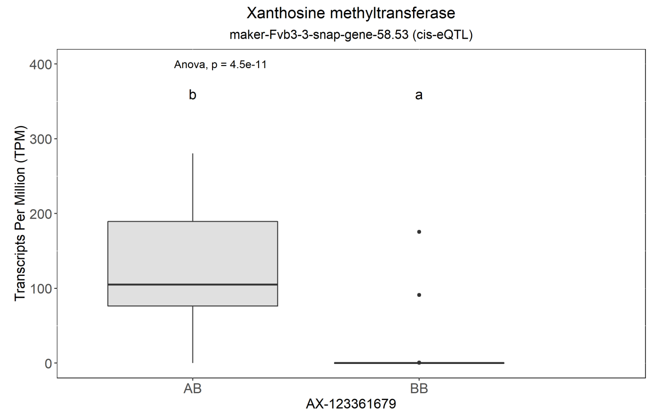
Xanthosine methyltransferase
maker-Fvb3-3-snap-gene-58.53 (cis-eQTL)
Anova, p = 4.5e-11
b
a
Transcripts Per Million (TPM)
400
300
200
100
0
AB
BB
AX-123361679

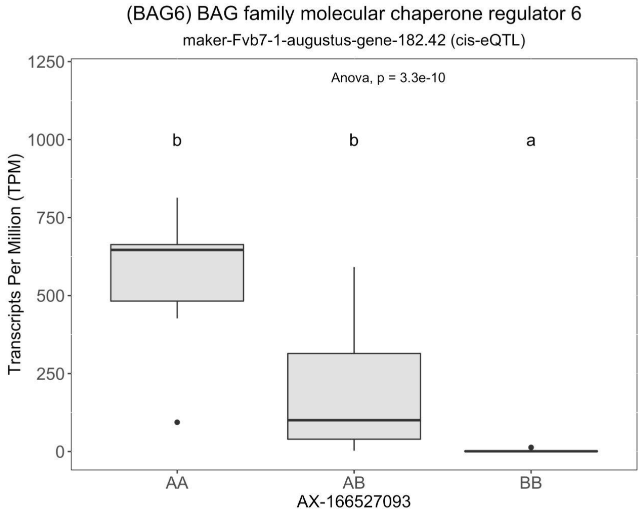
(BAG6) BAG family molecular chaperone regulator 6
maker-Fvb7-1-augustus-gene-182.42 (cis-eQTL)
Anova, p = 3.3e-10
b
b
a
Transcripts Per Million (TPM)
1250
1000
750
500
250
0
AA
AB
BB
AX-166527093

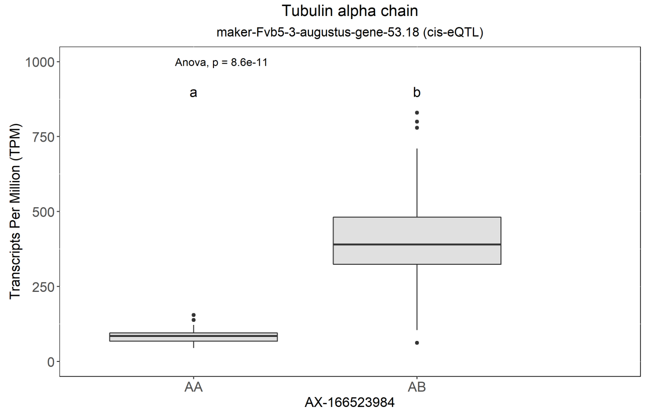
Tubulin alpha chain
maker-Fvb5-3-augustus-gene-53.18 (cis-eQTL)
Anova, p = 8.6e-11
a
b
Transcripts Per Million (TPM)
1000
750
500
250
0
AA
AB
AX-166523984

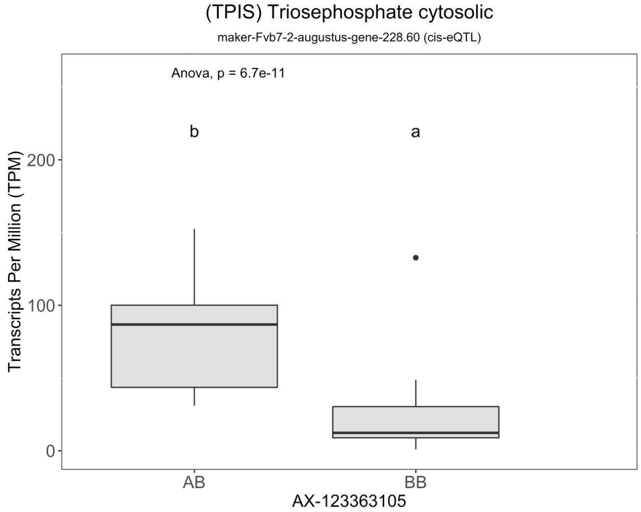
(TPIS) Triosephosphate cytosolic
maker-Fvb7-2-augustus-gene-228.60 (cis-eQTL)
Anova, p = 6.7e-11
b
a
Transcripts Per Million (TPM)
200
100
0
AB
BB
AX-123363105

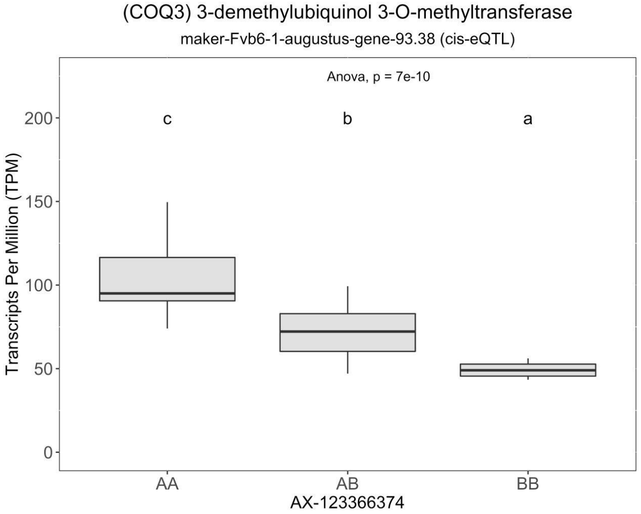
(COQ3) 3-demethylubiquinol 3-O-methyltransferase
maker-Fvb6-1-augustus-gene-93.38 (cis-eQTL)
Anova, p = 7e-10
c
b
a
Transcripts Per Million (TPM)
200
150
100
50
0
AA
AB
BB
AX-123366374

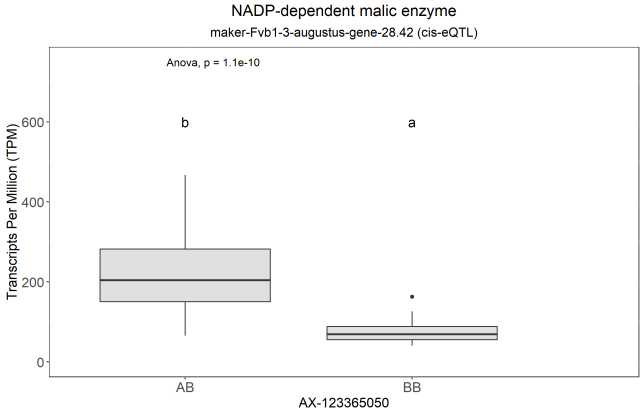
NADP-dependent malic enzyme
maker-Fvb1-3-augustus-gene-28.42 (cis-eQTL)
Anova, p = 1.1e-10
b
a
Transcripts Per Million (TPM)
600
400
200
0
AB
BB
AX-123365050

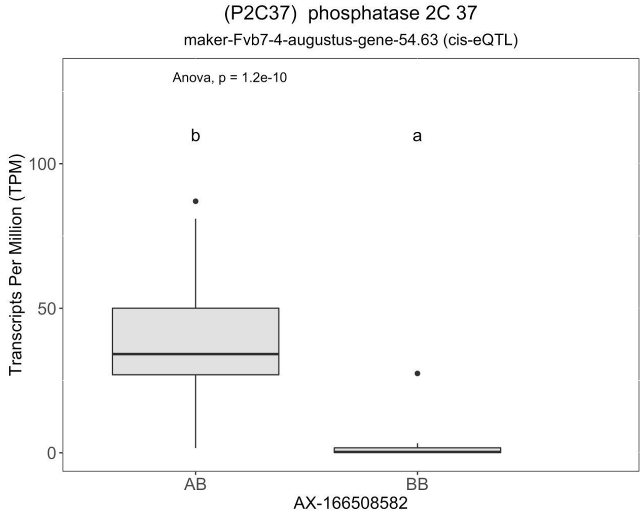
(P2C37) phosphatase 2C 37
maker-Fvb7-4-augustus-gene-54.63 (cis-eQTL)
Anova, p = 1.2e-10
b
a
Transcripts Per Million (TPM)
100
50
0
AB
BB
AX-166508582

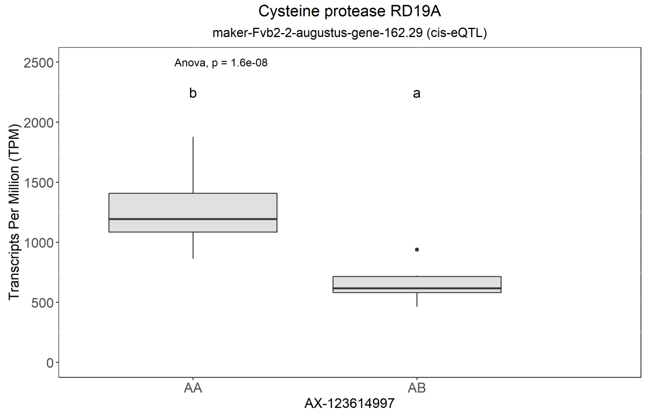
Cysteine protease RD19A
maker-Fvb2-2-augustus-gene-162.29 (cis-eQTL)
Anova, p = 1.6e-08
b
a
Transcripts Per Million (TPM)
2500
2000
1500
1000
500
0
AA
AB
AX-123614997

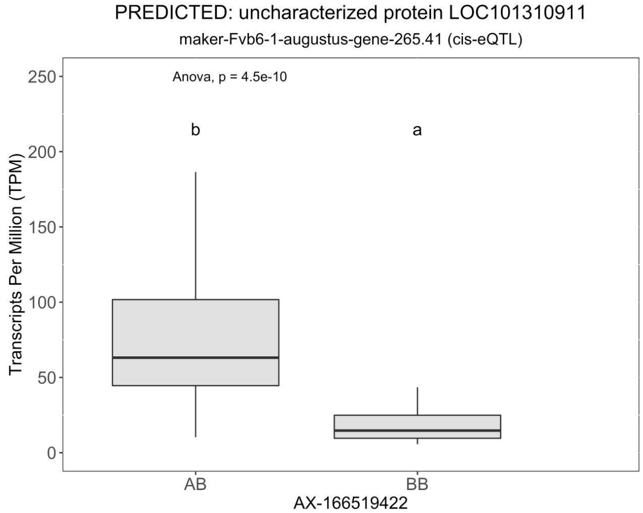
PREDICTED: uncharacterized protein LOC101310911
maker-Fvb6-1-augustus-gene-265.41 (cis-eQTL)
Anova, p = 4.5e-10
b
a
Transcripts Per Million (TPM)
250
200
150
100
50
0
AB
BB
AX-166519422

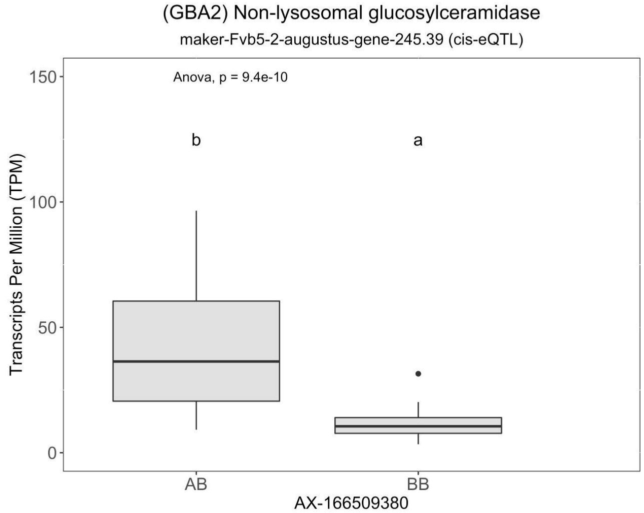
(GBA2) Non-lysosomal glucosylceramidase
maker-Fvb5-2-augustus-gene-245.39 (cis-eQTL)
Anova, p = 9.4e-10
b
a
Transcripts Per Million (TPM)
150
100
50
0
AB
BB
AX-166509380

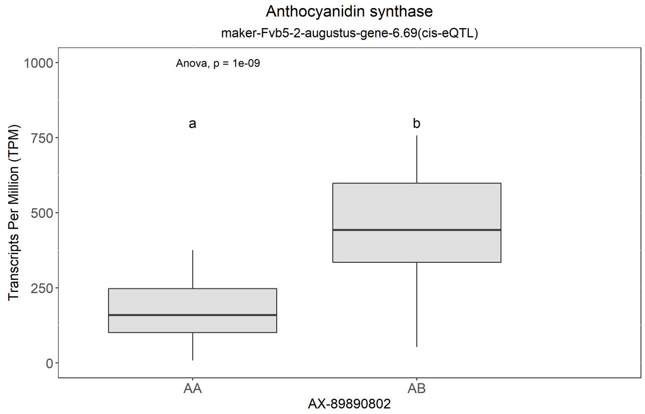
Anthocyanidin synthase
maker-Fvb5-2-augustus-gene-6.69(cis-eQTL)
Anova, p = 1e-09
a
b
Transcripts Per Million (TPM)
1000
750
500
250
0
AA
AB
AX-89890802

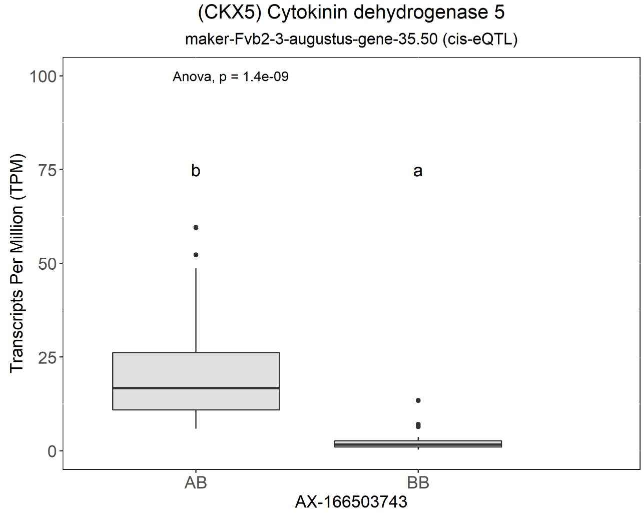
(CKX5) Cytokinin dehydrogenase 5
maker-Fvb2-3-augustus-gene-35.50 (cis-eQTL)
Anova, p = 1.4e-09
b
a
Transcripts Per Million (TPM)
100
75
50
25
0
AB
BB
AX-166503743

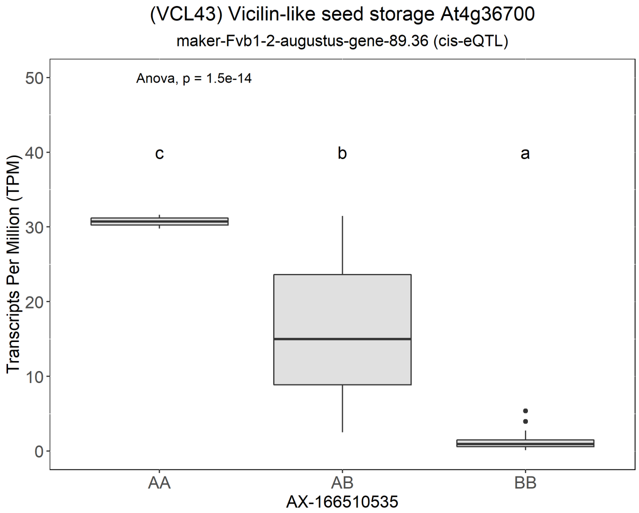
(VCL43) Vicilin-like seed storage At4g36700
maker-Fvb1-2-augustus-gene-89.36 (cis-eQTL)
Anova, p = 1.5e-14
c
b
a
Transcripts Per Million (TPM)
50
40
30
20
10
0
AA
AB
BB
AX-166510535

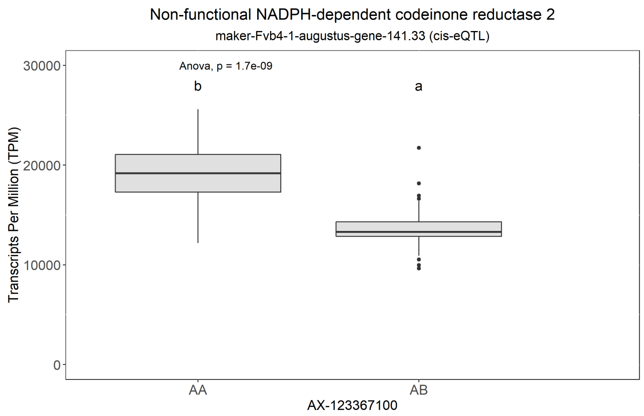
Non-functional NADPH-dependent codeinone reductase 2
maker-Fvb4-1-augustus-gene-141.33 (cis-eQTL)
Anova, p = 1.7e-09
b
a
Transcripts Per Million (TPM)
30000
20000
10000
0
AA
AB
AX-123367100

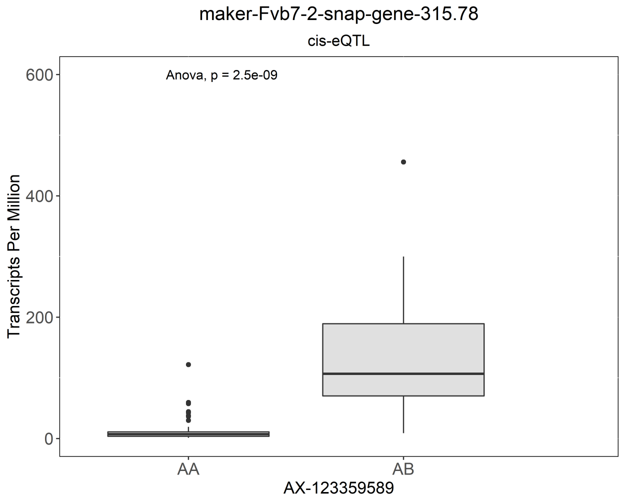
maker-Fvb7-2-snap-gene-315.78
cis-eQTL
Anova, p = 2.5e-09
Transcripts Per Million
600
400
200
0
AA
AB
AX-123359589

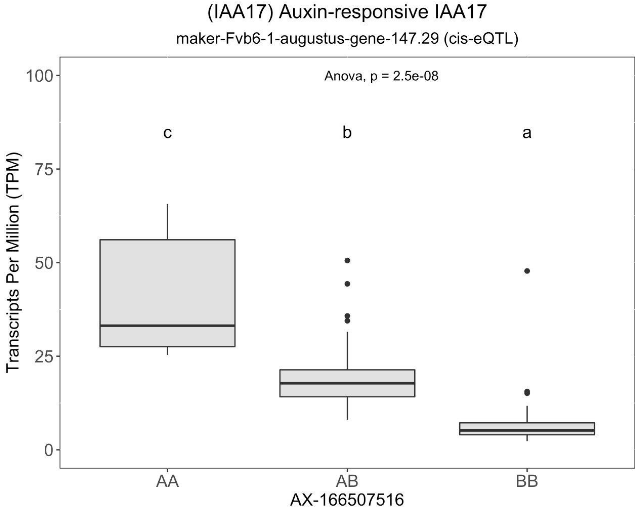
(IAA17) Auxin-responsive IAA17
maker-Fvb6-1-augustus-gene-147.29 (cis-eQTL)
Anova, p = 2.5e-08
c
b
a
Transcripts Per Million (TPM)
100
75
50
25
0
AA
AB
BB
AX-166507516

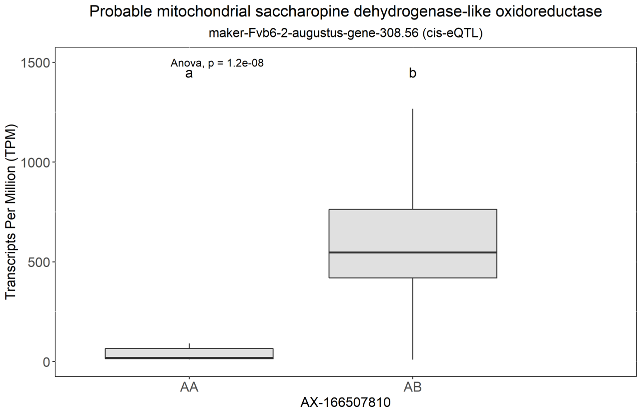
Probable mitochondrial saccharopine dehydrogenase-like oxidoreductase
maker-Fvb6-2-augustus-gene-308.56 (cis-eQTL)
Anova, p = 1.2e-08
a
b
Transcripts Per Million (TPM)
1500
1000
500
0
AA
AB
AX-166507810

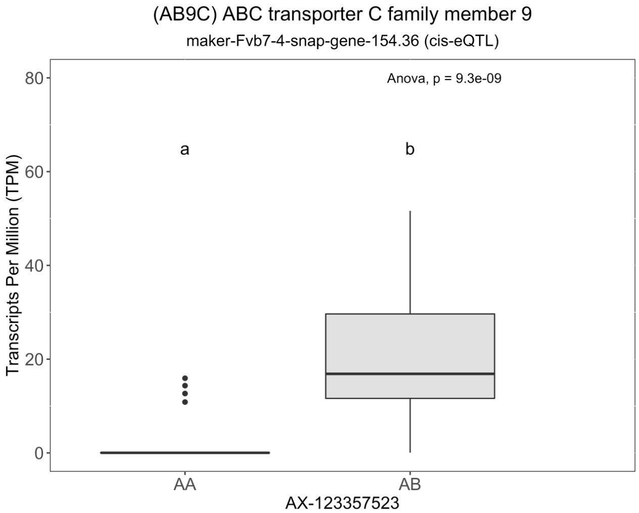
(AB9C) ABC transporter C family member 9
maker-Fvb7-4-snap-gene-154.36 (cis-eQTL)
Anova, p = 9.3e-09
a
b
Transcripts Per Million (TPM)
80
60
40
20
0
AA
AB
AX-123357523

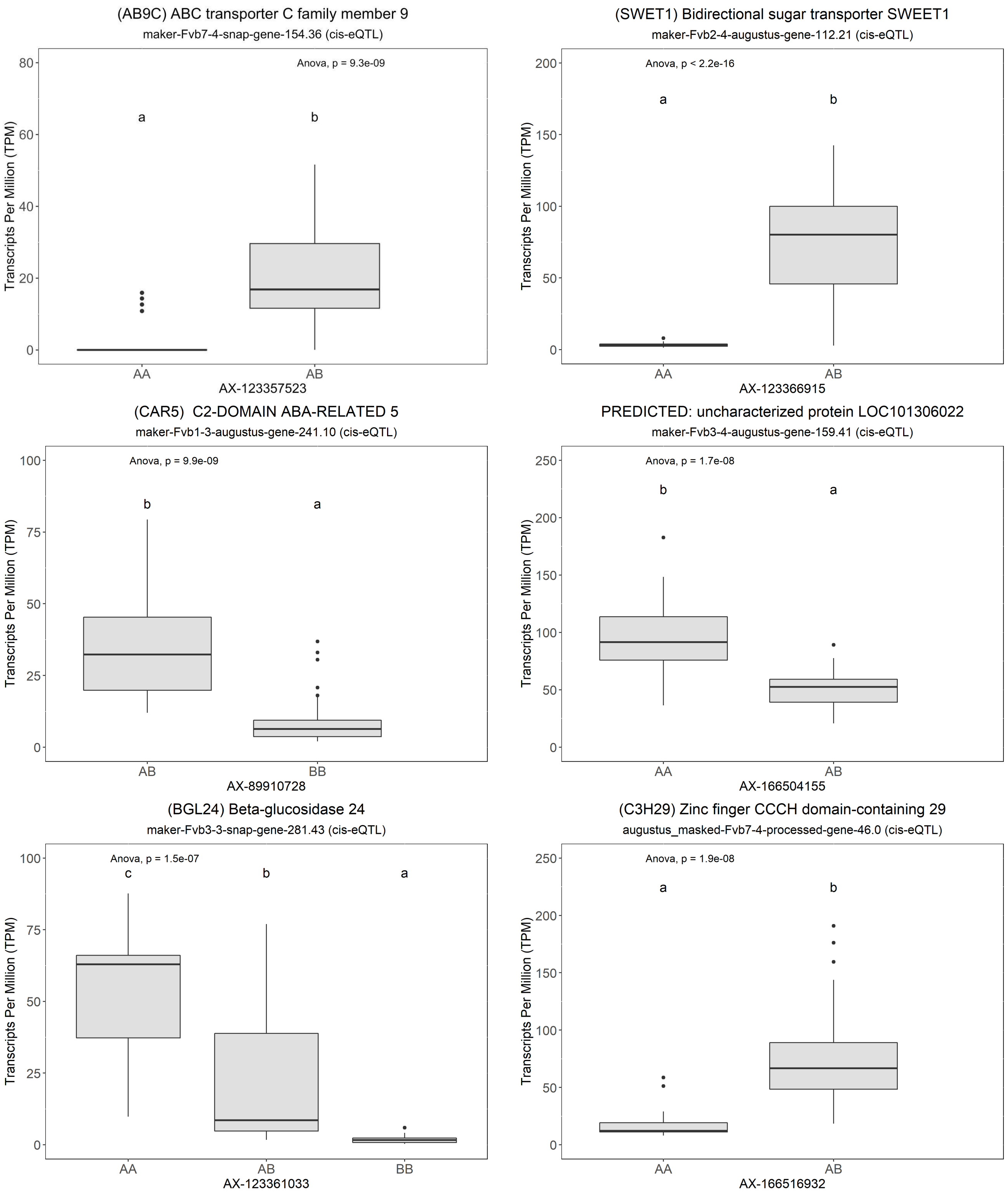
(AB9C) ABC transporter C family member 9
maker-Fvb7-4-snap-gene-154.36 (cis-eQTL)
Anova, p = 9.3e-09
Transcripts Per Million (TPM)
AX-123357523
(SWET1) Bidirectional sugar transporter SWEET1
maker-Fvb2-4-augustus-gene-112.21 (cis-eQTL)
Anova, p < 2.2e-16
AX-123366915
(CAR5) C2-DOMAIN ABA-RELATED 5
maker-Fvb1-3-augustus-gene-241.10 (cis-eQTL)
Anova, p = 9.9e-09
AX-89910728
PREDICTED: uncharacterized protein LOC101306022
maker-Fvb3-4-augustus-gene-159.41 (cis-eQTL)
Anova, p = 1.7e-08
AX-166504155
(BGL24) Beta-glucosidase 24
maker-Fvb3-3-snap-gene-281.43 (cis-eQTL)
Anova, p = 1.5e-07
AX-123361033
(C3H29) Zinc finger CCCH domain-containing 29
augustus_masked-Fvb7-4-processed-gene-46.0 (cis-eQTL)
Anova, p = 1.9e-08
AX-166516932

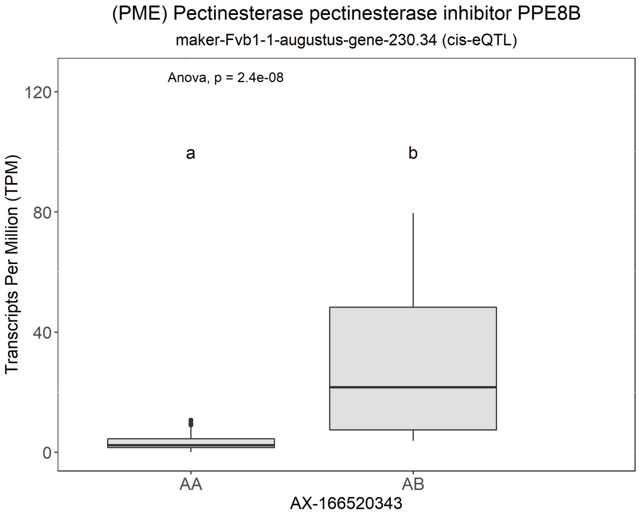
(PME) Pectinesterase pectinesterase inhibitor PPE8B
maker-Fvb1-1-augustus-gene-230.34 (cis-eQTL)
Anova, p = 2.4e-08
Transcripts Per Million (TPM)
a
b
AA
AB
AX-166520343

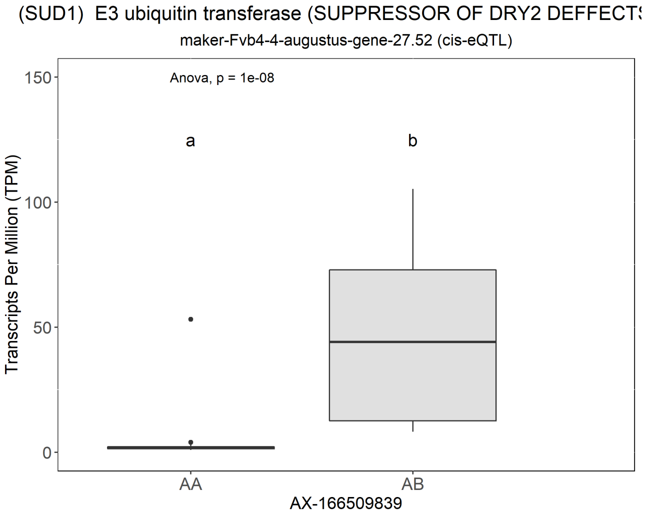
(SUD1) E3 ubiquitin transferase (SUPPRESSOR OF DRY2 DEFFECT
maker-Fvb4-4-augustus-gene-27.52 (cis-eQTL)
Anova, p = 1e-08
Transcripts Per Million (TPM)
a
b
AA
AB
AX-166509839

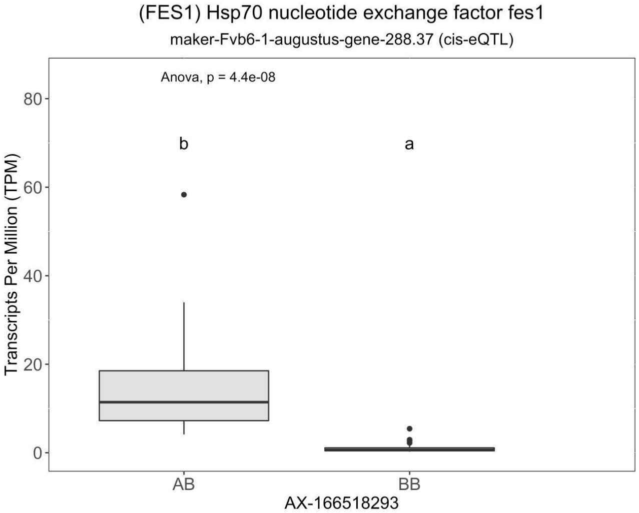
(FES1) Hsp70 nucleotide exchange factor fes1
maker-Fvb6-1-augustus-gene-288.37 (cis-eQTL)
Anova, p = 4.4e-08
Transcripts Per Million (TPM)
b
a
AB
BB
AX-166518293

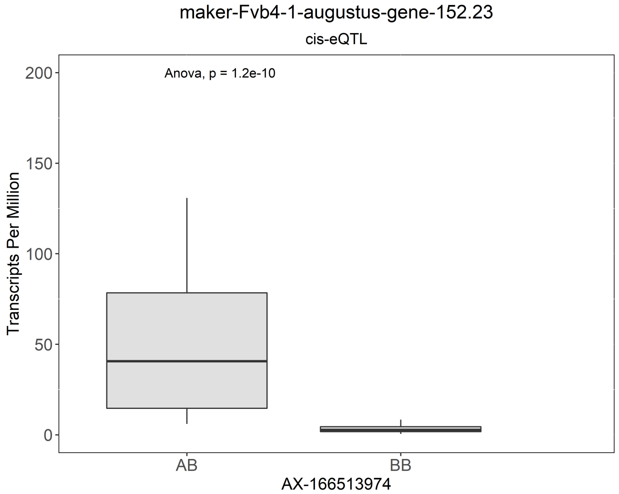
maker-Fvb4-1-augustus-gene-152.23
cis-eQTL
Anova, p = 1.2e-10
Transcripts Per Million
AB
BB
AX-166513974

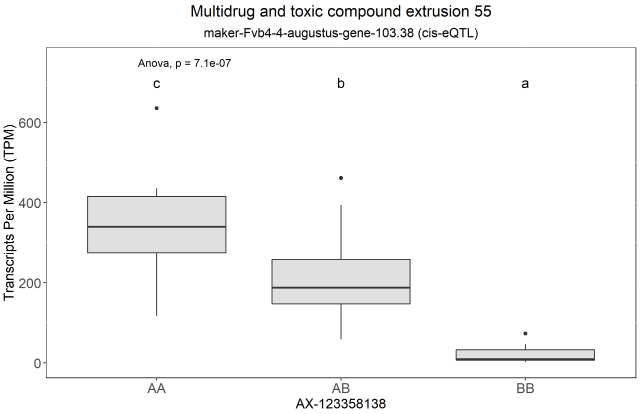
Multidrug and toxic compound extrusion 55
maker-Fvb4-4-augustus-gene-103.38 (cis-eQTL)
Anova, p = 7.1e-07
Transcripts Per Million (TPM)
c
b
a
AA
AB
BB
AX-123358138

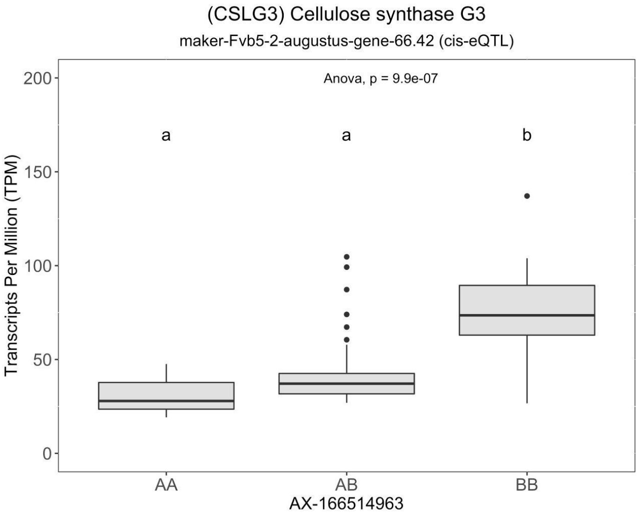
(CSLG3) Cellulose synthase G3
maker-Fvb5-2-augustus-gene-66.42 (cis-eQTL)
Anova, p = 9.9e-07
Transcripts Per Million (TPM)
a
a
b
AA
AB
BB
AX-166514963

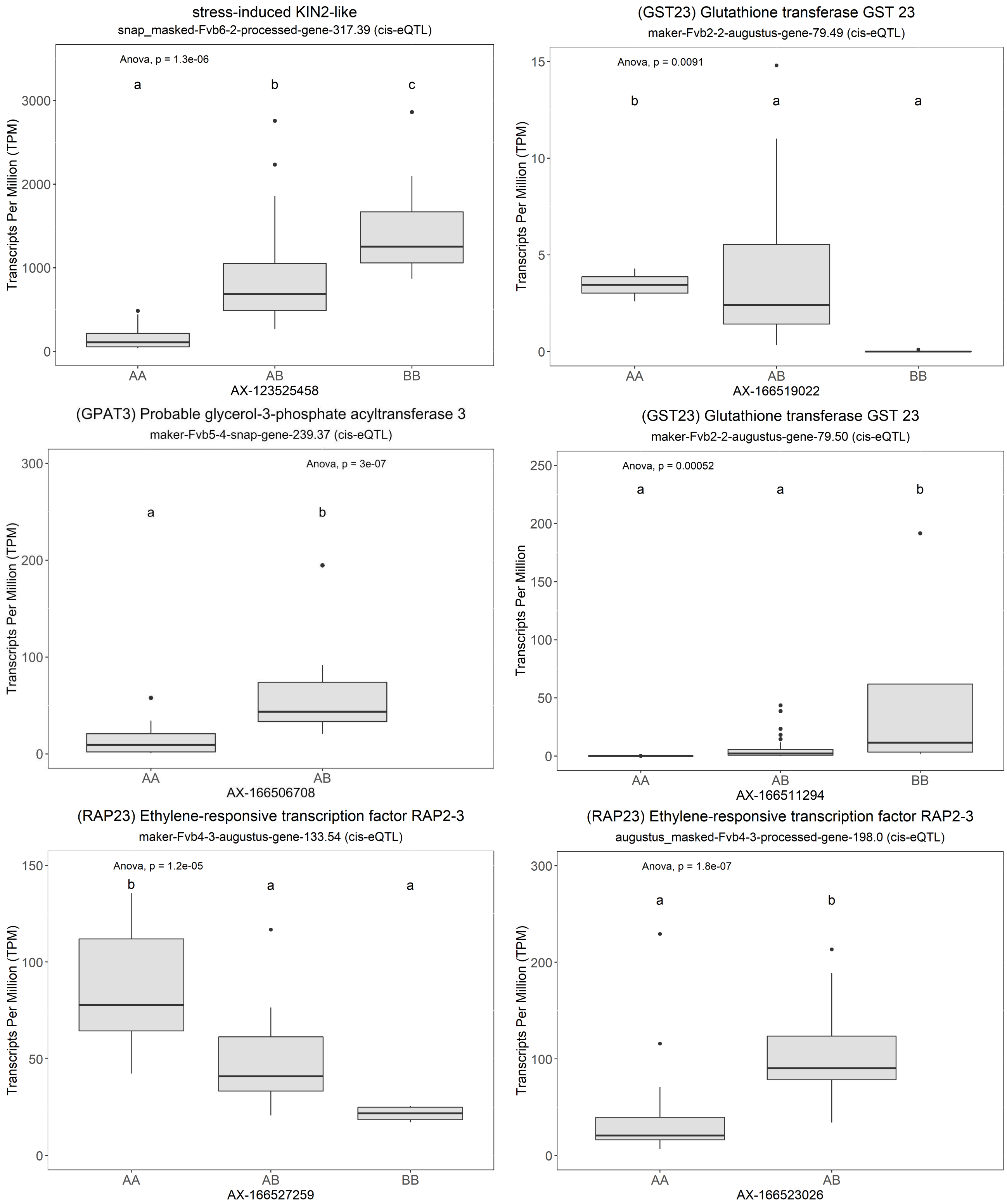
stress-induced KIN2-like
snap_masked-Fvb6-2-processed-gene-317.39 (cis-eQTL)
Anova, p = 1.3e-06
a
b
c
Transcripts Per Million (TPM)
0
1000
2000
3000
AA
AB
BB
AX-123525458
(GST23) Glutathione transferase GST 23
maker-Fvb2-2-augustus-gene-79.49 (cis-eQTL)
Anova, p = 0.0091
b
a
a
Transcripts Per Million (TPM)
0
5
10
15
AA
AB
BB
AX-166519022
(GPAT3) Probable glycerol-3-phosphate acyltransferase 3
maker-Fvb5-4-snap-gene-239.37 (cis-eQTL)
Anova, p = 3e-07
a
b
Transcripts Per Million (TPM)
0
100
200
300
AA
AB
AX-166506708
(GST23) Glutathione transferase GST 23
maker-Fvb2-2-augustus-gene-79.50 (cis-eQTL)
Anova, p = 0.00052
a
a
b
Transcripts Per Million
0
50
100
150
200
250
AA
AB
BB
AX-166511294
(RAP23) Ethylene-responsive transcription factor RAP2-3
maker-Fvb4-3-augustus-gene-133.54 (cis-eQTL)
Anova, p = 1.2e-05
b
a
a
Transcripts Per Million (TPM)
0
50
100
150
AA
AB
BB
AX-166527259
(RAP23) Ethylene-responsive transcription factor RAP2-3
augustus_masked-Fvb4-3-processed-gene-198.0 (cis-eQTL)
Anova, p = 1.8e-07
a
b
Transcripts Per Million (TPM)
0
100
200
300
AA
AB
AX-166523026

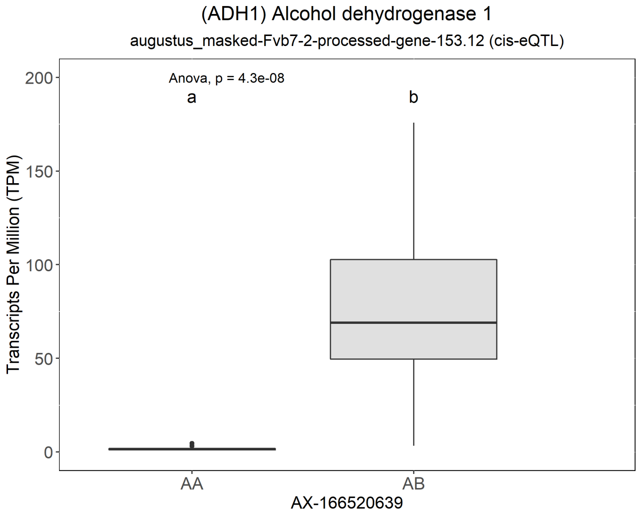
(ADH1) Alcohol dehydrogenase 1
augustus_masked-Fvb7-2-processed-gene-153.12 (cis-eQTL)
Anova, p = 4.3e-08
a
b
Transcripts Per Million (TPM)
0
50
100
150
200
AA
AB
AX-166520639

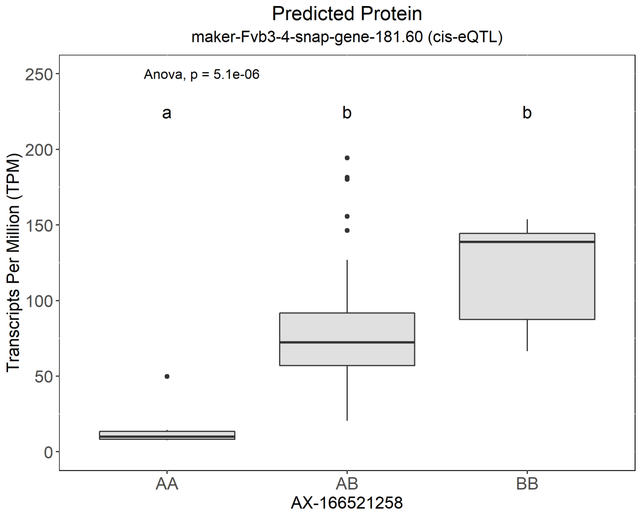
Predicted Protein
maker-Fvb3-4-snap-gene-181.60 (cis-eQTL)
Anova, p = 5.1e-06
a
b
b
Transcripts Per Million (TPM)
0
50
100
150
200
250
AA
AB
BB
AX-166521258

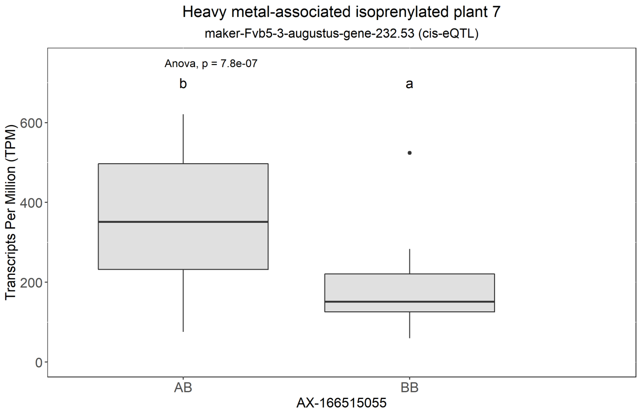
Heavy metal-associated isoprenylated plant 7
maker-Fvb5-3-augustus-gene-232.53 (cis-eQTL)
Anova, p = 7.8e-07
b
a
Transcripts Per Million (TPM)
0
200
400
600
AB
BB
AX-166515055

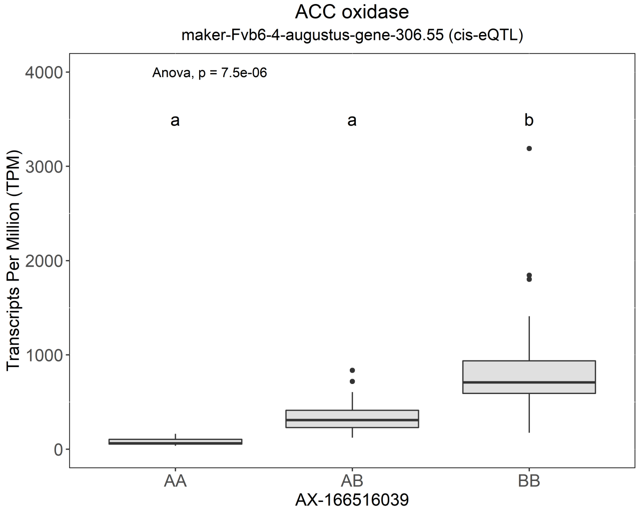
ACC oxidase
maker-Fvb6-4-augustus-gene-306.55 (cis-eQTL)
Anova, p = 7.5e-06
a
a
b
Transcripts Per Million (TPM)
0
1000
2000
3000
4000
AA
AB
BB
AX-166516039

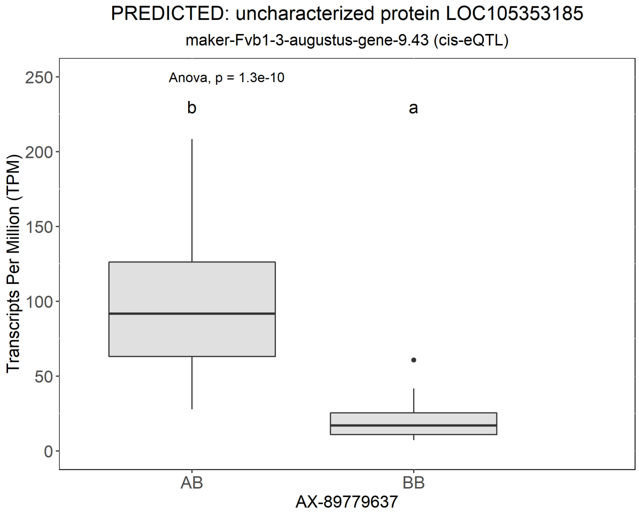
PREDICTED: uncharacterized protein LOC105353185
maker-Fvb1-3-augustus-gene-9.43 (cis-eQTL)
Anova, p = 1.3e-10
b
a
Transcripts Per Million (TPM)
0
50
100
150
200
250
AB
BB
AX-89779637

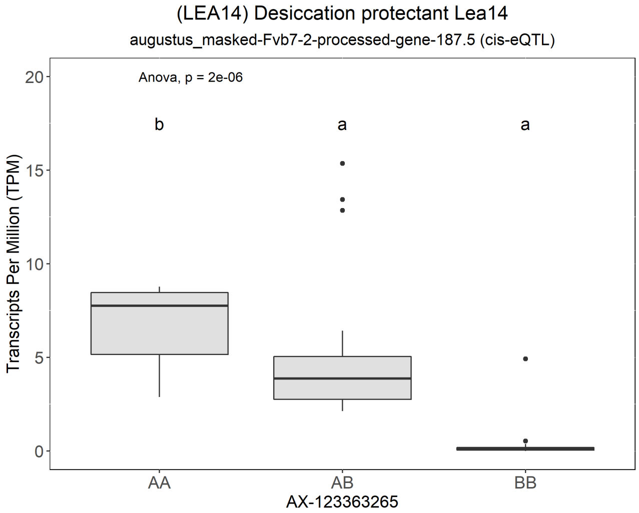
(LEA14) Desiccation protectant Lea14
augustus_masked-Fvb7-2-processed-gene-187.5 (cis-eQTL)
Anova, p = 2e-06
b
a
a
Transcripts Per Million (TPM)
0
5
10
15
20
AA
AB
BB
AX-123363265

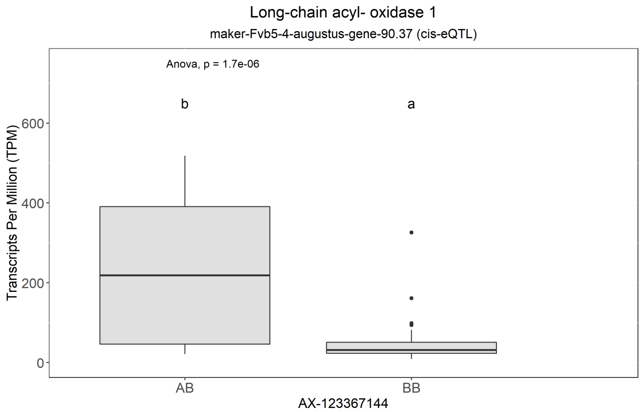
Long-chain acyl- oxidase 1
maker-Fvb5-4-augustus-gene-90.37 (cis-eQTL)
Anova, p = 1.7e-06
b
a
Transcripts Per Million (TPM)
AB
BB
AX-123367144

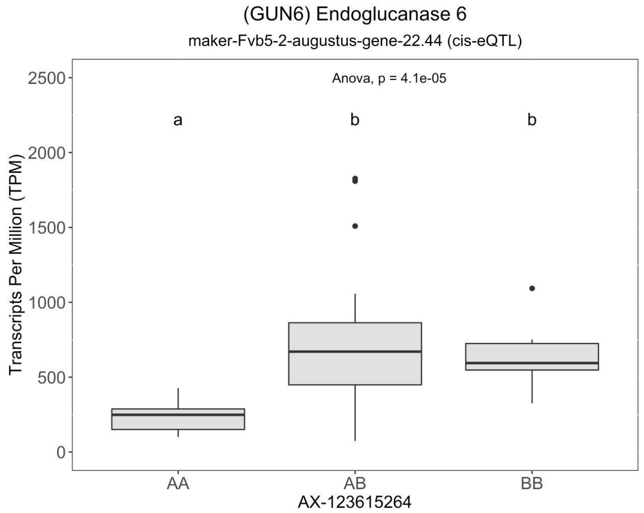
(GUN6) Endoglucanase 6
maker-Fvb5-2-augustus-gene-22.44 (cis-eQTL)
Anova, p = 4.1e-05
a
b
b
Transcripts Per Million (TPM)
AA
AB
BB
AX-123615264

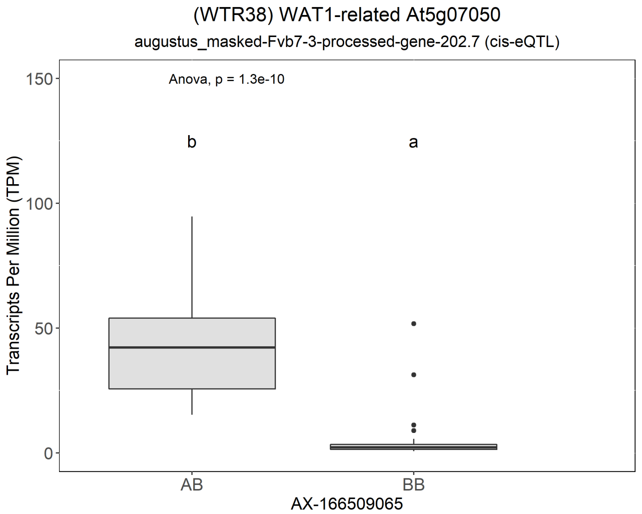
(WTR38) WAT1-related At5g07050
augustus_masked-Fvb7-3-processed-gene-202.7 (cis-eQTL)
Anova, p = 1.3e-10
b
a
Transcripts Per Million (TPM)
AB
BB
AX-166509065

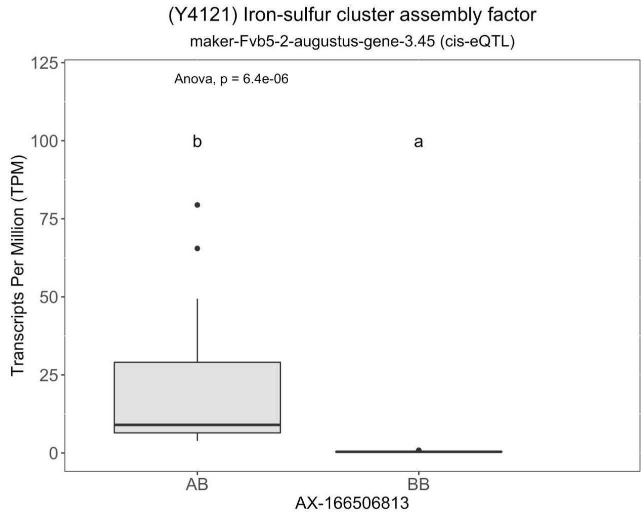
(Y4121) Iron-sulfur cluster assembly factor
maker-Fvb5-2-augustus-gene-3.45 (cis-eQTL)
Anova, p = 6.4e-06
b
a
Transcripts Per Million (TPM)
AB
BB
AX-166506813

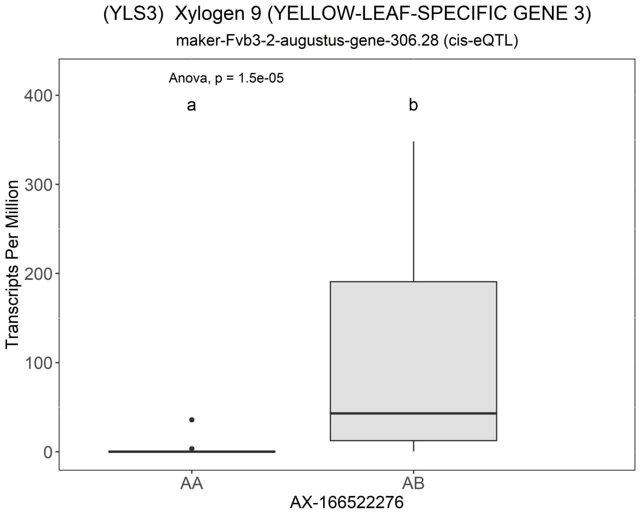
(YLS3) Xylogen 9 (YELLOW-LEAF-SPECIFIC GENE 3)
maker-Fvb3-2-augustus-gene-306.28 (cis-eQTL)
Anova, p = 1.5e-05
a
b
Transcripts Per Million
AA
AB
AX-166522276

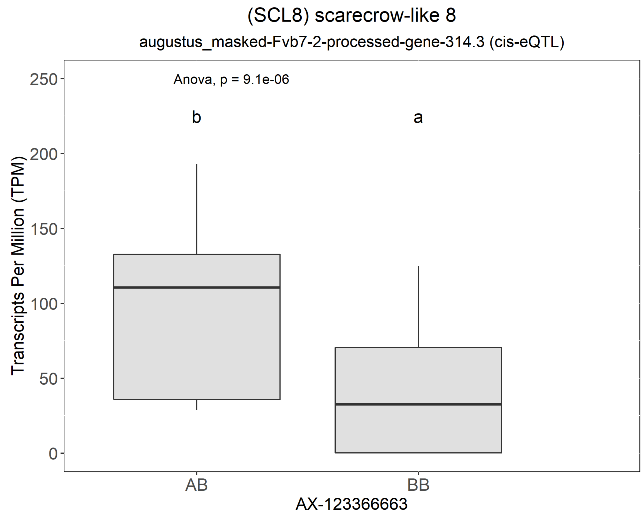
(SCL8) scarecrow-like 8
augustus_masked-Fvb7-2-processed-gene-314.3 (cis-eQTL)
Anova, p = 9.1e-06
b
a
Transcripts Per Million (TPM)
AB
BB
AX-123366663

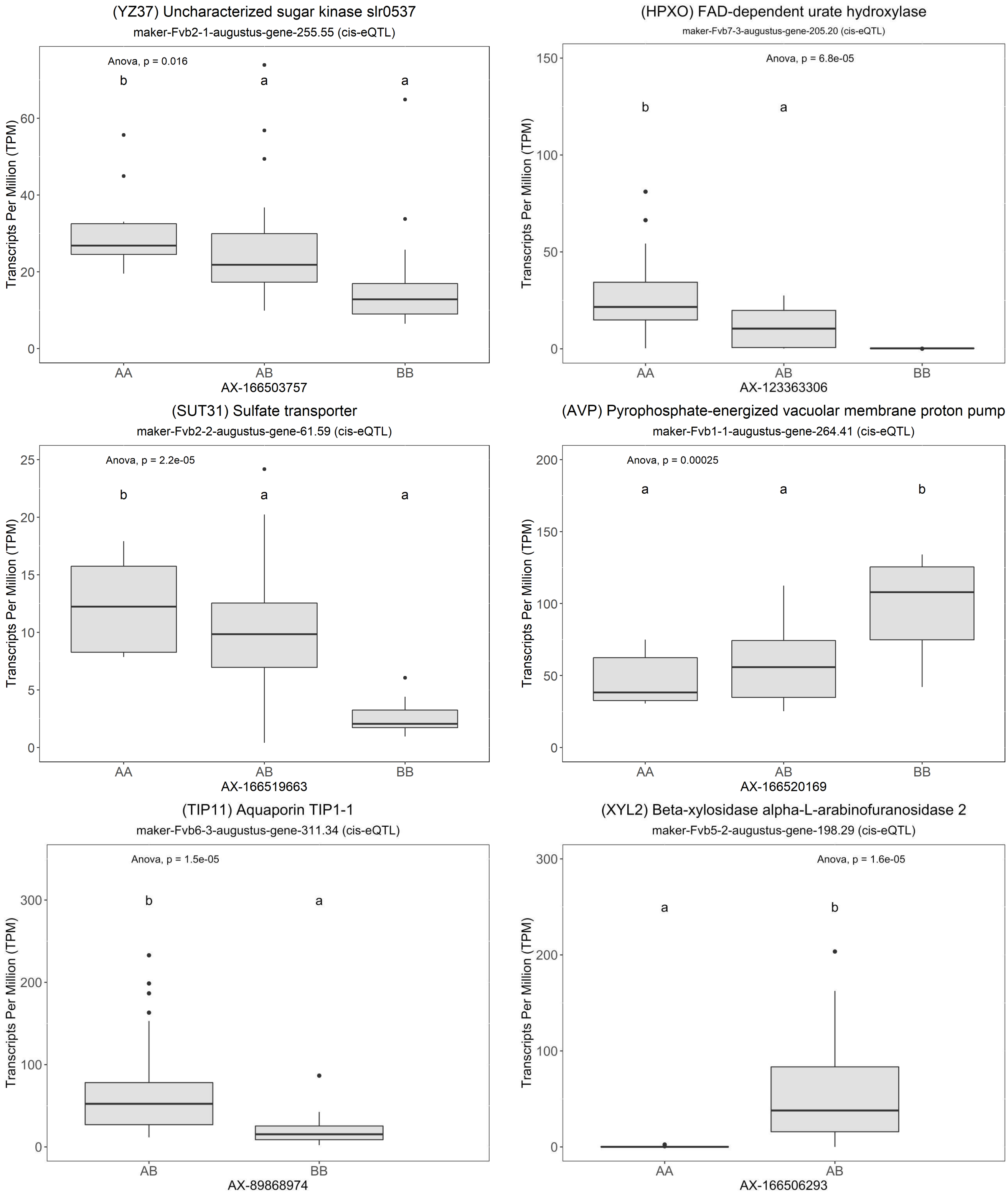
(YZ37) Uncharacterized sugar kinase slr0537
maker-Fvb2-1-augustus-gene-255.55 (cis-eQTL)
Anova, p = 0.016
b
a
a
Transcripts Per Million (TPM)
0
20
40
60
AA
AB
BB
AX-166503757
(HPXO) FAD-dependent urate hydroxylase
maker-Fvb7-3-augustus-gene-205.20 (cis-eQTL)
Anova, p = 6.8e-05
b
a
Transcripts Per Million (TPM)
0
50
100
150
AA
AB
BB
AX-123363306
(SUT31) Sulfate transporter
maker-Fvb2-2-augustus-gene-61.59 (cis-eQTL)
Anova, p = 2.2e-05
b
a
a
Transcripts Per Million (TPM)
0
5
10
15
20
25
AA
AB
BB
AX-166519663
(AVP) Pyrophosphate-energized vacuolar membrane proton pump
maker-Fvb1-1-augustus-gene-264.41 (cis-eQTL)
Anova, p = 0.00025
a
a
b
Transcripts Per Million (TPM)
0
50
100
150
200
AA
AB
BB
AX-166520169
(TIP11) Aquaporin TIP1-1
maker-Fvb6-3-augustus-gene-311.34 (cis-eQTL)
Anova, p = 1.5e-05
b
a
Transcripts Per Million (TPM)
0
100
200
300
AB
BB
AX-89868974
(XYL2) Beta-xylosidase alpha-L-arabinofuranosidase 2
maker-Fvb5-2-augustus-gene-198.29 (cis-eQTL)
Anova, p = 1.6e-05
a
b
Transcripts Per Million (TPM)
0
100
200
300
AA
AB
AX-166506293

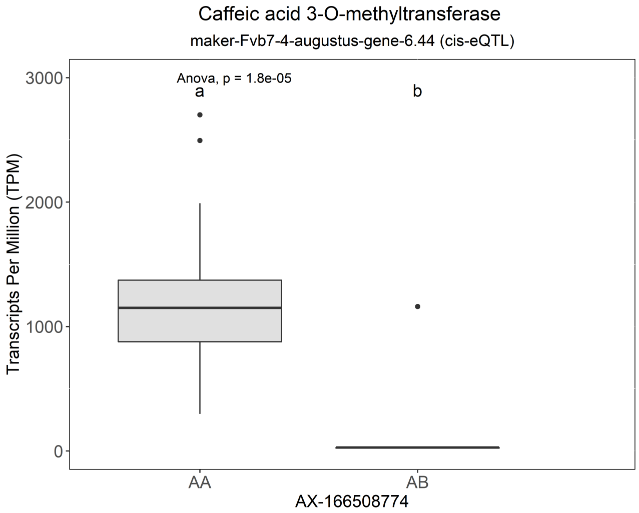
Caffeic acid 3-O-methyltransferase
maker-Fvb7-4-augustus-gene-6.44 (cis-eQTL)
Anova, p = 1.8e-05
a
b
Transcripts Per Million (TPM)
0
1000
2000
3000
AA
AB
AX-166508774

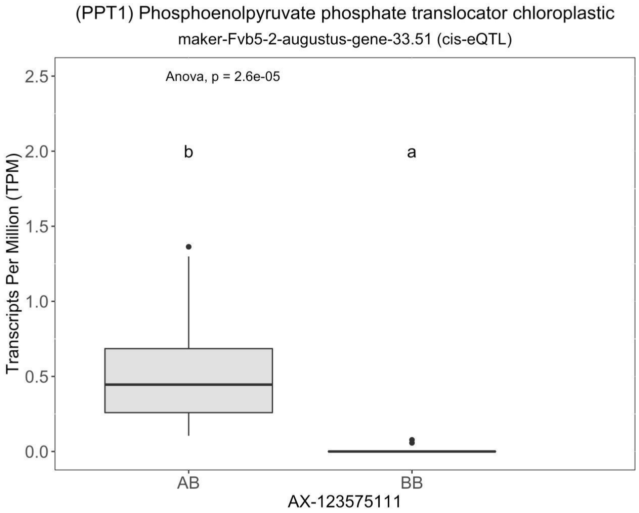
(PPT1) Phosphoenolpyruvate phosphate translocator chloroplastic
maker-Fvb5-2-augustus-gene-33.51 (cis-eQTL)
Anova, p = 2.6e-05
b
a
Transcripts Per Million (TPM)
0.0
0.5
1.0
1.5
2.0
2.5
AB
BB
AX-123575111

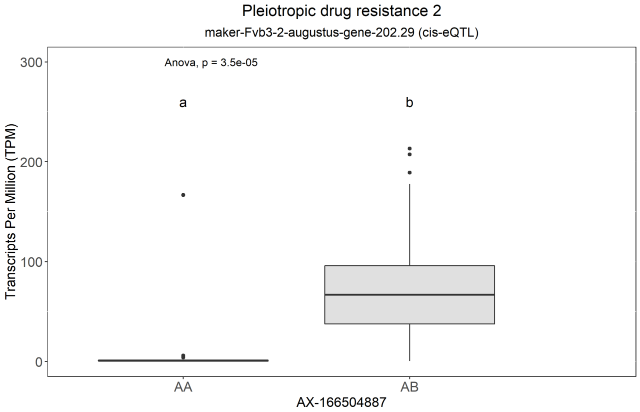
Pleiotropic drug resistance 2
maker-Fvb3-2-augustus-gene-202.29 (cis-eQTL)
Anova, p = 3.5e-05
a
b
Transcripts Per Million (TPM)
0
100
200
300
AA
AB
AX-166504887

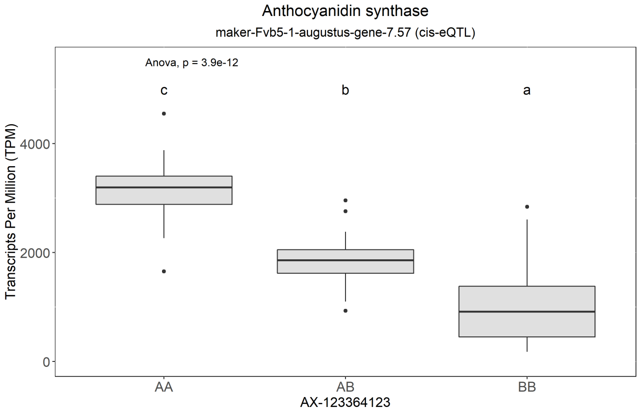
Anthocyanidin synthase
maker-Fvb5-1-augustus-gene-7.57 (cis-eQTL)
Anova, p = 3.9e-12
c
b
a
Transcripts Per Million (TPM)
0
2000
4000
AA
AB
BB
AX-123364123

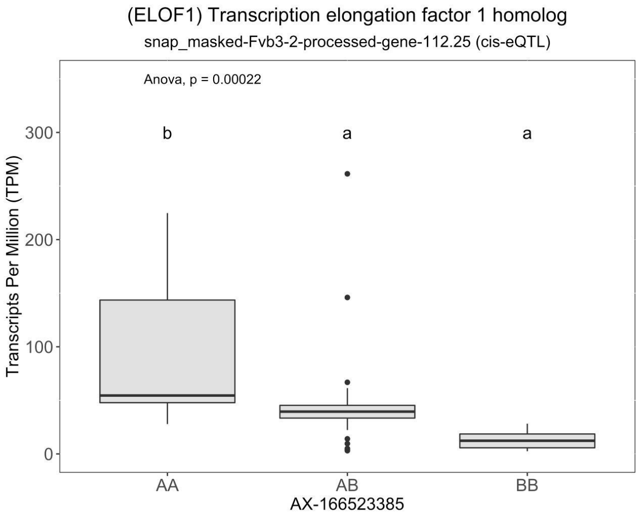
(ELOF1) Transcription elongation factor 1 homolog
snap_masked-Fvb3-2-processed-gene-112.25 (cis-eQTL)
Anova, p = 0.00022
b
a
a
Transcripts Per Million (TPM)
0
100
200
300
AA
AB
BB
AX-166523385

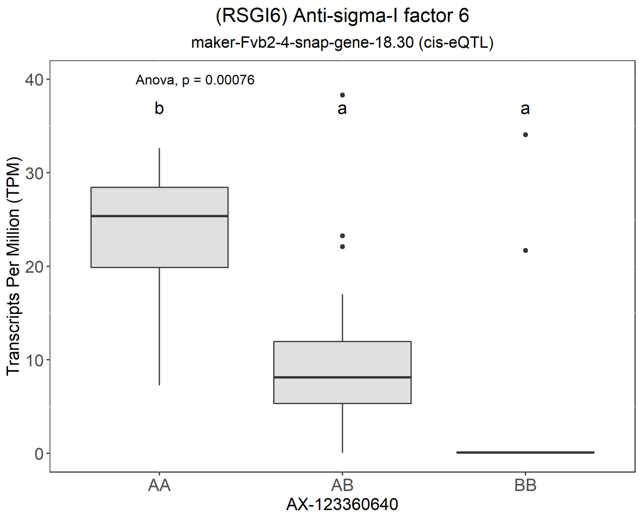
(RSGI6) Anti-sigma-I factor 6
maker-Fvb2-4-snap-gene-18.30 (cis-eQTL)
Anova, p = 0.00076
b
a
a
Transcripts Per Million (TPM)
0
10
20
30
40
AA
AB
BB
AX-123360640

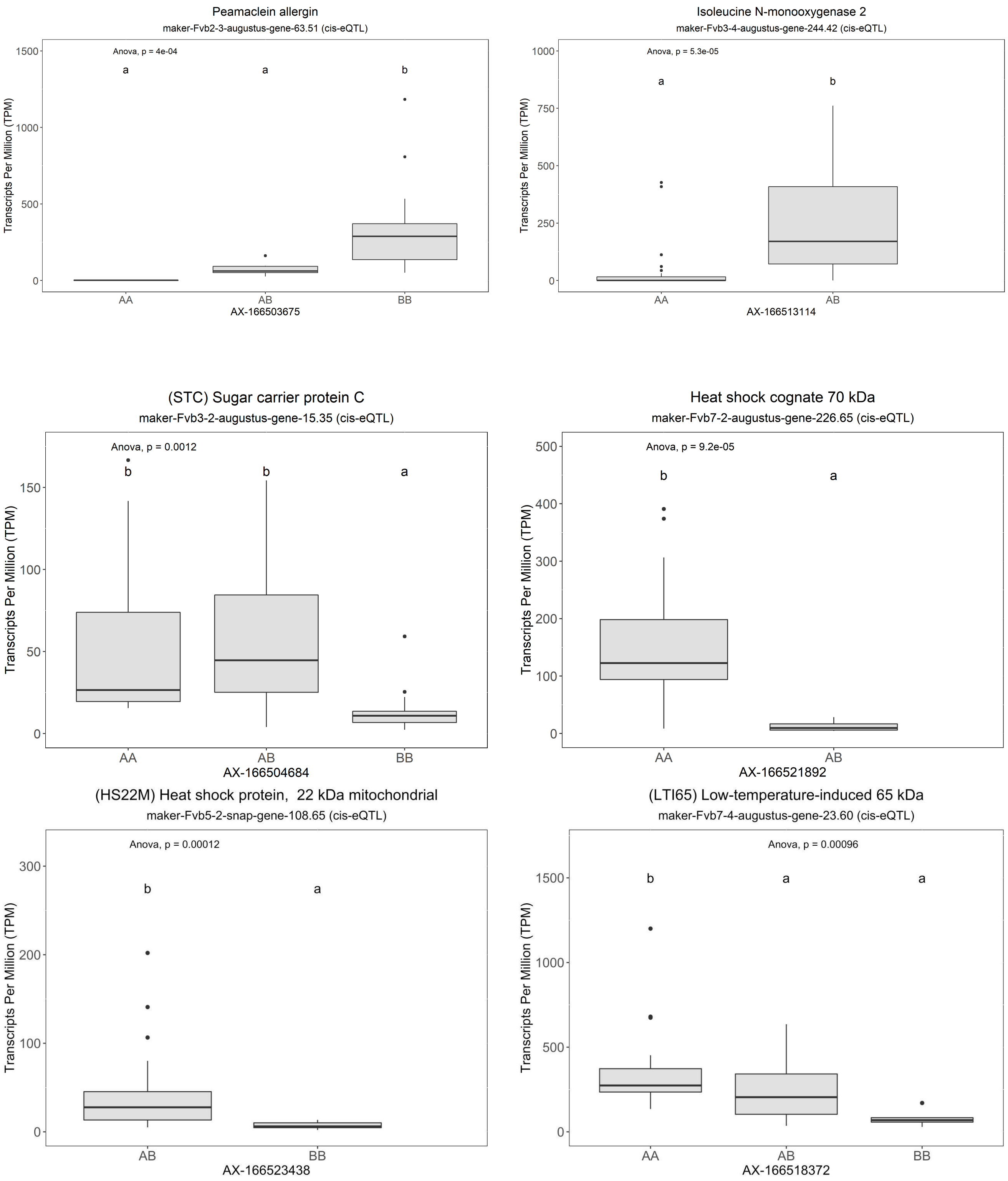
Peamaclein allergin
maker-Fvb2-3-augustus-gene-63.51 (cis-eQTL)
Anova, p = 4e-04
a
a
b
Transcripts Per Million (TPM)
0
500
1000
1500
AA
AB
BB
AX-166503675
Isoleucine N-monooxygenase 2
maker-Fvb3-4-augustus-gene-244.42 (cis-eQTL)
Anova, p = 5.3e-05
a
b
Transcripts Per Million (TPM)
0
250
500
750
1000
AA
AB
AX-166513114
(STC) Sugar carrier protein C
maker-Fvb3-2-augustus-gene-15.35 (cis-eQTL)
Anova, p = 0.0012
b
b
a
Transcripts Per Million (TPM)
0
50
100
150
AA
AB
BB
AX-166504684
Heat shock cognate 70 kDa
maker-Fvb7-2-augustus-gene-226.65 (cis-eQTL)
Anova, p = 9.2e-05
b
a
Transcripts Per Million (TPM)
0
100
200
300
400
500
AA
AB
AX-166521892
(HS22M) Heat shock protein, 22 kDa mitochondrial
maker-Fvb5-2-snap-gene-108.65 (cis-eQTL)
Anova, p = 0.00012
b
a
Transcripts Per Million (TPM)
0
100
200
300
AB
BB
AX-166523438
(LTI65) Low-temperature-induced 65 kDa
maker-Fvb7-4-augustus-gene-23.60 (cis-eQTL)
Anova, p = 0.00096
b
a
a
Transcripts Per Million (TPM)
0
500
1000
1500
AA
AB
BB
AX-166518372

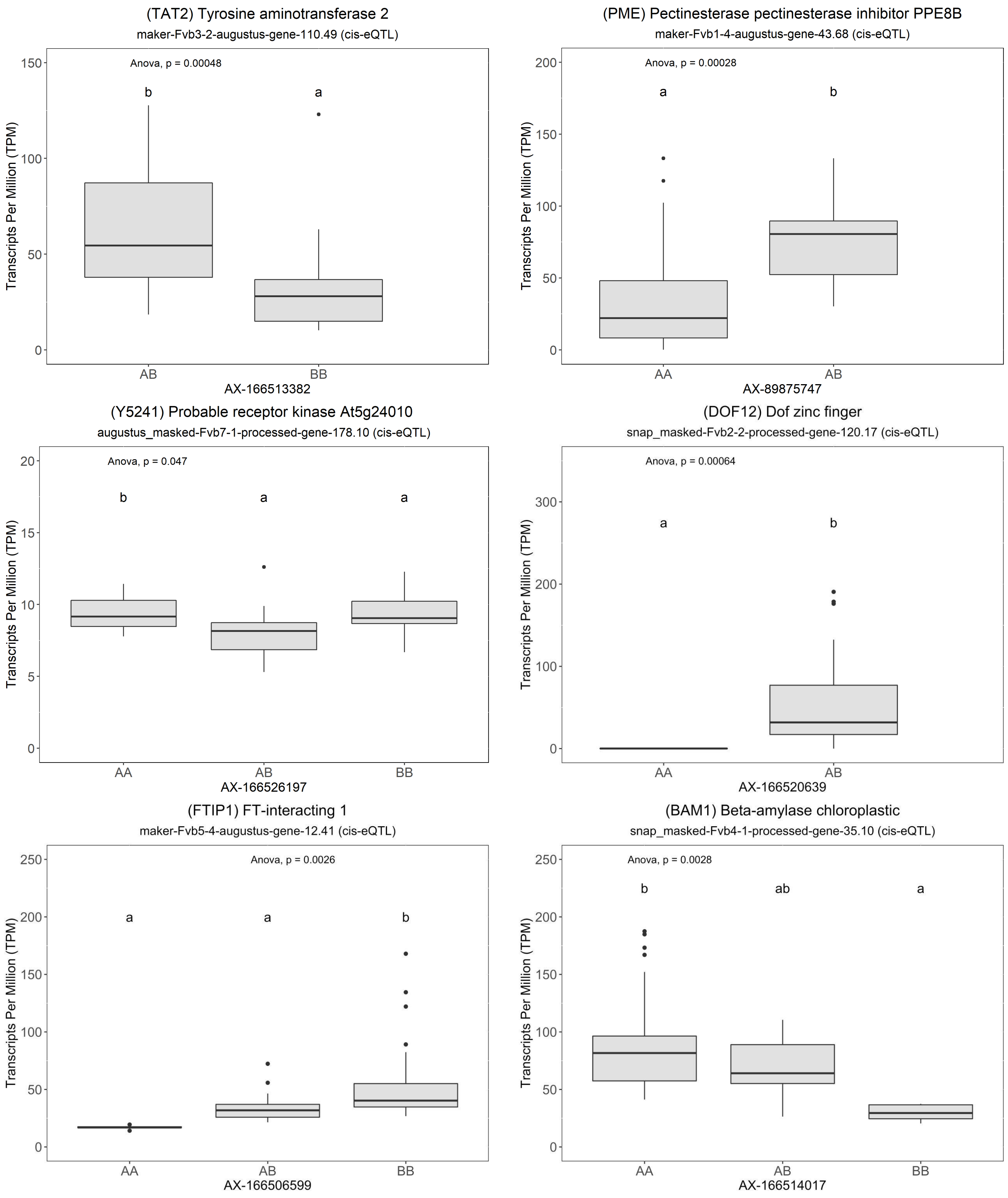
(TAT2) Tyrosine aminotransferase 2
maker-Fvb3-2-augustus-gene-110.49 (cis-eQTL)
Anova, p = 0.00048
b
a
Transcripts Per Million (TPM)
150
100
50
0
AB
BB
AX-166513382
(PME) Pectinesterase pectinesterase inhibitor PPE8B
maker-Fvb1-4-augustus-gene-43.68 (cis-eQTL)
Anova, p = 0.00028
a
b
Transcripts Per Million (TPM)
200
150
100
50
0
AA
AB
AX-89875747
(Y5241) Probable receptor kinase At5g24010
augustus_masked-Fvb7-1-processed-gene-178.10 (cis-eQTL)
Anova, p = 0.047
b
a
a
Transcripts Per Million (TPM)
20
15
10
5
0
AA
AB
BB
AX-166526197
(DOF12) Dof zinc finger
snap_masked-Fvb2-2-processed-gene-120.17 (cis-eQTL)
Anova, p = 0.00064
a
b
Transcripts Per Million (TPM)
300
200
100
0
AA
AB
AX-166520639
(FTIP1) FT-interacting 1
maker-Fvb5-4-augustus-gene-12.41 (cis-eQTL)
Anova, p = 0.0026
a
a
b
Transcripts Per Million (TPM)
250
200
150
100
50
0
AA
AB
BB
AX-166506599
(BAM1) Beta-amylase chloroplastic
snap_masked-Fvb4-1-processed-gene-35.10 (cis-eQTL)
Anova, p = 0.0028
b
ab
a
Transcripts Per Million (TPM)
250
200
150
100
50
0
AA
AB
BB
AX-166514017

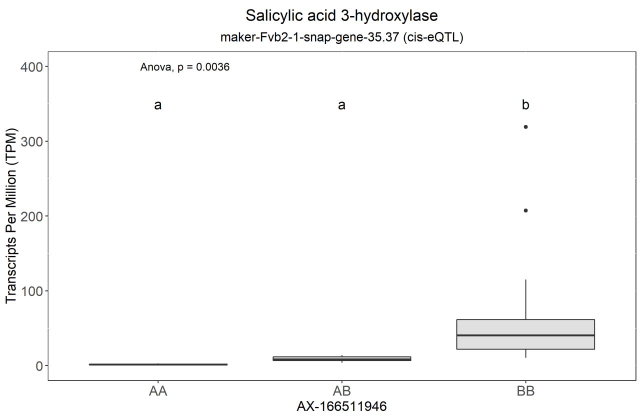
Salicylic acid 3-hydroxylase
maker-Fvb2-1-snap-gene-35.37 (cis-eQTL)
Anova, p = 0.0036
a
a
b
Transcripts Per Million (TPM)
0
100
200
300
400
AA
AB
BB
AX-166511946

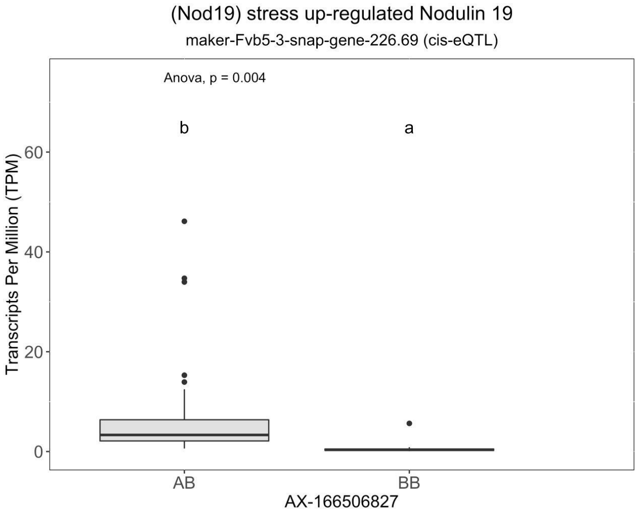
(Nod19) stress up-regulated Nodulin 19
maker-Fvb5-3-snap-gene-226.69 (cis-eQTL)
Anova, p = 0.004
b
a
Transcripts Per Million (TPM)
0
20
40
60
AB
BB
AX-166506827

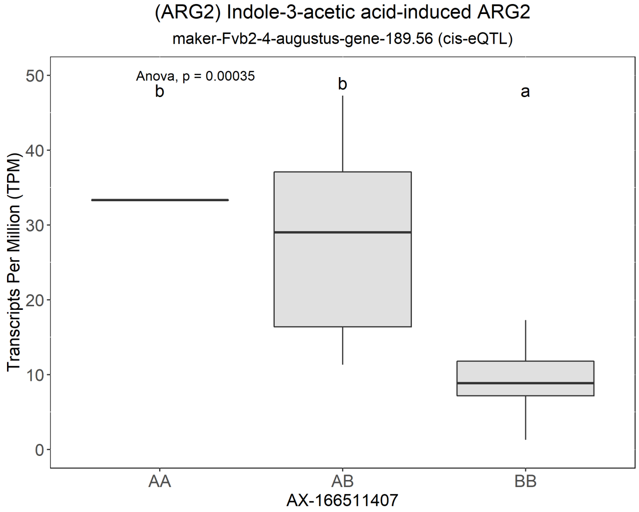
(ARG2) Indole-3-acetic acid-induced ARG2
maker-Fvb2-4-augustus-gene-189.56 (cis-eQTL)
Anova, p = 0.00035
b
b
a
Transcripts Per Million (TPM)
0
10
20
30
40
50
AA
AB
BB
AX-166511407

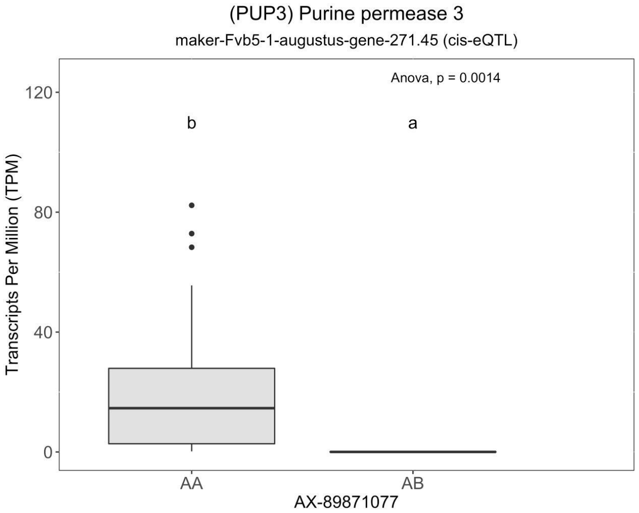
(PUP3) Purine permease 3
maker-Fvb5-1-augustus-gene-271.45 (cis-eQTL)
Anova, p = 0.0014
b
a
Transcripts Per Million (TPM)
0
40
80
120
AA
AB
AX-89871077

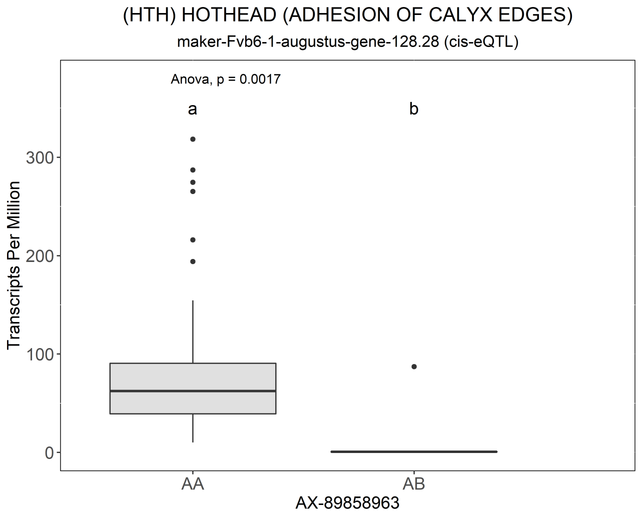
(HTH) HOTHEAD (ADHESION OF CALYX EDGES)
maker-Fvb6-1-augustus-gene-128.28 (cis-eQTL)
Anova, p = 0.0017
a
b
Transcripts Per Million
0
100
200
300
AA
AB
AX-89858963

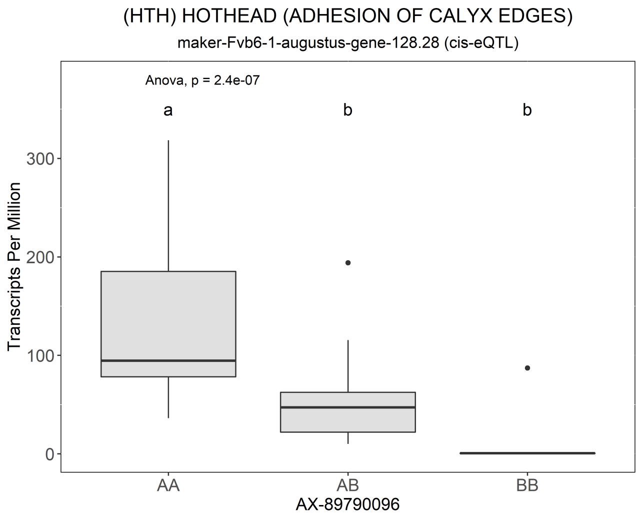
(HTH) HOTHEAD (ADHESION OF CALYX EDGES)
maker-Fvb6-1-augustus-gene-128.28 (cis-eQTL)
Anova, p = 2.4e-07
a
b
b
Transcripts Per Million
0
100
200
300
AA
AB
BB
AX-89790096

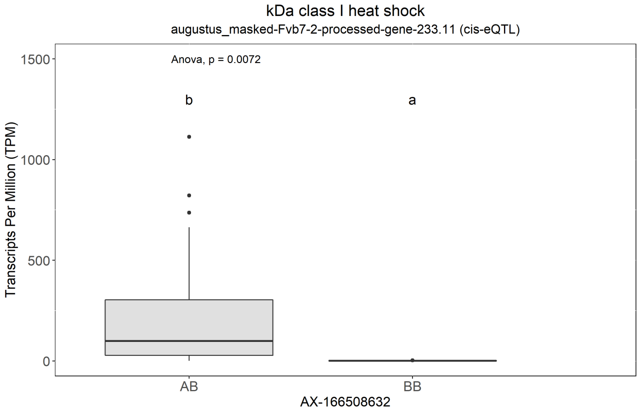
kDa class I heat shock
augustus_masked-Fvb7-2-processed-gene-233.11 (cis-eQTL)
Anova, p = 0.0072
b
a
Transcripts Per Million (TPM)
1500
1000
500
0
AB
BB
AX-166508632

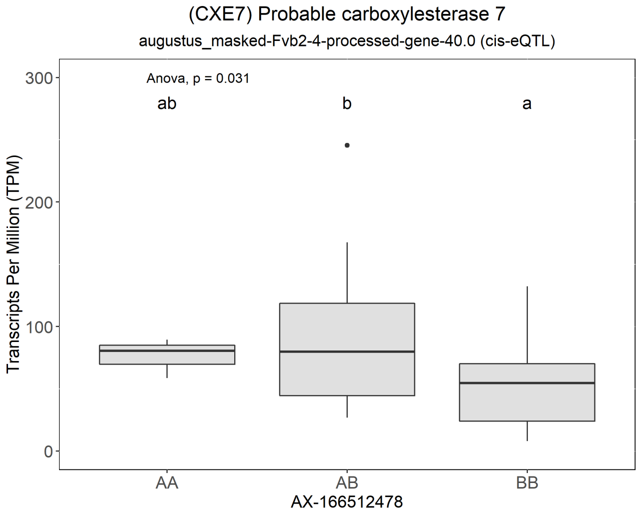
(CXE7) Probable carboxylesterase 7
augustus_masked-Fvb2-4-processed-gene-40.0 (cis-eQTL)
Anova, p = 0.031
ab
b
a
Transcripts Per Million (TPM)
300
200
100
0
AA
AB
BB
AX-166512478

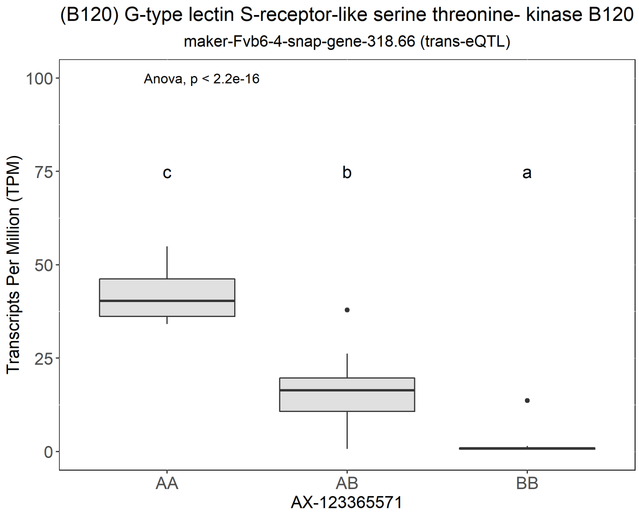
(B120) G-type lectin S-receptor-like serine threonine- kinase B120
maker-Fvb6-4-snap-gene-318.66 (trans-eQTL)
Anova, p < 2.2e-16
c
b
a
Transcripts Per Million (TPM)
100
75
50
25
0
AA
AB
BB
AX-123365571

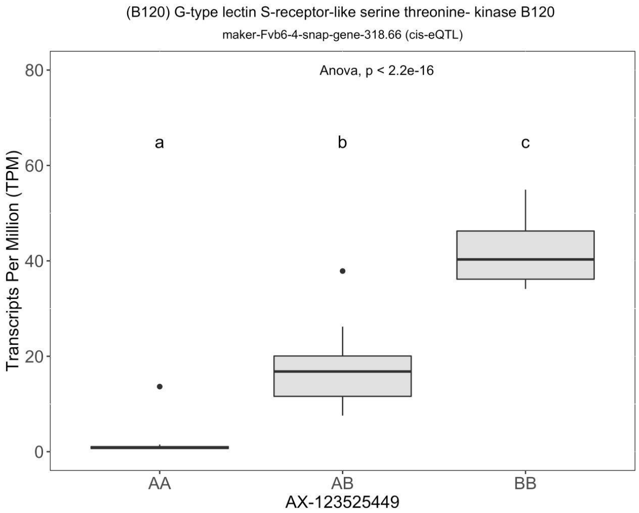
(B120) G-type lectin S-receptor-like serine threonine- kinase B120
maker-Fvb6-4-snap-gene-318.66 (cis-eQTL)
Anova, p < 2.2e-16
a
b
c
Transcripts Per Million (TPM)
80
60
40
20
0
AA
AB
BB
AX-123525449

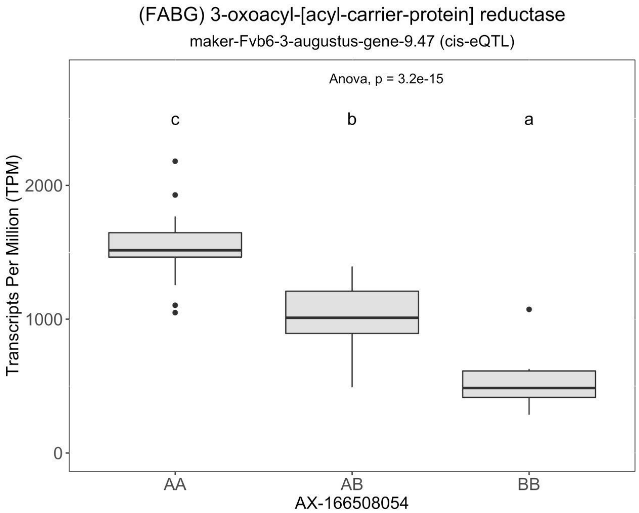
(FABG) 3-oxoacyl-[acyl-carrier-protein] reductase
maker-Fvb6-3-augustus-gene-9.47 (cis-eQTL)
Anova, p = 3.2e-15
c
b
a
Transcripts Per Million (TPM)
2000
1000
0
AA
AB
BB
AX-166508054

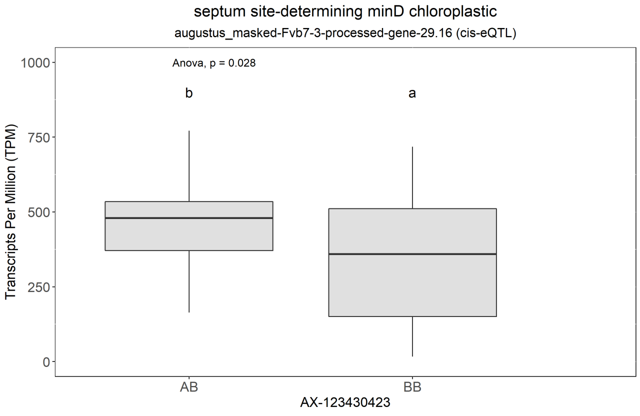
septum site-determining minD chloroplastic
augustus_masked-Fvb7-3-processed-gene-29.16 (cis-eQTL)
Anova, p = 0.028
b
a
Transcripts Per Million (TPM)
1000
750
500
250
0
AB
BB
AX-123430423

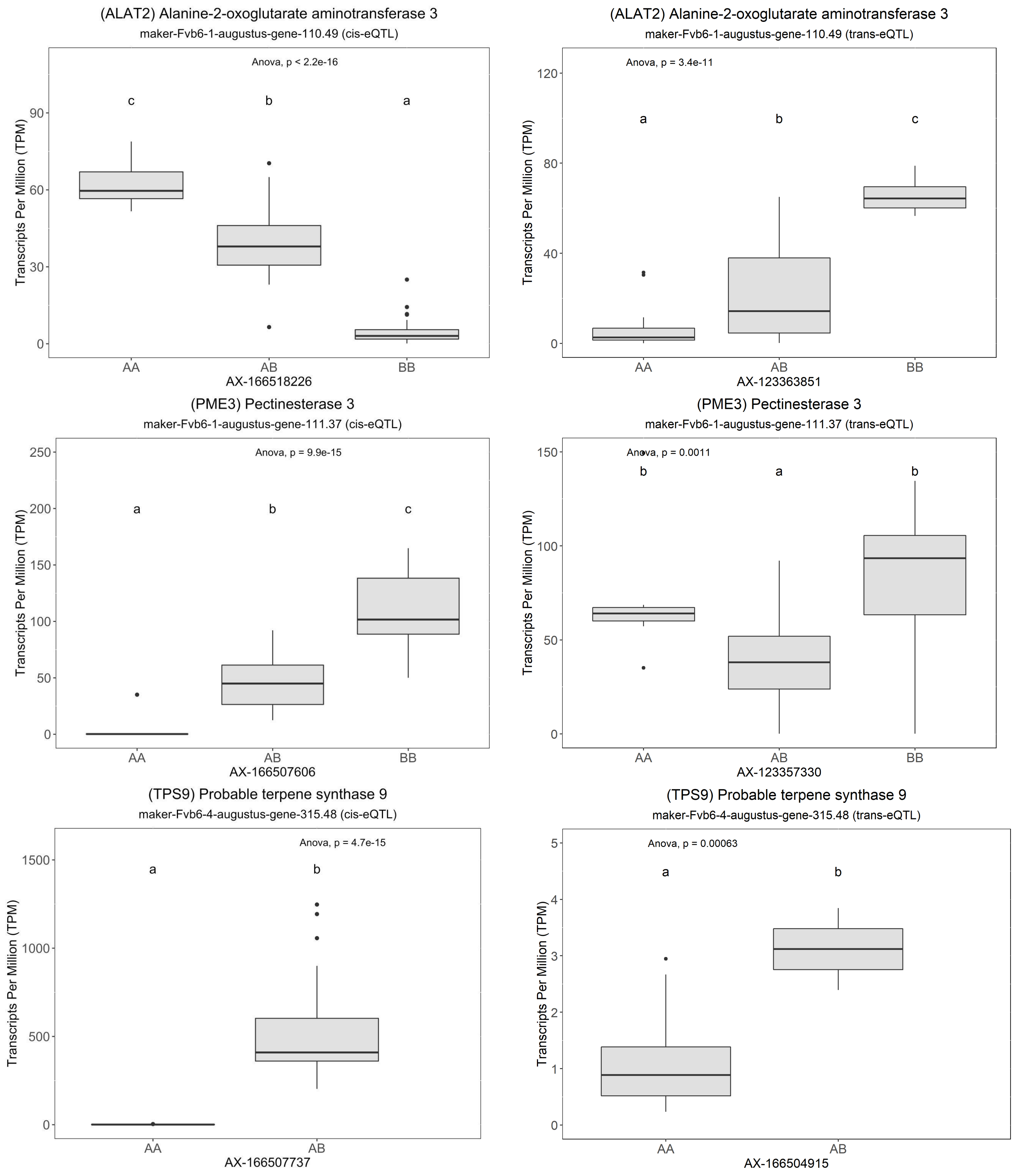
(ALAT2) Alanine-2-oxoglutarate aminotransferase 3
maker-Fvb6-1-augustus-gene-110.49 (cis-eQTL)
Anova, p < 2.2e-16
c
b
a
Transcripts Per Million (TPM)
0
30
60
90
AA
AB
BB
AX-166518226
(ALAT2) Alanine-2-oxoglutarate aminotransferase 3
maker-Fvb6-1-augustus-gene-110.49 (trans-eQTL)
Anova, p = 3.4e-11
a
b
c
Transcripts Per Million (TPM)
0
40
80
120
AA
AB
BB
AX-123363851
(PME3) Pectinesterase 3
maker-Fvb6-1-augustus-gene-111.37 (cis-eQTL)
Anova, p = 9.9e-15
a
b
c
Transcripts Per Million (TPM)
0
50
100
150
200
250
AA
AB
BB
AX-166507606
(PME3) Pectinesterase 3
maker-Fvb6-1-augustus-gene-111.37 (trans-eQTL)
Anova, p = 0.0011
b
a
b
Transcripts Per Million (TPM)
0
50
100
150
AA
AB
BB
AX-123357330
(TPS9) Probable terpene synthase 9
maker-Fvb6-4-augustus-gene-315.48 (cis-eQTL)
Anova, p = 4.7e-15
a
b
Transcripts Per Million (TPM)
0
500
1000
1500
AA
AB
AX-166507737
(TPS9) Probable terpene synthase 9
maker-Fvb6-4-augustus-gene-315.48 (trans-eQTL)
Anova, p = 0.00063
a
b
Transcripts Per Million (TPM)
0
1
2
3
4
5
AA
AB
AX-166504915

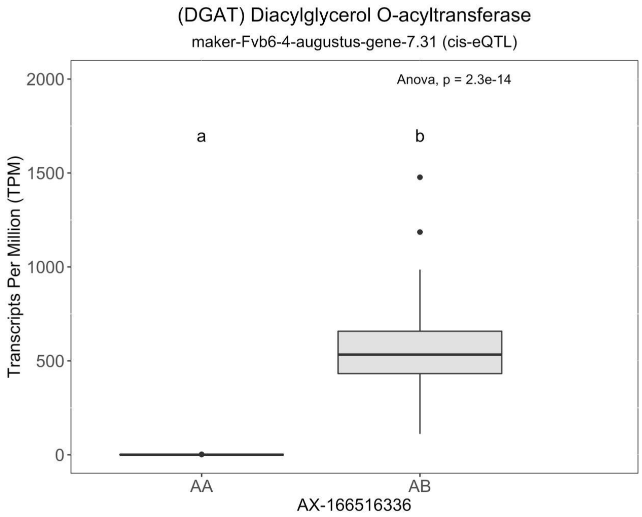
(DGAT) Diacylglycerol O-acyltransferase
maker-Fvb6-4-augustus-gene-7.31 (cis-eQTL)
Anova, p = 2.3e-14
a
b
Transcripts Per Million (TPM)
2000
1500
1000
500
0
AA
AB
AX-166516336

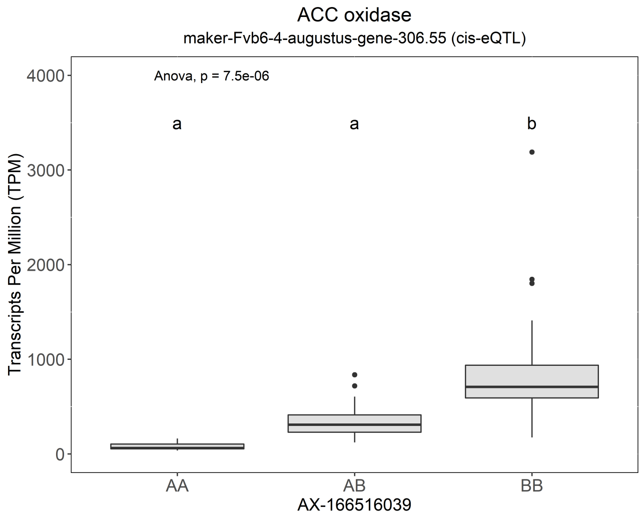
ACC oxidase
maker-Fvb6-4-augustus-gene-306.55 (cis-eQTL)
Anova, p = 7.5e-06
a
a
b
Transcripts Per Million (TPM)
4000
3000
2000
1000
0
AA
AB
BB
AX-166516039

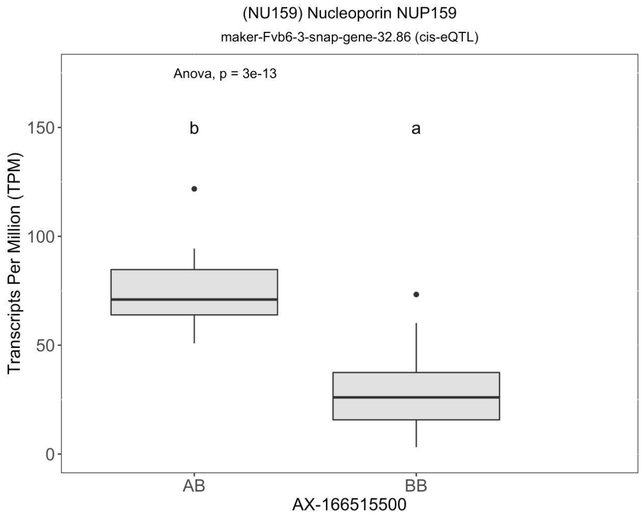
(NU159) Nucleoporin NUP159
maker-Fvb6-3-snap-gene-32.86 (cis-eQTL)
Anova, p = 3e-13
b
a
Transcripts Per Million (TPM)
150
100
50
0
AB
BB
AX-166515500

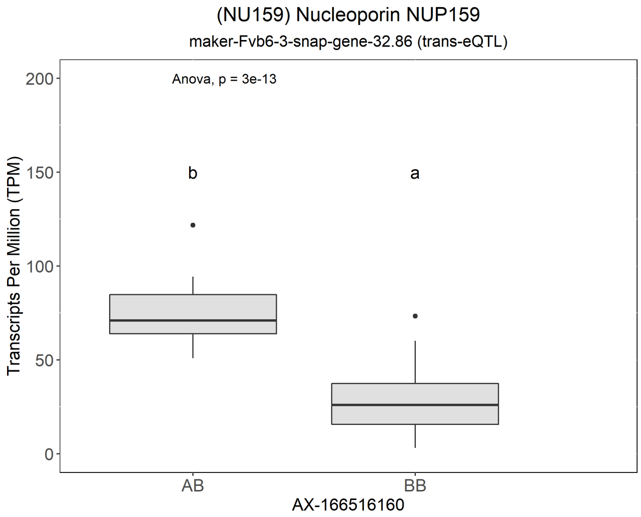
(NU159) Nucleoporin NUP159
maker-Fvb6-3-snap-gene-32.86 (trans-eQTL)
Anova, p = 3e-13
b
a
Transcripts Per Million (TPM)
200
150
100
50
0
AB
BB
AX-166516160

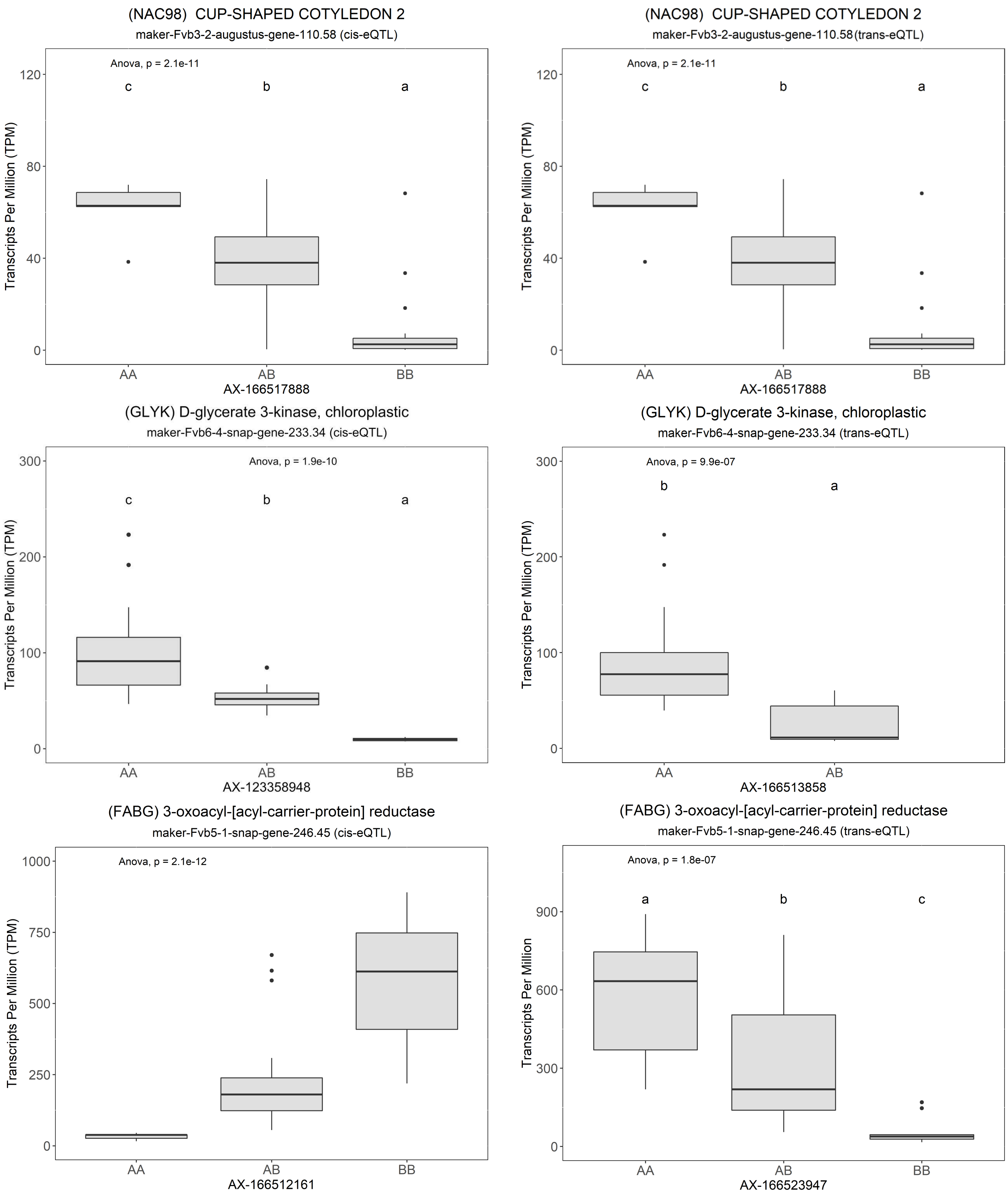
(NAC98) CUP-SHAPED COTYLEDON 2
maker-Fvb3-2-augustus-gene-110.58 (cis-eQTL)
Anova, p = 2.1e-11
c
b
a
Transcripts Per Million (TPM)
120
80
40
0
AA
AB
BB
AX-166517888
(NAC98) CUP-SHAPED COTYLEDON 2
maker-Fvb3-2-augustus-gene-110.58(trans-eQTL)
Anova, p = 2.1e-11
c
b
a
Transcripts Per Million (TPM)
120
80
40
0
AA
AB
BB
AX-166517888
(GLYK) D-glycerate 3-kinase, chloroplastic
maker-Fvb6-4-snap-gene-233.34 (cis-eQTL)
Anova, p = 1.9e-10
c
b
a
Transcripts Per Million (TPM)
300
200
100
0
AA
AB
BB
AX-123358948
(GLYK) D-glycerate 3-kinase, chloroplastic
maker-Fvb6-4-snap-gene-233.34 (trans-eQTL)
Anova, p = 9.9e-07
b
a
Transcripts Per Million (TPM)
300
200
100
0
AA
AB
AX-166513858
(FABG) 3-oxoacyl-[acyl-carrier-protein] reductase
maker-Fvb5-1-snap-gene-246.45 (cis-eQTL)
Anova, p = 2.1e-12
Transcripts Per Million (TPM)
1000
750
500
250
0
AA
AB
BB
AX-166512161
(FABG) 3-oxoacyl-[acyl-carrier-protein] reductase
maker-Fvb5-1-snap-gene-246.45 (trans-eQTL)
Anova, p = 1.8e-07
a
b
c
Transcripts Per Million
900
600
300
0
AA
AB
BB
AX-166523947

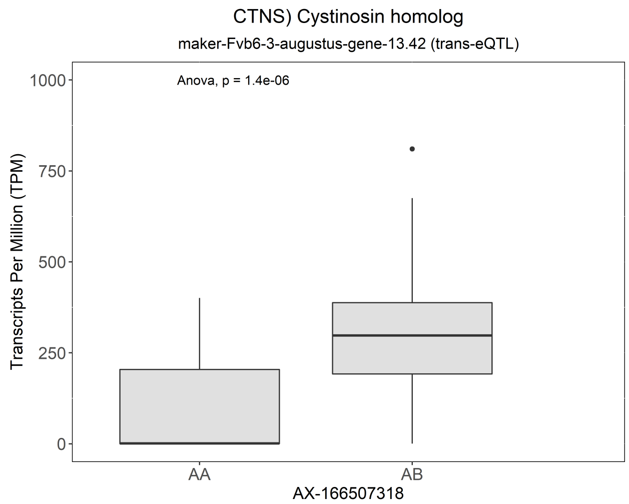
CTNS) Cystinosin homolog
maker-Fvb6-3-augustus-gene-13.42 (trans-eQTL)
Anova, p = 1.4e-06
Transcripts Per Million (TPM)
1000
750
500
250
0
AA
AB
AX-166507318

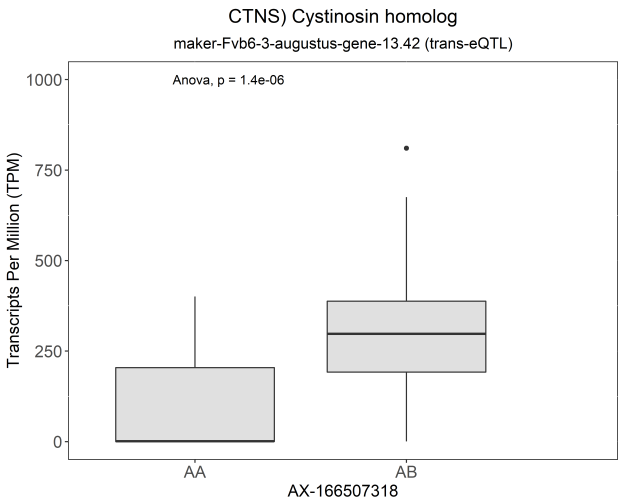
CTNS) Cystinosin homolog
maker-Fvb6-3-augustus-gene-13.42 (trans-eQTL)
Anova, p = 1.4e-06
Transcripts Per Million (TPM)
1000
750
500
250
0
AA
AB
AX-166507318

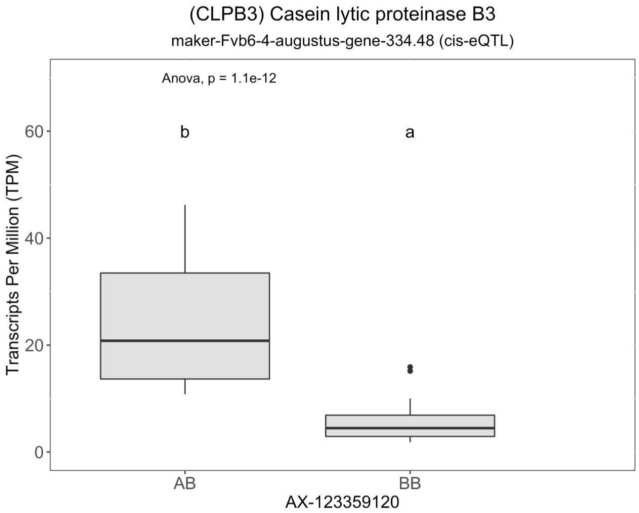
(CLPB3) Casein lytic proteinase B3
maker-Fvb6-4-augustus-gene-334.48 (cis-eQTL)
Anova, p = 1.1e-12
b
a
Transcripts Per Million (TPM)
60
40
20
0
AB
BB
AX-123359120

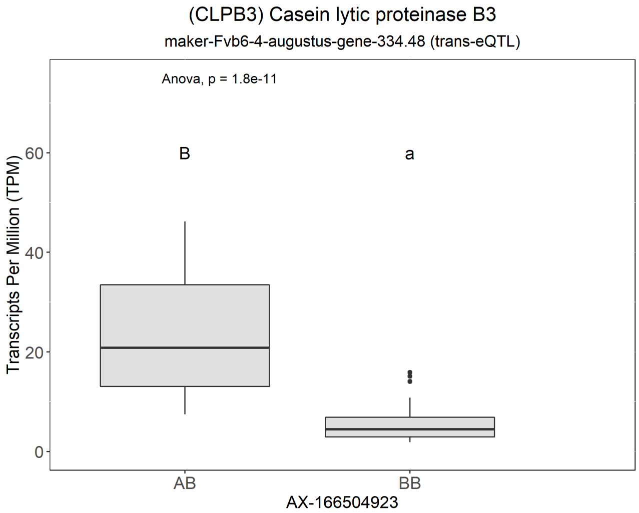
(CLPB3) Casein lytic proteinase B3
maker-Fvb6-4-augustus-gene-334.48 (trans-eQTL)
Anova, p = 1.8e-11
B
a
Transcripts Per Million (TPM)
60
40
20
0
AB
BB
AX-166504923

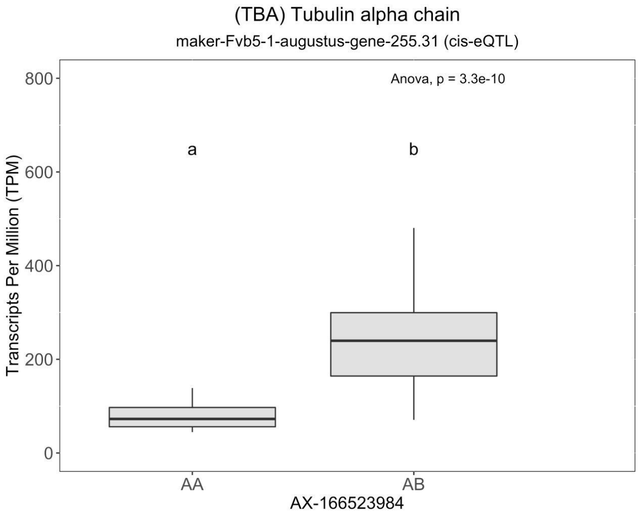
(TBA) Tubulin alpha chain
maker-Fvb5-1-augustus-gene-255.31 (cis-eQTL)
Anova, p = 3.3e-10
a
b
Transcripts Per Million (TPM)
800
600
400
200
0
AA
AB
AX-166523984

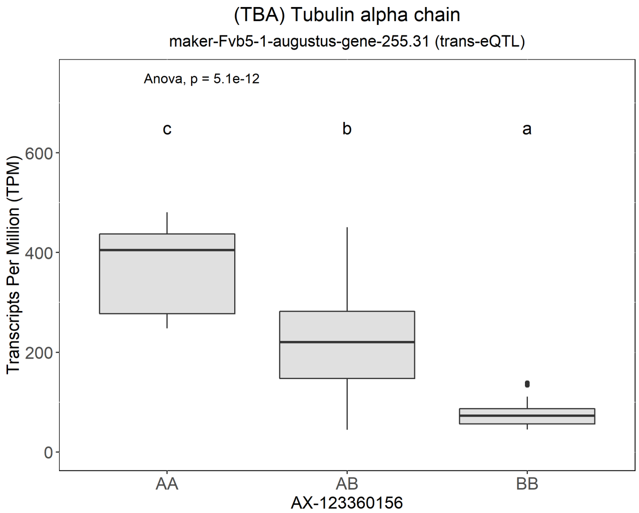
(TBA) Tubulin alpha chain
maker-Fvb5-1-augustus-gene-255.31 (trans-eQTL)
Anova, p = 5.1e-12
c
b
a
Transcripts Per Million (TPM)
600
400
200
0
AA
AB
BB
AX-123360156

(DES1L) Sphingolipid delta(4)-desaturase DES1-like
maker-Fvb3-2-augustus-gene-10.41 (cis-eQTL)

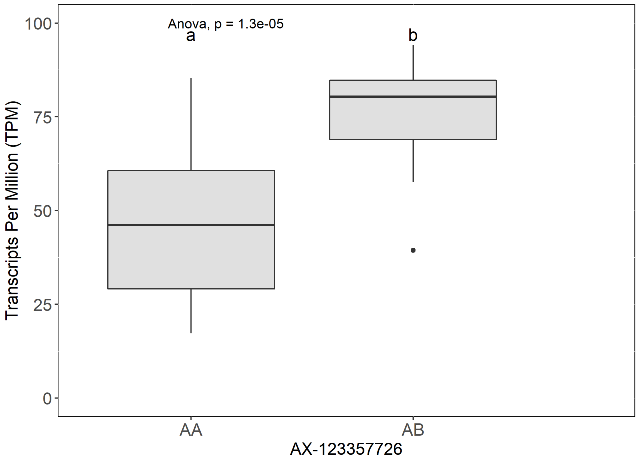

(DES1L) Sphingolipid delta(4)-desaturase DES1-like
maker-Fvb3-2-augustus-gene-10.41 (trans-eQTL)

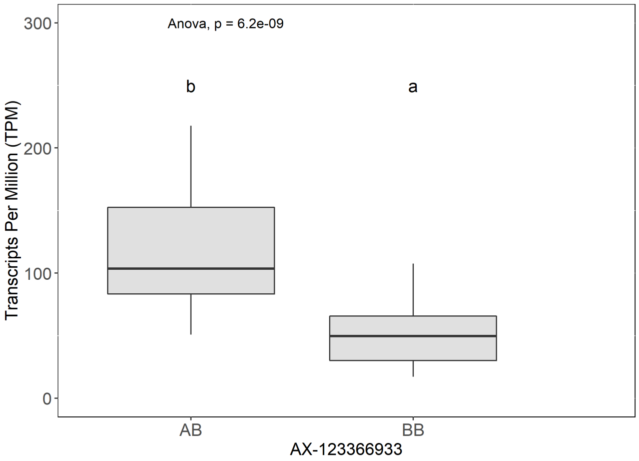

(HTH) HOTHEAD (ADHESION OF CALYX EDGES)
maker-Fvb6-1-augustus-gene-128.28 (cis-eQTL)

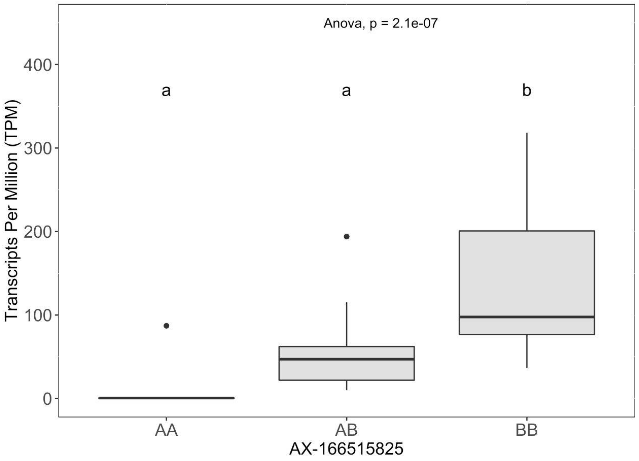

(HTH) HOTHEAD (ADHESION OF CALYX EDGES)
maker-Fvb6-1-augustus-gene-128.28 (cis-eQTL)

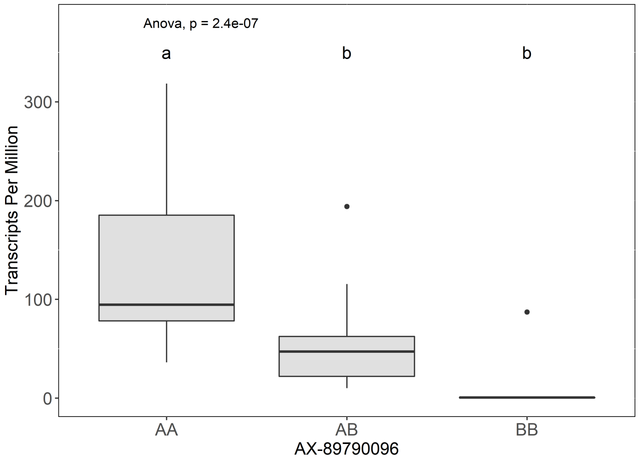

(CUCM1) Cucumisin allergen (subtilisin□like endopeptidase)
maker-Fvb7-2-augustus-gene-139.49 (cis-eQTL)

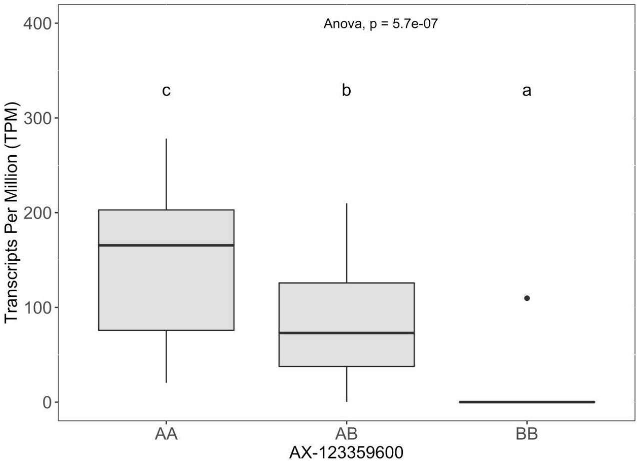

(CUCM1) Cucumisin allergen (subtilisin?like endopeptidase)
maker-Fvb7-2-augustus-gene-139.49 (trans-eQTL)

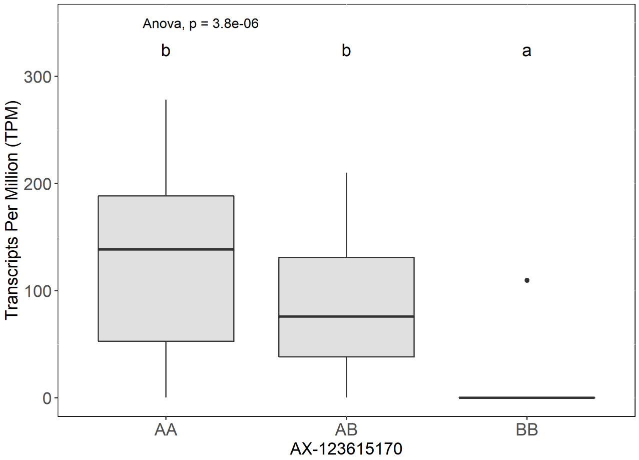

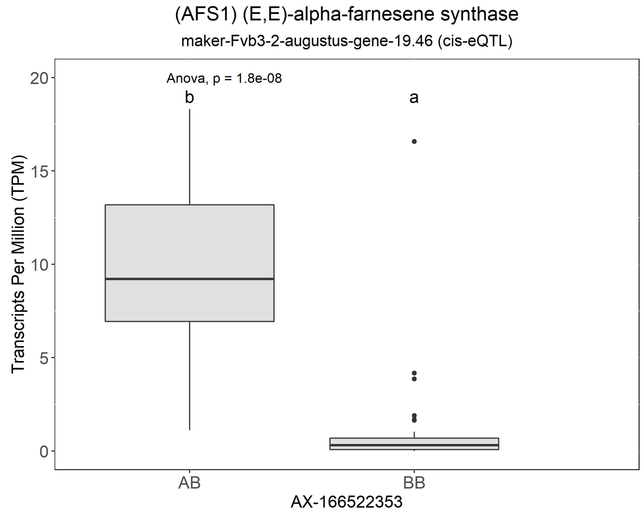
(AFS1) (E,E)-alpha-farnesene synthase
maker-Fvb3-2-augustus-gene-19.46 (cis-eQTL)
Anova, p = 1.8e-08
b
a
Transcripts Per Million (TPM)
AB
BB
AX-166522353

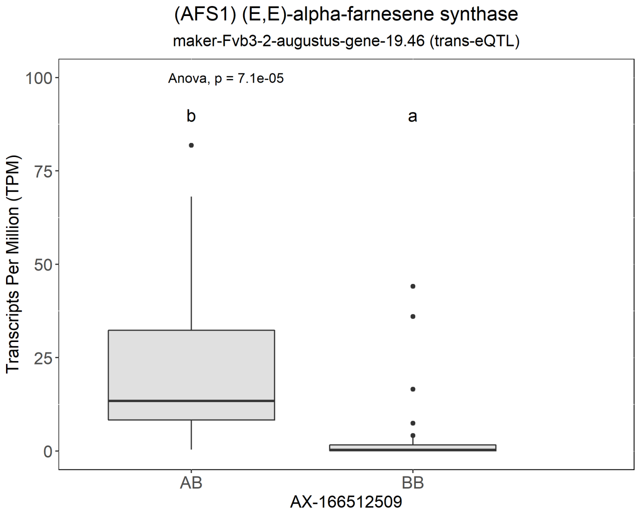
(AFS1) (E,E)-alpha-farnesene synthase
maker-Fvb3-2-augustus-gene-19.46 (trans-eQTL)
Anova, p = 7.1e-05
b
a
Transcripts Per Million (TPM)
AB
BB
AX-166512509

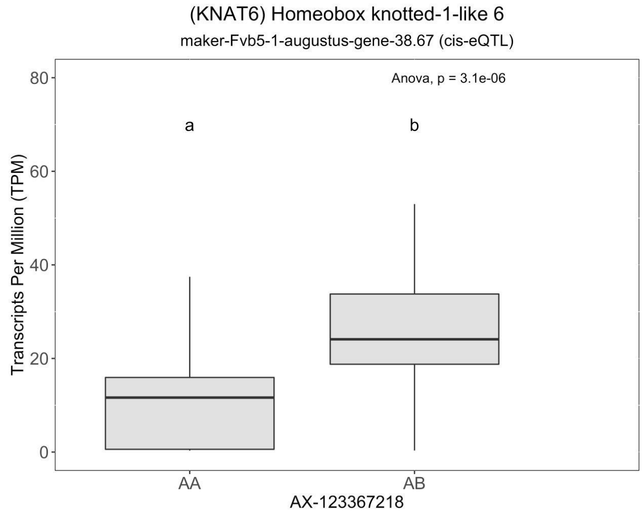
(KNAT6) Homeobox knotted-1-like 6
maker-Fvb5-1-augustus-gene-38.67 (cis-eQTL)
Anova, p = 3.1e-06
a
b
Transcripts Per Million (TPM)
AA
AB
AX-123367218

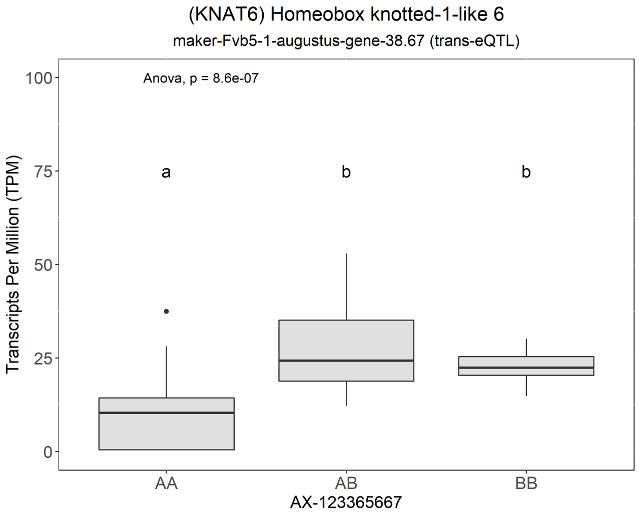
(KNAT6) Homeobox knotted-1-like 6
maker-Fvb5-1-augustus-gene-38.67 (trans-eQTL)
Anova, p = 8.6e-07
a
b
b
Transcripts Per Million (TPM)
AA
AB
BB
AX-123365667

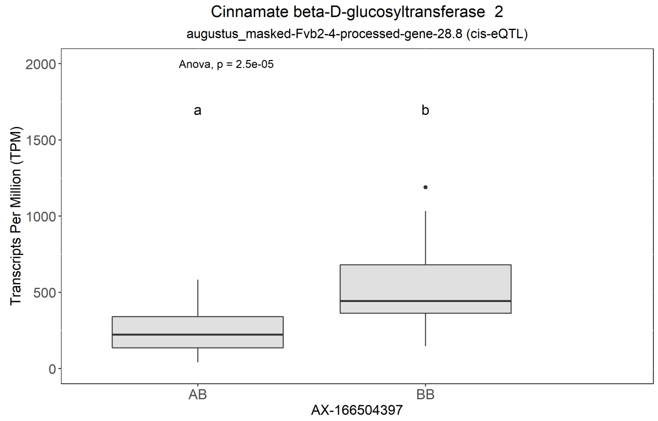
Cinnamate beta-D-glucosyltransferase 2
augustus_masked-Fvb2-4-processed-gene-28.8 (cis-eQTL)
Anova, p = 2.5e-05
a
b
Transcripts Per Million (TPM)
AB
BB
AX-166504397

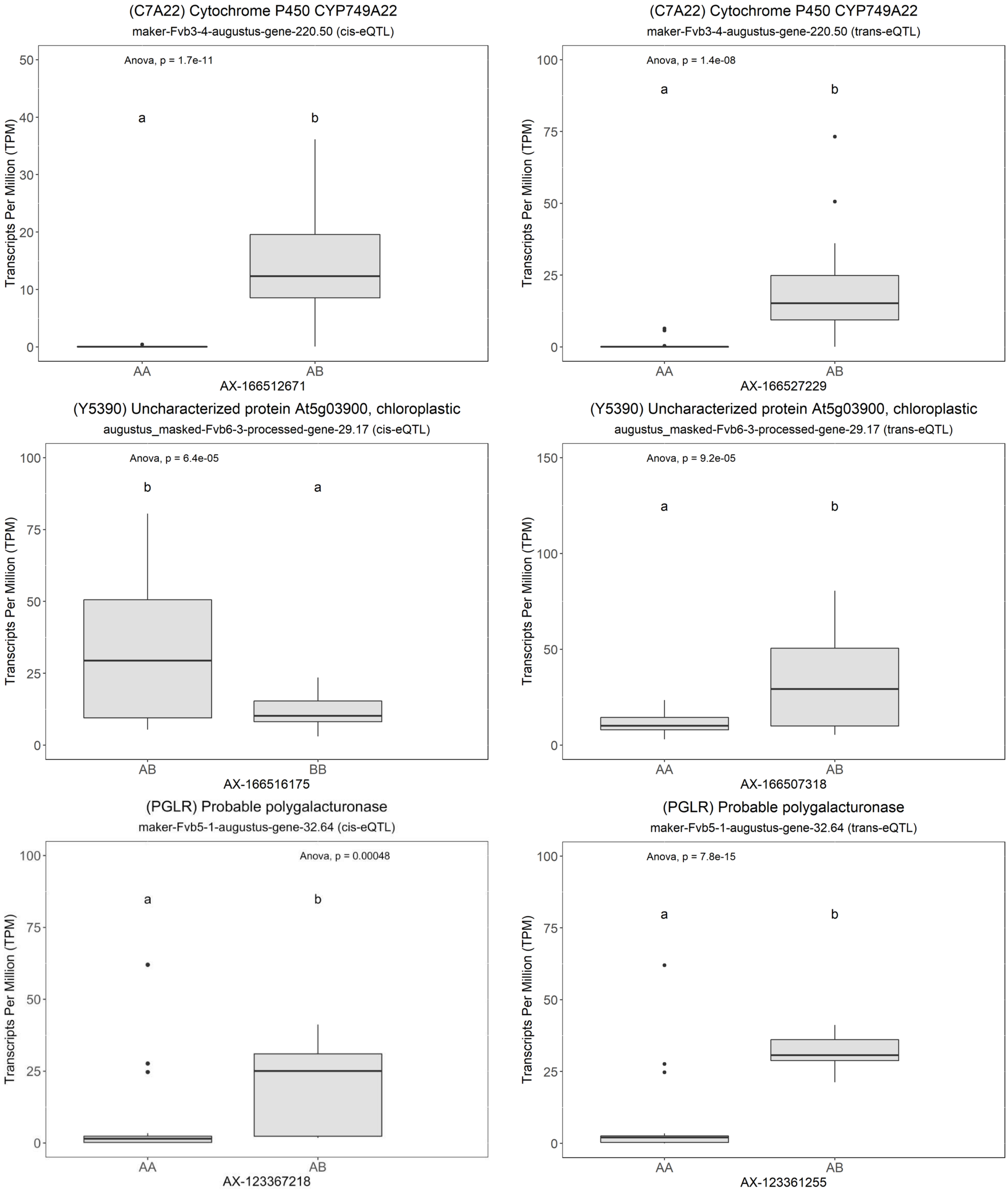
(C7A22) Cytochrome P450 CYP749A22
maker-Fvb3-4-augustus-gene-220.50 (cis-eQTL)
Anova, p = 1.7e-11
a
b
Transcripts Per Million (TPM)
0
10
20
30
40
50
AA
AB
AX-166512671
(C7A22) Cytochrome P450 CYP749A22
maker-Fvb3-4-augustus-gene-220.50 (trans-eQTL)
Anova, p = 1.4e-08
a
b
Transcripts Per Million (TPM)
0
25
50
75
100
AA
AB
AX-166527229
(Y5390) Uncharacterized protein At5g03900, chloroplastic
augustus_masked-Fvb6-3-processed-gene-29.17 (cis-eQTL)
Anova, p = 6.4e-05
b
a
Transcripts Per Million (TPM)
0
25
50
75
100
AB
BB
AX-166516175
(Y5390) Uncharacterized protein At5g03900, chloroplastic
augustus_masked-Fvb6-3-processed-gene-29.17 (trans-eQTL)
Anova, p = 9.2e-05
a
b
Transcripts Per Million (TPM)
0
50
100
150
AA
AB
AX-166507318
(PGLR) Probable polygalacturonase
maker-Fvb5-1-augustus-gene-32.64 (cis-eQTL)
Anova, p = 0.00048
a
b
Transcripts Per Million (TPM)
0
25
50
75
100
AA
AB
AX-123367218
(PGLR) Probable polygalacturonase
maker-Fvb5-1-augustus-gene-32.64 (trans-eQTL)
Anova, p = 7.8e-15
a
b
Transcripts Per Million (TPM)
0
25
50
75
100
AA
AB
AX-123361255

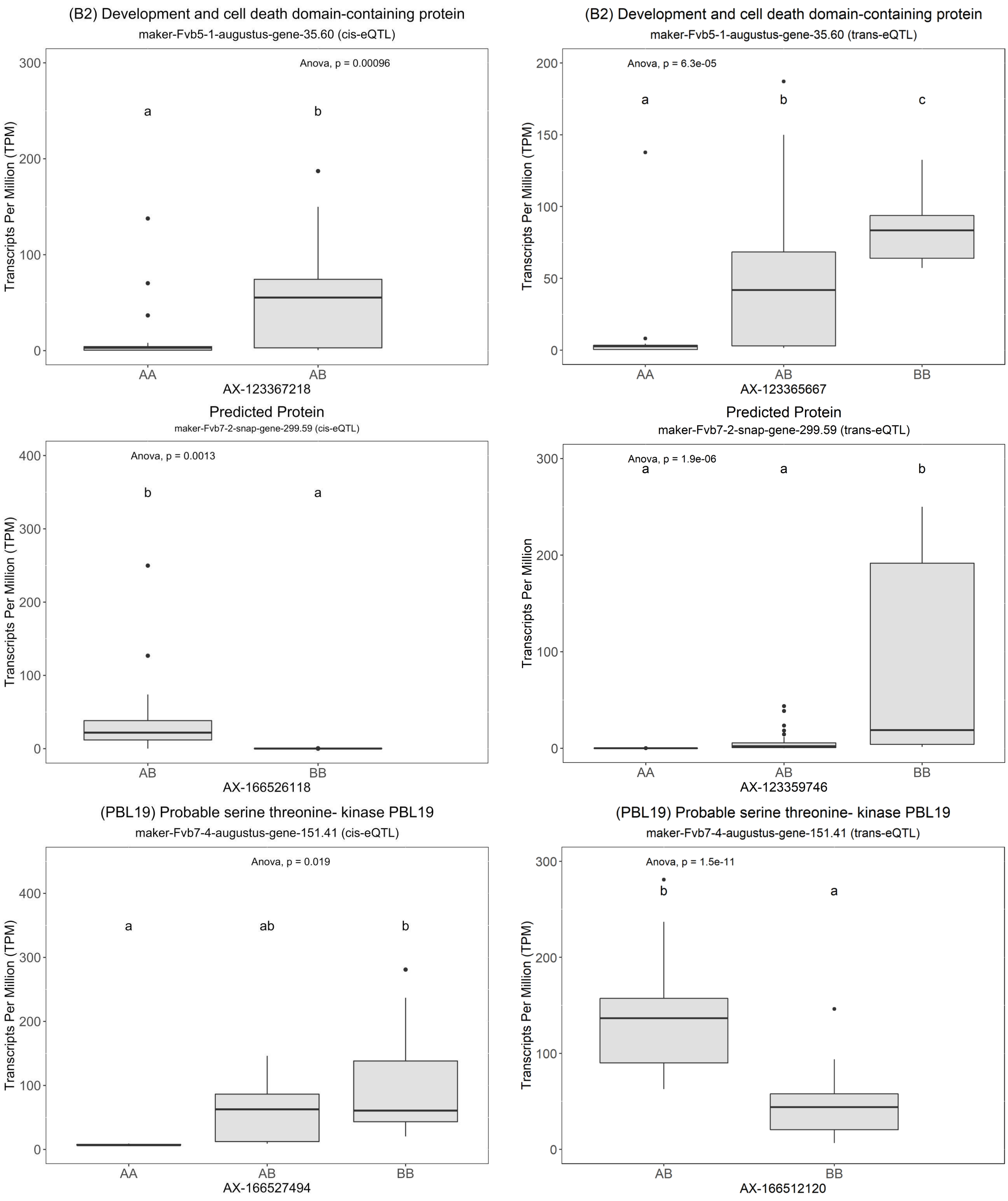
(B2) Development and cell death domain-containing protein
maker-Fvb5-1-augustus-gene-35.60 (cis-eQTL)
Anova, p = 0.00096
a
b
Transcripts Per Million (TPM)
AA
AB
AX-123367218
(B2) Development and cell death domain-containing protein
maker-Fvb5-1-augustus-gene-35.60 (trans-eQTL)
Anova, p = 6.3e-05
a
b
c
Transcripts Per Million (TPM)
AA
AB
BB
AX-123365667
Predicted Protein
maker-Fvb7-2-snap-gene-299.59 (cis-eQTL)
Anova, p = 0.0013
b
a
Transcripts Per Million (TPM)
AB
BB
AX-166526118
Predicted Protein
maker-Fvb7-2-snap-gene-299.59 (trans-eQTL)
Anova, p = 1.9e-06
a
a
b
Transcripts Per Million
AA
AB
BB
AX-123359746
(PBL19) Probable serine threonine- kinase PBL19
maker-Fvb7-4-augustus-gene-151.41 (cis-eQTL)
Anova, p = 0.019
a
ab
b
Transcripts Per Million (TPM)
AA
AB
BB
AX-166527494
(PBL19) Probable serine threonine- kinase PBL19
maker-Fvb7-4-augustus-gene-151.41 (trans-eQTL)
Anova, p = 1.5e-11
b
a
Transcripts Per Million (TPM)
AB
BB
AX-166512120

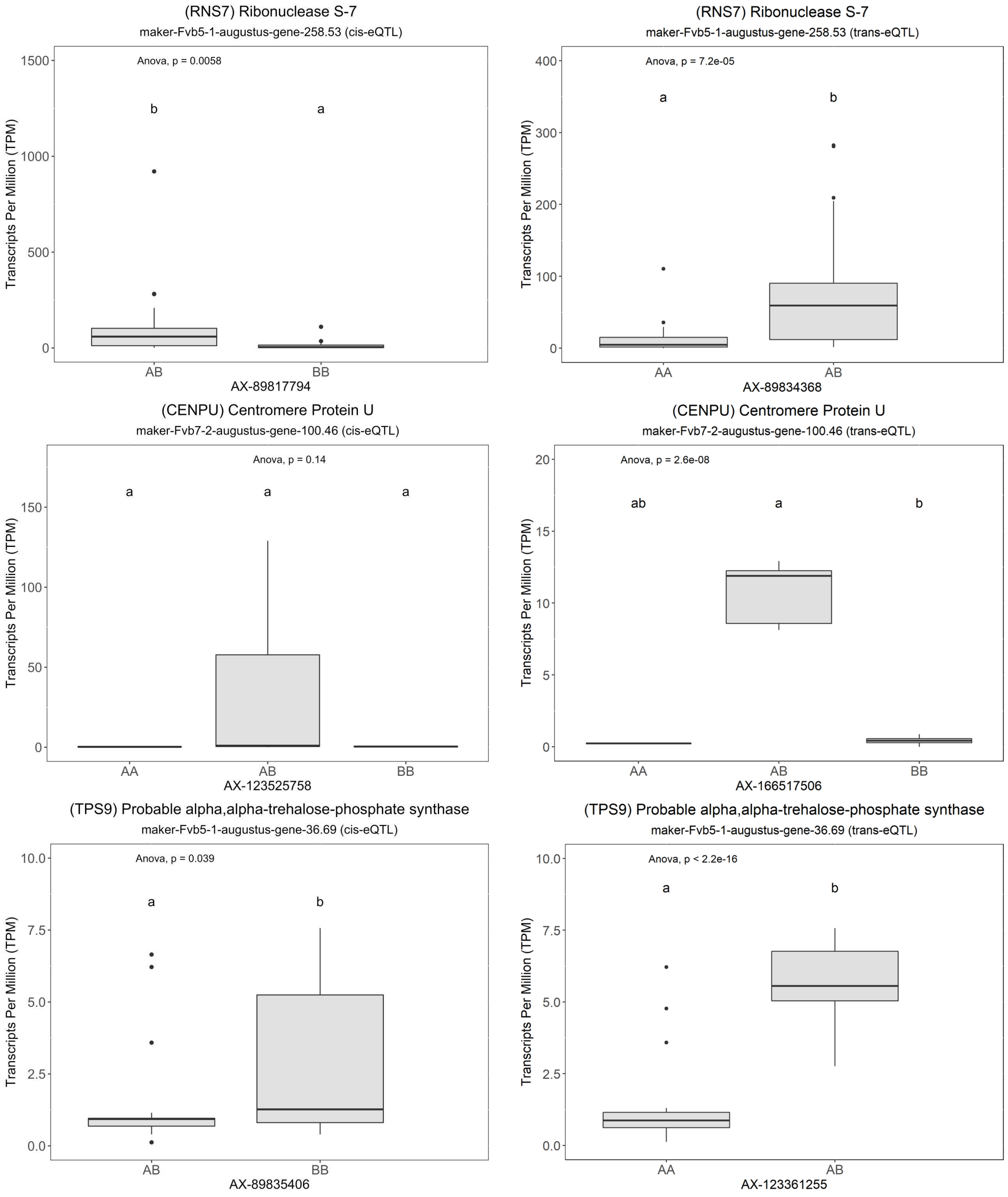
(RNS7) Ribonuclease S-7
maker-Fvb5-1-augustus-gene-258.53 (cis-eQTL)
Anova, p = 0.0058
b
a
Transcripts Per Million (TPM)
1500
1000
500
0
AB
BB
AX-89817794
(RNS7) Ribonuclease S-7
maker-Fvb5-1-augustus-gene-258.53 (trans-eQTL)
Anova, p = 7.2e-05
a
b
Transcripts Per Million (TPM)
400
300
200
100
0
AA
AB
AX-89834368
(CENPU) Centromere Protein U
maker-Fvb7-2-augustus-gene-100.46 (cis-eQTL)
Anova, p = 0.14
a
a
a
Transcripts Per Million (TPM)
150
100
50
0
AA
AB
BB
AX-123525758
(CENPU) Centromere Protein U
maker-Fvb7-2-augustus-gene-100.46 (trans-eQTL)
Anova, p = 2.6e-08
ab
a
b
Transcripts Per Million (TPM)
20
15
10
5
0
AA
AB
BB
AX-166517506
(TPS9) Probable alpha,alpha-trehalose-phosphate synthase
maker-Fvb5-1-augustus-gene-36.69 (cis-eQTL)
Anova, p = 0.039
a
b
Transcripts Per Million (TPM)
10.0
7.5
5.0
2.5
0.0
AB
BB
AX-89835406
(TPS9) Probable alpha,alpha-trehalose-phosphate synthase
maker-Fvb5-1-augustus-gene-36.69 (trans-eQTL)
Anova, p < 2.2e-16
a
b
Transcripts Per Million (TPM)
10.0
7.5
5.0
2.5
0.0
AA
AB
AX-123361255

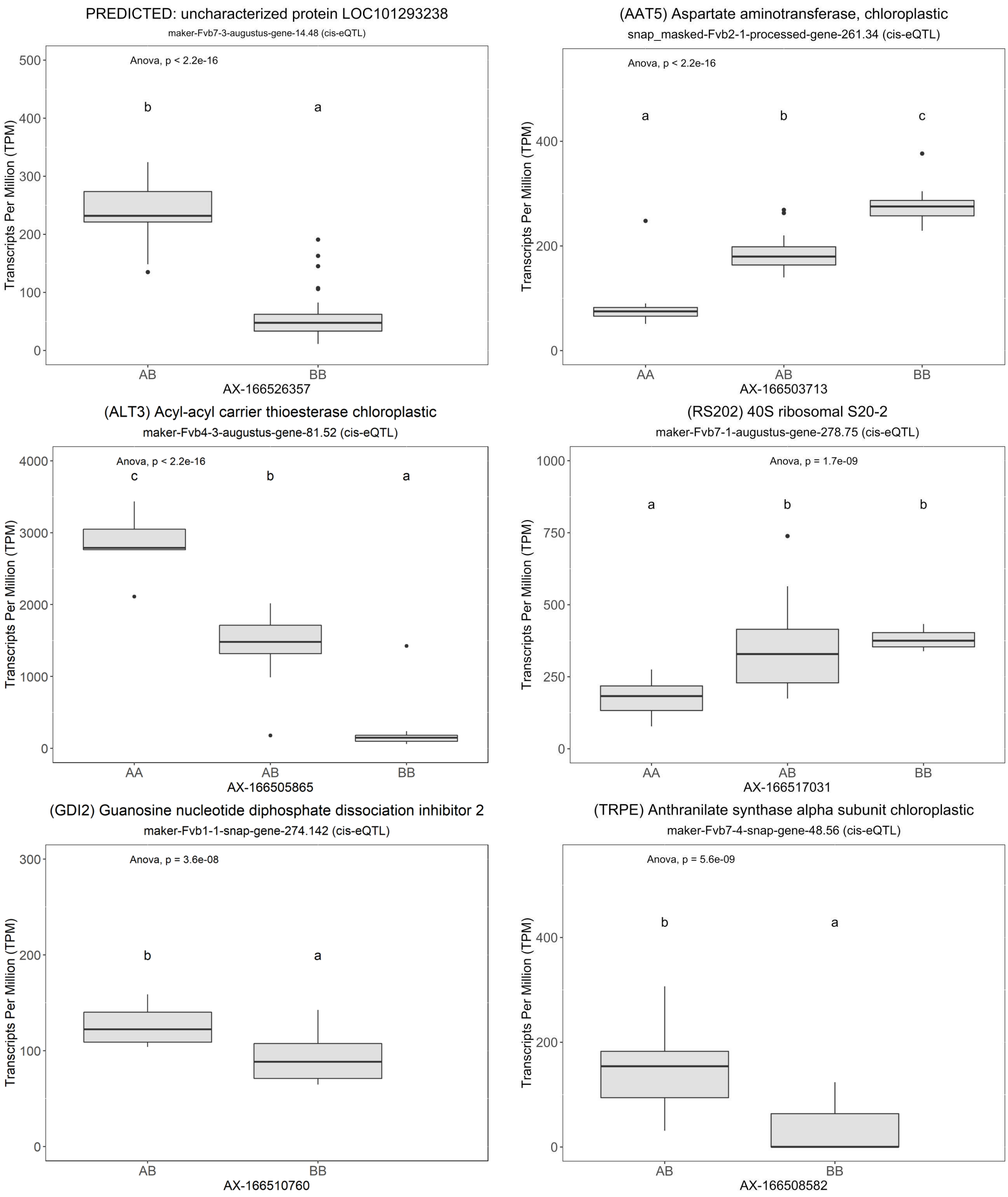
PREDICTED: uncharacterized protein LOC101293238
maker-Fvb7-3-augustus-gene-14.48 (cis-eQTL)
Anova, p < 2.2e-16
Transcripts Per Million (TPM)
AX-166526357
(AAT5) Aspartate aminotransferase, chloroplastic
snap_masked-Fvb2-1-processed-gene-261.34 (cis-eQTL)
Anova, p < 2.2e-16
Transcripts Per Million (TPM)
AX-166503713
(ALT3) Acyl-acyl carrier thioesterase chloroplastic
maker-Fvb4-3-augustus-gene-81.52 (cis-eQTL)
Anova, p < 2.2e-16
Transcripts Per Million (TPM)
AX-166505865
(RS202) 40S ribosomal S20-2
maker-Fvb7-1-augustus-gene-278.75 (cis-eQTL)
Anova, p = 1.7e-09
Transcripts Per Million (TPM)
AX-166517031
(GDI2) Guanosine nucleotide diphosphate dissociation inhibitor 2
maker-Fvb1-1-snap-gene-274.142 (cis-eQTL)
Anova, p = 3.6e-08
Transcripts Per Million (TPM)
AX-166510760
(TRPE) Anthranilate synthase alpha subunit chloroplastic
maker-Fvb7-4-snap-gene-48.56 (cis-eQTL)
Anova, p = 5.6e-09
Transcripts Per Million (TPM)
AX-166508582

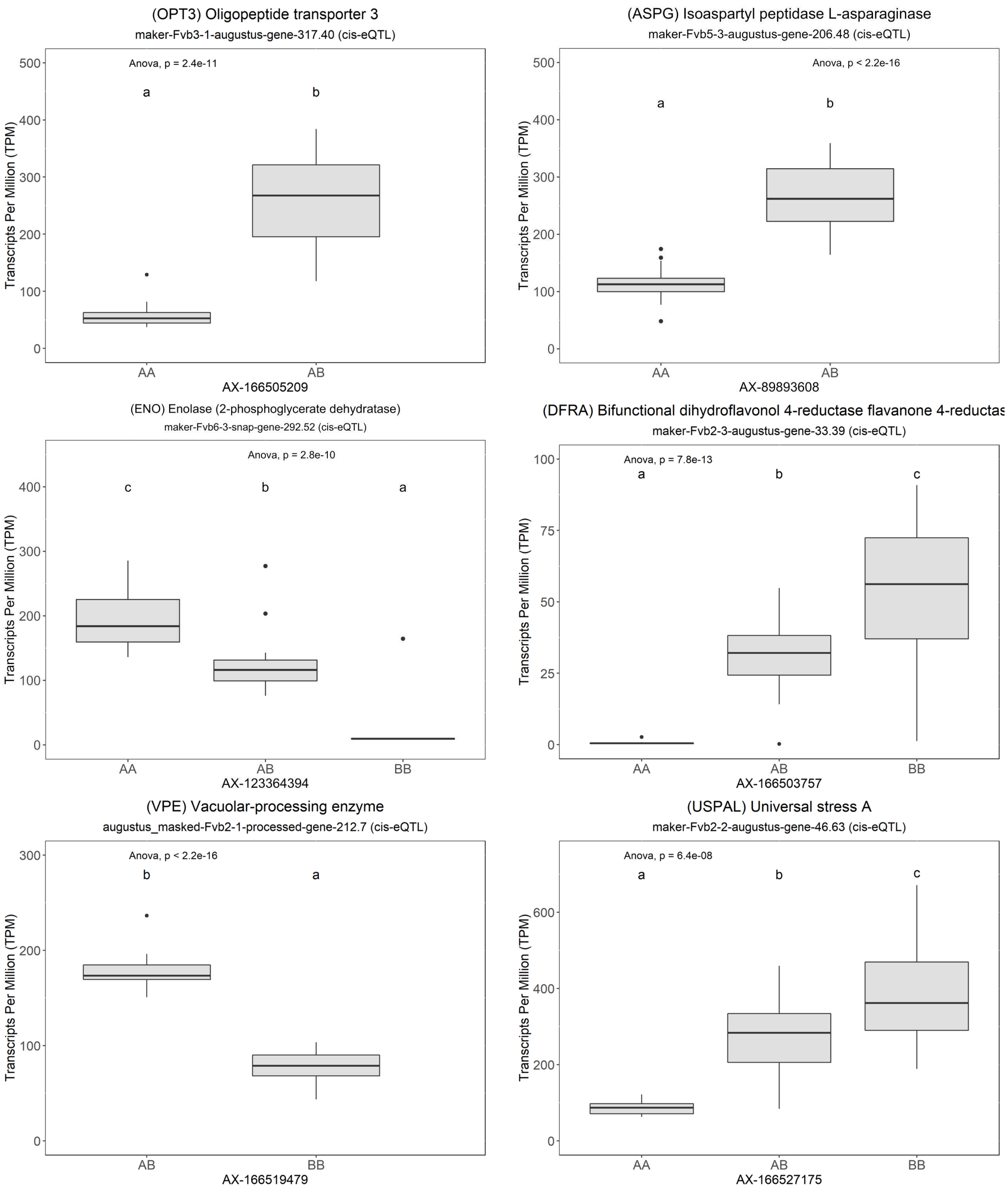
(OPT3) Oligopeptide transporter 3
maker-Fvb3-1-augustus-gene-317.40 (cis-eQTL)
Anova, p = 2.4e-11
a
b
Transcripts Per Million (TPM)
0
100
200
300
400
500
AA
AB
AX-166505209
(ASPG) Isoaspartyl peptidase L-asparaginase
maker-Fvb5-3-augustus-gene-206.48 (cis-eQTL)
Anova, p < 2.2e-16
a
b
Transcripts Per Million (TPM)
0
100
200
300
400
500
AA
AB
AX-89893608
(ENO) Enolase (2-phosphoglycerate dehydratase)
maker-Fvb6-3-snap-gene-292.52 (cis-eQTL)
Anova, p = 2.8e-10
c
b
a
Transcripts Per Million (TPM)
0
100
200
300
400
AA
AB
BB
AX-123364394
(DFRA) Bifunctional dihydroflavonol 4-reductase flavanone 4-reductas
maker-Fvb2-3-augustus-gene-33.39 (cis-eQTL)
Anova, p = 7.8e-13
a
b
c
Transcripts Per Million (TPM)
0
25
50
75
100
AA
AB
BB
AX-166503757
(VPE) Vacuolar-processing enzyme
augustus_masked-Fvb2-1-processed-gene-212.7 (cis-eQTL)
Anova, p < 2.2e-16
b
a
Transcripts Per Million (TPM)
0
100
200
300
AB
BB
AX-166519479
(USPAL) Universal stress A
maker-Fvb2-2-augustus-gene-46.63 (cis-eQTL)
Anova, p = 6.4e-08
a
b
c
Transcripts Per Million (TPM)
0
200
400
600
AA
AB
BB
AX-166527175

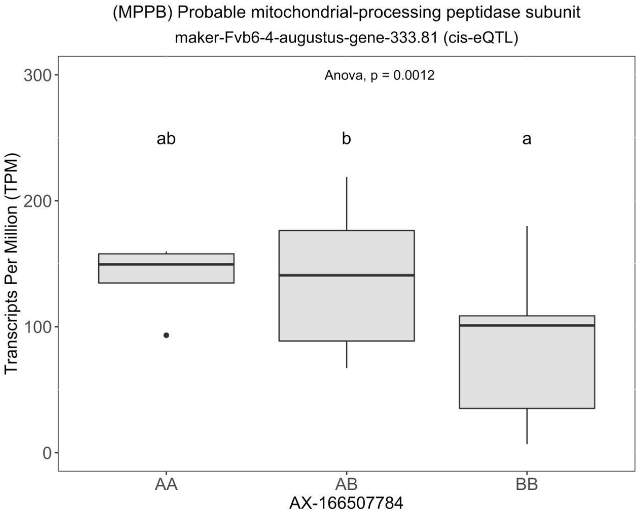
(MPPB) Probable mitochondrial-processing peptidase subunit
maker-Fvb6-4-augustus-gene-333.81 (cis-eQTL)
Anova, p = 0.0012
ab
b
a
Transcripts Per Million (TPM)
300
200
100
0
AA
AB
BB
AX-166507784

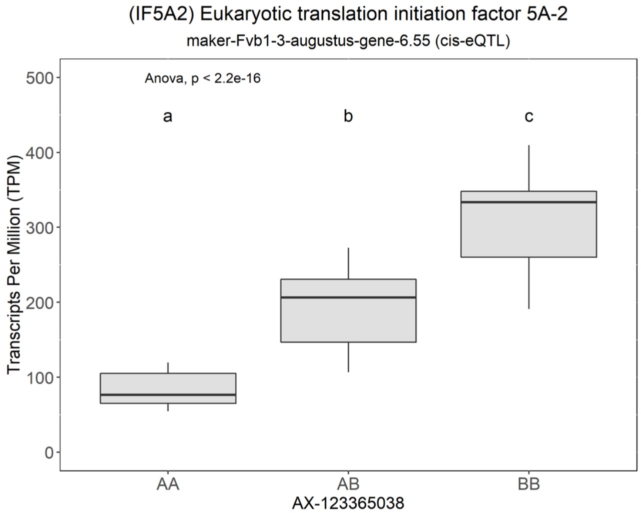
(IF5A2) Eukaryotic translation initiation factor 5A-2
maker-Fvb1-3-augustus-gene-6.55 (cis-eQTL)
Anova, p < 2.2e-16
a
b
c
Transcripts Per Million (TPM)
500
400
300
200
100
0
AA
AB
BB
AX-123365038

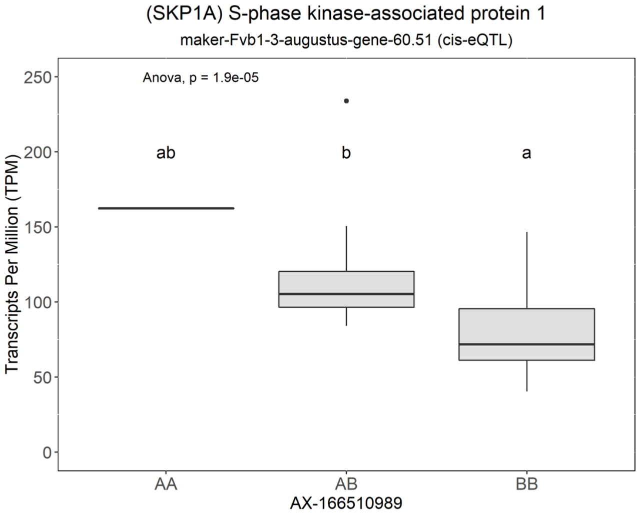
(SKP1A) S-phase kinase-associated protein 1
maker-Fvb1-3-augustus-gene-60.51 (cis-eQTL)
Anova, p = 1.9e-05
ab
b
a
Transcripts Per Million (TPM)
250
200
150
100
50
0
AA
AB
BB
AX-166510989

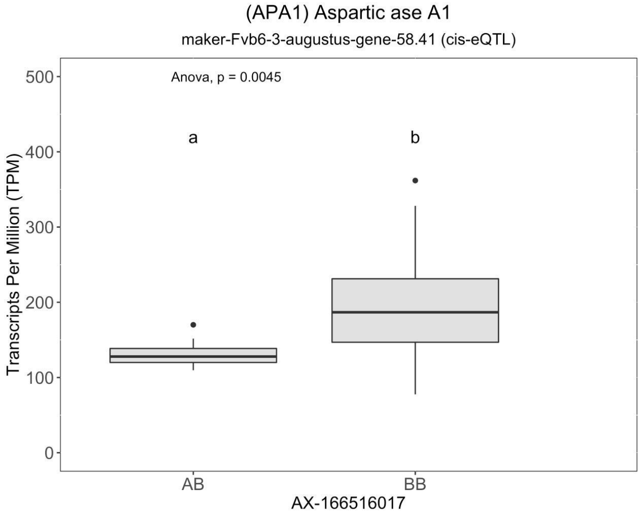
(APA1) Aspartic ase A1
maker-Fvb6-3-augustus-gene-58.41 (cis-eQTL)
Anova, p = 0.0045
a
b
Transcripts Per Million (TPM)
500
400
300
200
100
0
AB
BB
AX-166516017

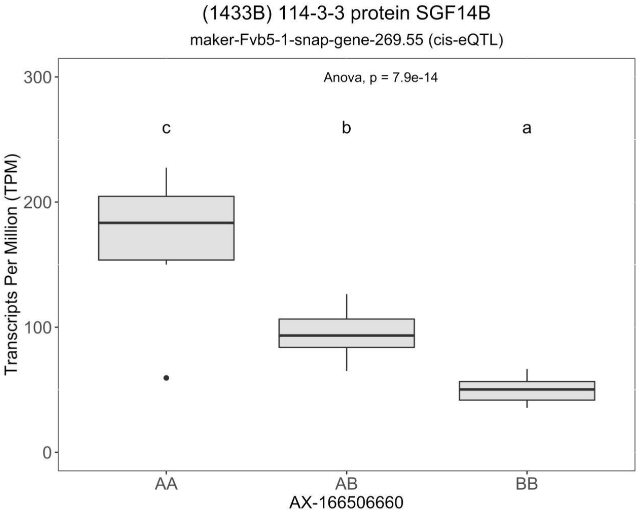
(1433B) 114-3-3 protein SGF14B
maker-Fvb5-1-snap-gene-269.55 (cis-eQTL)
Anova, p = 7.9e-14
c
b
a
Transcripts Per Million (TPM)
300
200
100
0
AA
AB
BB
AX-166506660

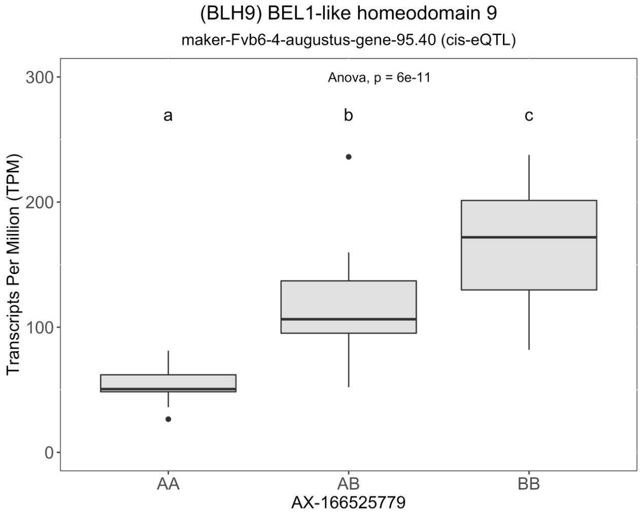
(BLH9) BEL1-like homeodomain 9
maker-Fvb6-4-augustus-gene-95.40 (cis-eQTL)
Anova, p = 6e-11
a
b
c
Transcripts Per Million (TPM)
300
200
100
0
AA
AB
BB
AX-166525779

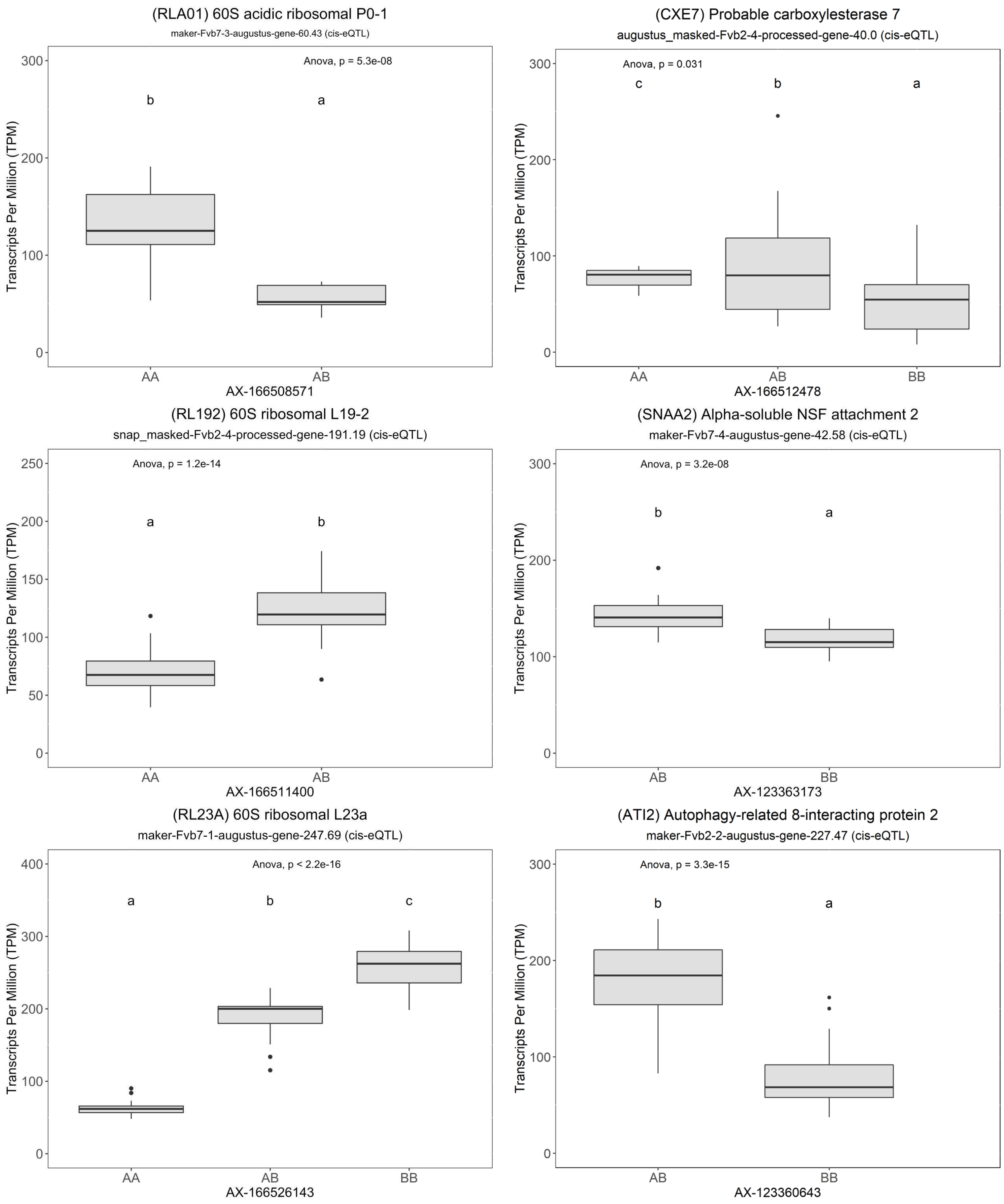
(RLA01) 60S acidic ribosomal P0-1
maker-Fvb7-3-augustus-gene-60.43 (cis-eQTL)
Anova, p = 5.3e-08
b
a
Transcripts Per Million (TPM)
0
100
200
300
AA
AB
AX-166508571
(CXE7) Probable carboxylesterase 7
augustus_masked-Fvb2-4-processed-gene-40.0 (cis-eQTL)
Anova, p = 0.031
c
b
a
Transcripts Per Million (TPM)
0
100
200
300
AA
AB
BB
AX-166512478
(RL192) 60S ribosomal L19-2
snap_masked-Fvb2-4-processed-gene-191.19 (cis-eQTL)
Anova, p = 1.2e-14
a
b
Transcripts Per Million (TPM)
0
50
100
150
200
250
AA
AB
AX-166511400
(SNAA2) Alpha-soluble NSF attachment 2
maker-Fvb7-4-augustus-gene-42.58 (cis-eQTL)
Anova, p = 3.2e-08
b
a
Transcripts Per Million (TPM)
0
100
200
300
AB
BB
AX-123363173
(RL23A) 60S ribosomal L23a
maker-Fvb7-1-augustus-gene-247.69 (cis-eQTL)
Anova, p < 2.2e-16
a
b
c
Transcripts Per Million (TPM)
0
100
200
300
400
AA
AB
BB
AX-166526143
(ATI2) Autophagy-related 8-interacting protein 2
maker-Fvb2-2-augustus-gene-227.47 (cis-eQTL)
Anova, p = 3.3e-15
b
a
Transcripts Per Million (TPM)
0
100
200
300
AB
BB
AX-123360643

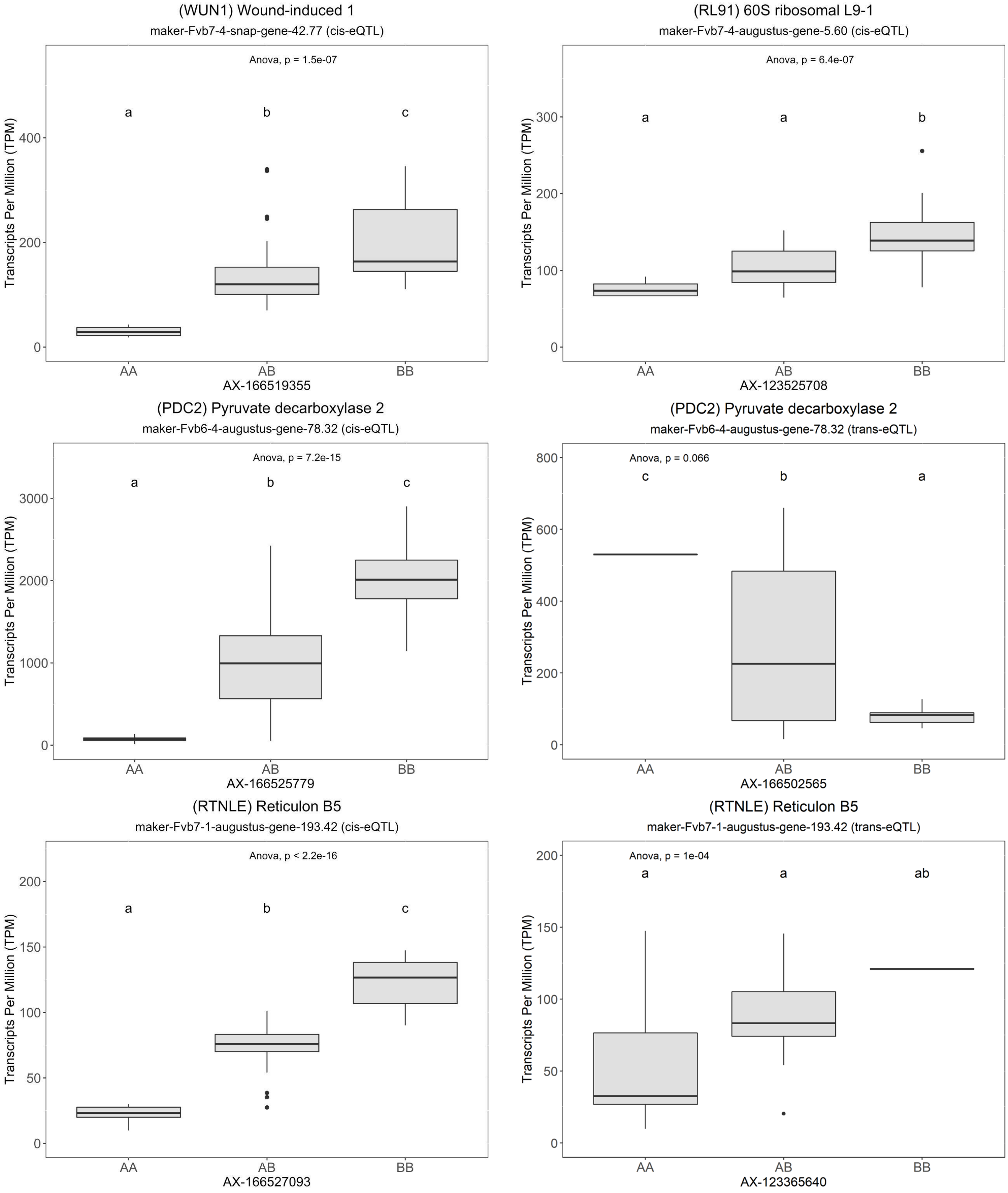
(WUN1) Wound-induced 1
maker-Fvb7-4-snap-gene-42.77 (cis-eQTL)
Anova, p = 1.5e-07
a
b
c
Transcripts Per Million (TPM)
0
200
400
AA
AB
BB
AX-166519355
(RL91) 60S ribosomal L9-1
maker-Fvb7-4-augustus-gene-5.60 (cis-eQTL)
Anova, p = 6.4e-07
a
a
b
Transcripts Per Million (TPM)
0
100
200
300
AA
AB
BB
AX-123525708
(PDC2) Pyruvate decarboxylase 2
maker-Fvb6-4-augustus-gene-78.32 (cis-eQTL)
Anova, p = 7.2e-15
a
b
c
Transcripts Per Million (TPM)
0
1000
2000
3000
AA
AB
BB
AX-166525779
(PDC2) Pyruvate decarboxylase 2
maker-Fvb6-4-augustus-gene-78.32 (trans-eQTL)
Anova, p = 0.066
c
b
a
Transcripts Per Million (TPM)
0
200
400
600
800
AA
AB
BB
AX-166502565
(RTNLE) Reticulon B5
maker-Fvb7-1-augustus-gene-193.42 (cis-eQTL)
Anova, p < 2.2e-16
a
b
c
Transcripts Per Million (TPM)
0
50
100
150
200
AA
AB
BB
AX-166527093
(RTNLE) Reticulon B5
maker-Fvb7-1-augustus-gene-193.42 (trans-eQTL)
Anova, p = 1e-04
a
a
ab
Transcripts Per Million (TPM)
0
50
100
150
200
AA
AB
BB
AX-123365640

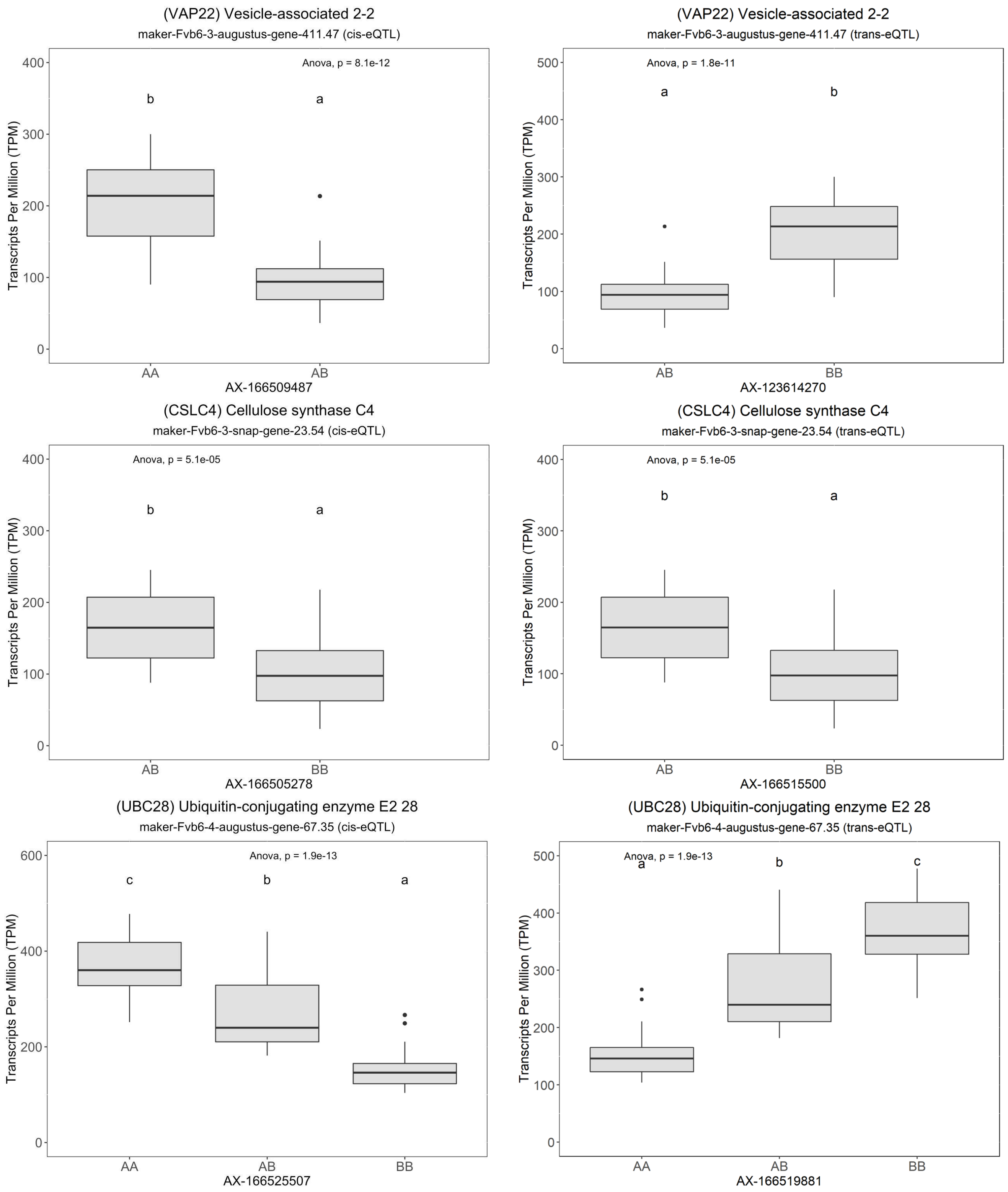
(VAP22) Vesicle-associated 2-2
maker-Fvb6-3-augustus-gene-411.47 (cis-eQTL)
Anova, p = 8.1e-12
Transcripts Per Million (TPM)
AX-166509487
(VAP22) Vesicle-associated 2-2
maker-Fvb6-3-augustus-gene-411.47 (trans-eQTL)
Anova, p = 1.8e-11
AX-123614270
(CSLC4) Cellulose synthase C4
maker-Fvb6-3-snap-gene-23.54 (cis-eQTL)
Anova, p = 5.1e-05
AX-166505278
(CSLC4) Cellulose synthase C4
maker-Fvb6-3-snap-gene-23.54 (trans-eQTL)
Anova, p = 5.1e-05
AX-166515500
(UBC28) Ubiquitin-conjugating enzyme E2 28
maker-Fvb6-4-augustus-gene-67.35 (cis-eQTL)
Anova, p = 1.9e-13
AX-166525507
(UBC28) Ubiquitin-conjugating enzyme E2 28
maker-Fvb6-4-augustus-gene-67.35 (trans-eQTL)
Anova, p = 1.9e-13
AX-166519881

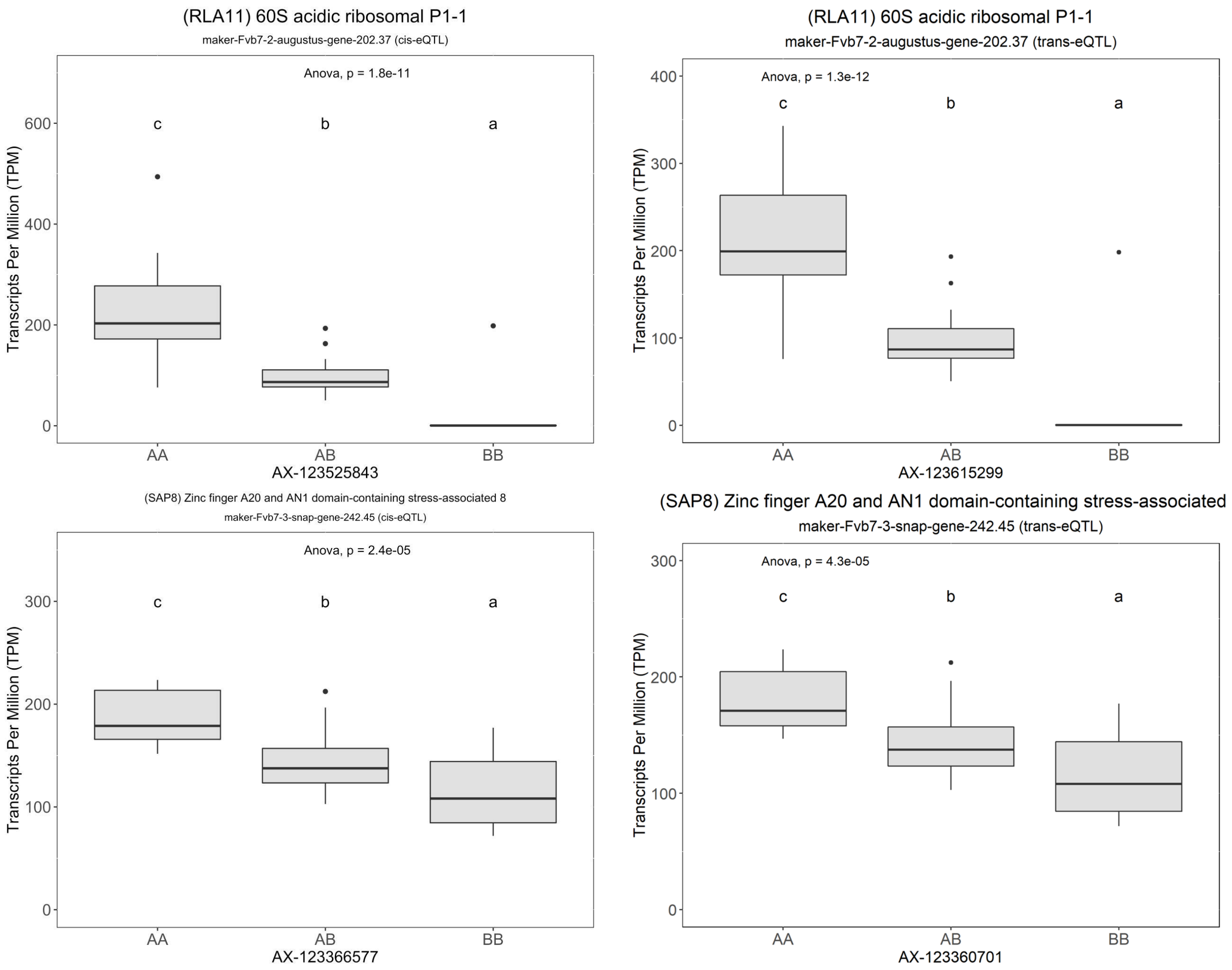

(RLA11) 60S acidic ribosomal P1-1
maker-Fvb7-2-augustus-gene-202.37 (cis-eQTL)
Anova, p = 1.8e-11
c
b
a
Transcripts Per Million (TPM)
0
200
400
600
AA
AB
BB
AX-123525843
(RLA11) 60S acidic ribosomal P1-1
maker-Fvb7-2-augustus-gene-202.37 (trans-eQTL)
Anova, p = 1.3e-12
c
b
a
Transcripts Per Million (TPM)
0
100
200
300
400
AA
AB
BB
AX-123615299
(SAP8) Zinc finger A20 and AN1 domain-containing stress-associated 8
maker-Fvb7-3-snap-gene-242.45 (cis-eQTL)
Anova, p = 2.4e-05
c
b
a
Transcripts Per Million (TPM)
0
100
200
300
AA
AB
BB
AX-123366577
(SAP8) Zinc finger A20 and AN1 domain-containing stress-associated
maker-Fvb7-3-snap-gene-242.45 (trans-eQTL)
Anova, p = 4.3e-05
c
b
a
Transcripts Per Million (TPM)
0
100
200
300
AA
AB
BB
AX-123360701
