# Supplementary material for: The Genetics of Differential Gene Expression Related to Fruit Traits in Strawberry (Fragaria ×ananassa)
Source: Front Genet. 2020 Feb 7;10:1317. doi: 10.3389/fgene.2019.01317 (PMC7025477; doi:10.3389/fgene.2019.01317)

File S3. Manhattan plots for select fruit-expressed genes associated with an eQTL.

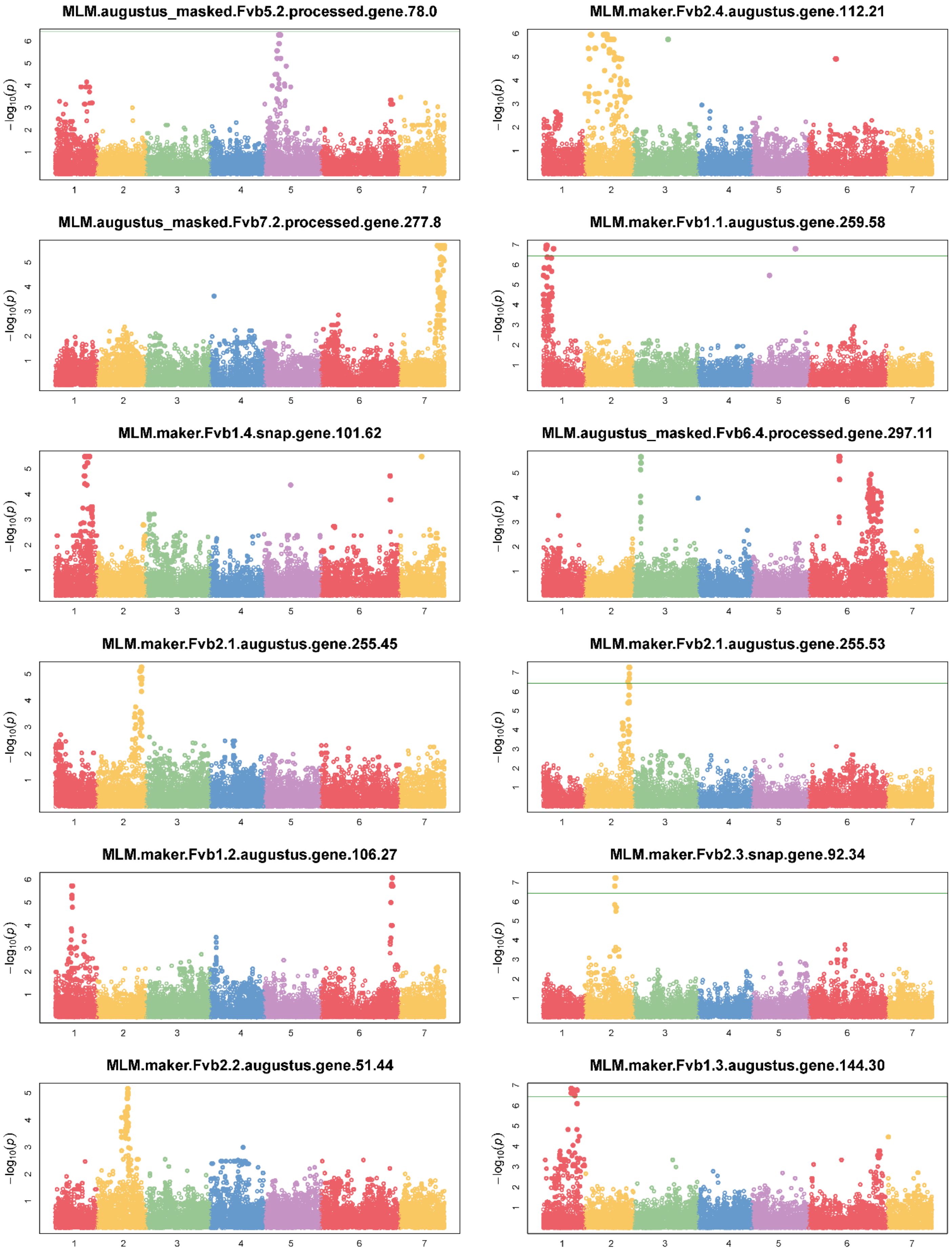

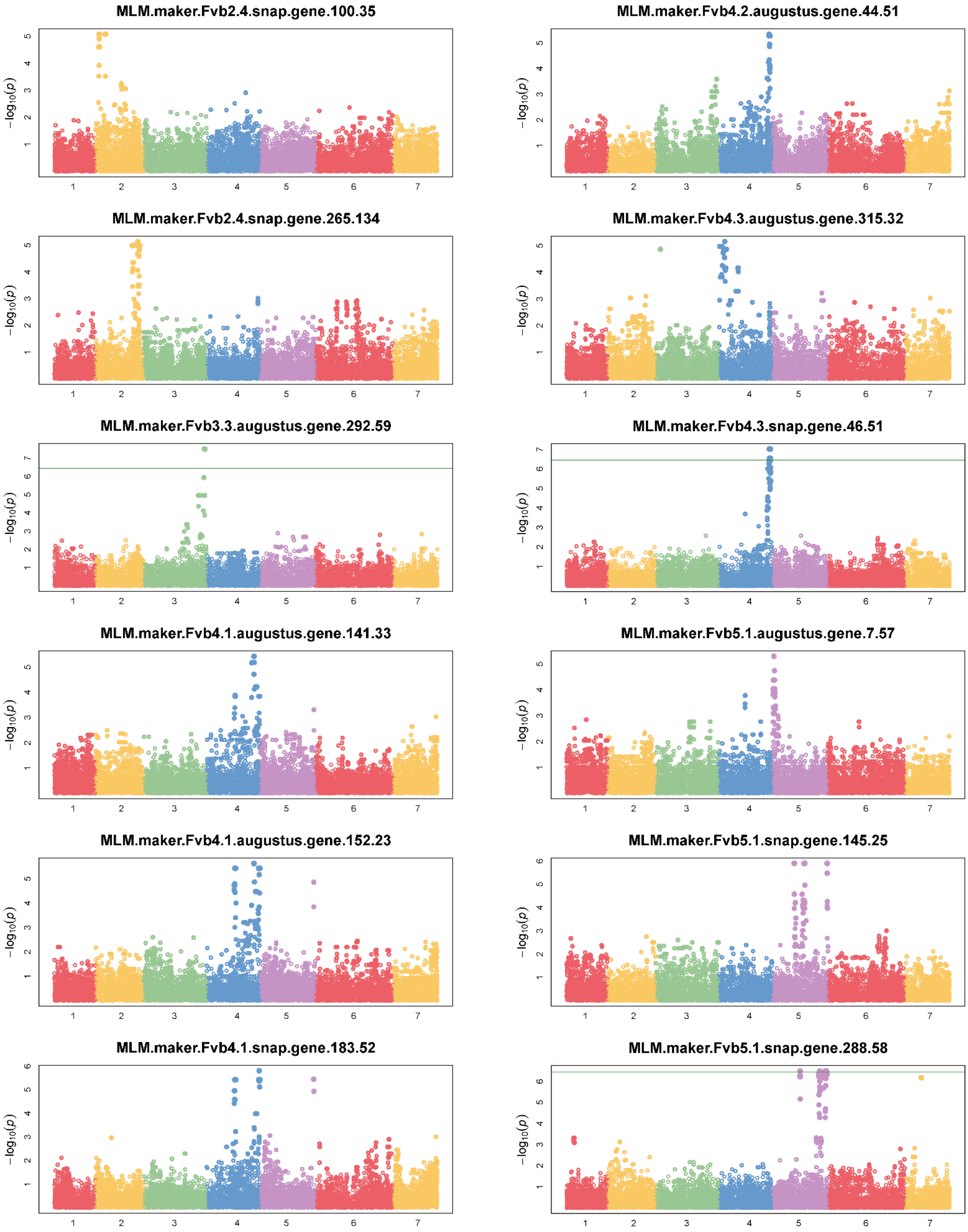
MLM.maker.Fvb2.4.snap.gene.100.35
MLM.maker.Fvb4.2.augustus.gene.44.51
MLM.maker.Fvb2.4.snap.gene.265.134
MLM.maker.Fvb4.3.augustus.gene.315.32
MLM.maker.Fvb3.3.augustus.gene.292.59
MLM.maker.Fvb4.3.snap.gene.46.51
MLM.maker.Fvb4.1.augustus.gene.141.33
MLM.maker.Fvb5.1.augustus.gene.7.57
MLM.maker.Fvb4.1.augustus.gene.152.23
MLM.maker.Fvb5.1.snap.gene.145.25
MLM.maker.Fvb4.1.snap.gene.183.52
MLM.maker.Fvb5.1.snap.gene.288.58
$-\log_{10}(p)$
1
2
3
4
5
6
7

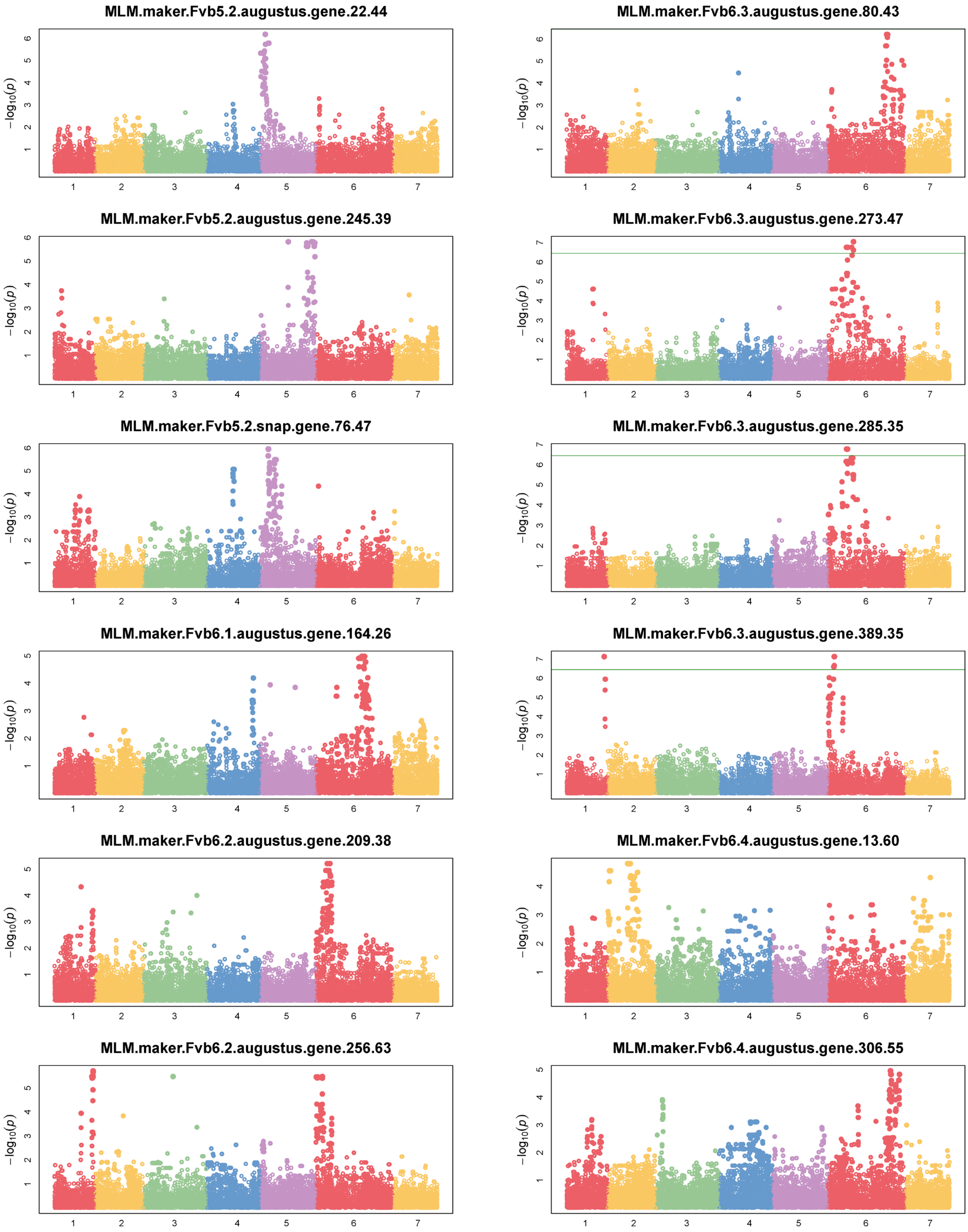
MLM.maker.Fvb5.2.augustus.gene.22.44
MLM.maker.Fvb6.3.augustus.gene.80.43
MLM.maker.Fvb5.2.augustus.gene.245.39
MLM.maker.Fvb6.3.augustus.gene.273.47
MLM.maker.Fvb5.2.snap.gene.76.47
MLM.maker.Fvb6.3.augustus.gene.285.35
MLM.maker.Fvb6.1.augustus.gene.164.26
MLM.maker.Fvb6.3.augustus.gene.389.35
MLM.maker.Fvb6.2.augustus.gene.209.38
MLM.maker.Fvb6.4.augustus.gene.13.60
MLM.maker.Fvb6.2.augustus.gene.256.63
MLM.maker.Fvb6.4.augustus.gene.306.55
$-\log_{10}(p)$
1 2 3 4 5 6 7

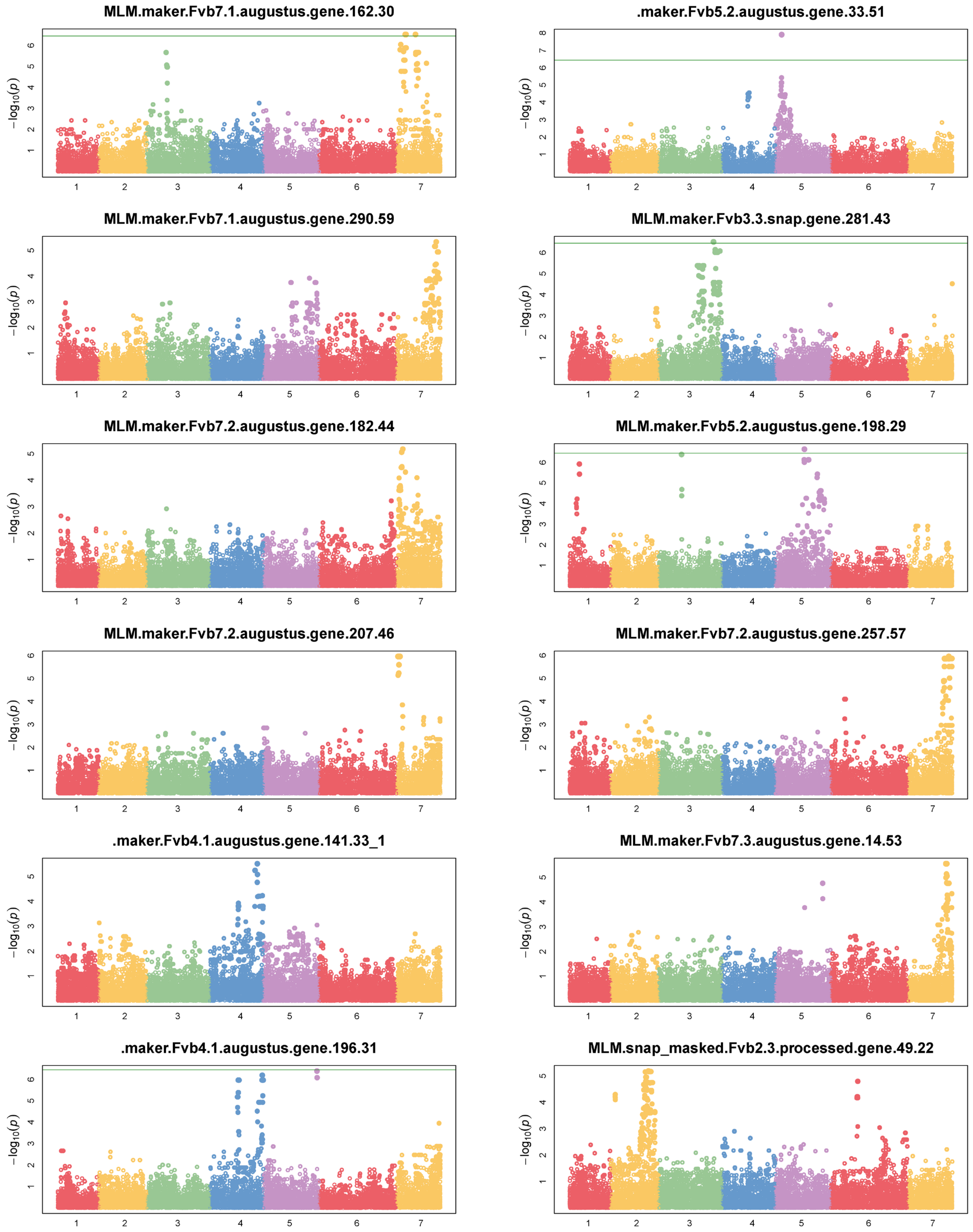
MLM.maker.Fvb7.1.augustus.gene.162.30
.maker.Fvb5.2.augustus.gene.33.51
MLM.maker.Fvb7.1.augustus.gene.290.59
MLM.maker.Fvb3.3.snap.gene.281.43
MLM.maker.Fvb7.2.augustus.gene.182.44
MLM.maker.Fvb5.2.augustus.gene.198.29
MLM.maker.Fvb7.2.augustus.gene.207.46
MLM.maker.Fvb7.2.augustus.gene.257.57
.maker.Fvb4.1.augustus.gene.141.33_1
MLM.maker.Fvb7.3.augustus.gene.14.53
.maker.Fvb4.1.augustus.gene.196.31
MLM.snap_masked.Fvb2.3.processed.gene.49.22
$-\log_{10}(p)$
1 2 3 4 5 6 7

MLM.snap_masked.Fvb6.4.processed.gene.308.25

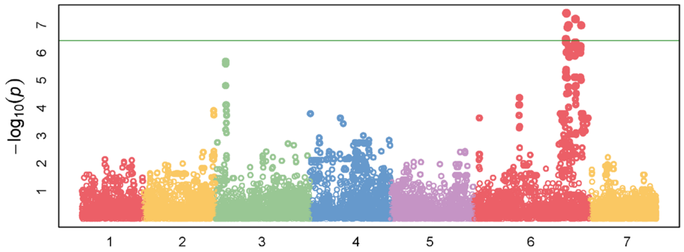

MLM.snap_masked.Fvb7.4.processed.gene.40.42

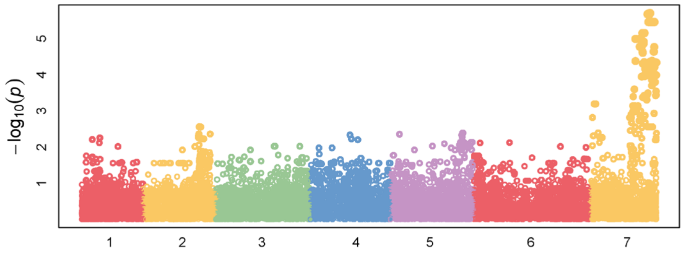

Supplement: Supplementary file 3 [file DataSheet_3.pdf]
