# Supplementary material for: The Genetics of Differential Gene Expression Related to Fruit Traits in Strawberry (Fragaria ×ananassa)
Source: Front Genet. 2020 Feb 7;10:1317. doi: 10.3389/fgene.2019.01317 (PMC7025477; doi:10.3389/fgene.2019.01317)
Supplement: Supplementary file 5 [file Presentation_1.pdf]

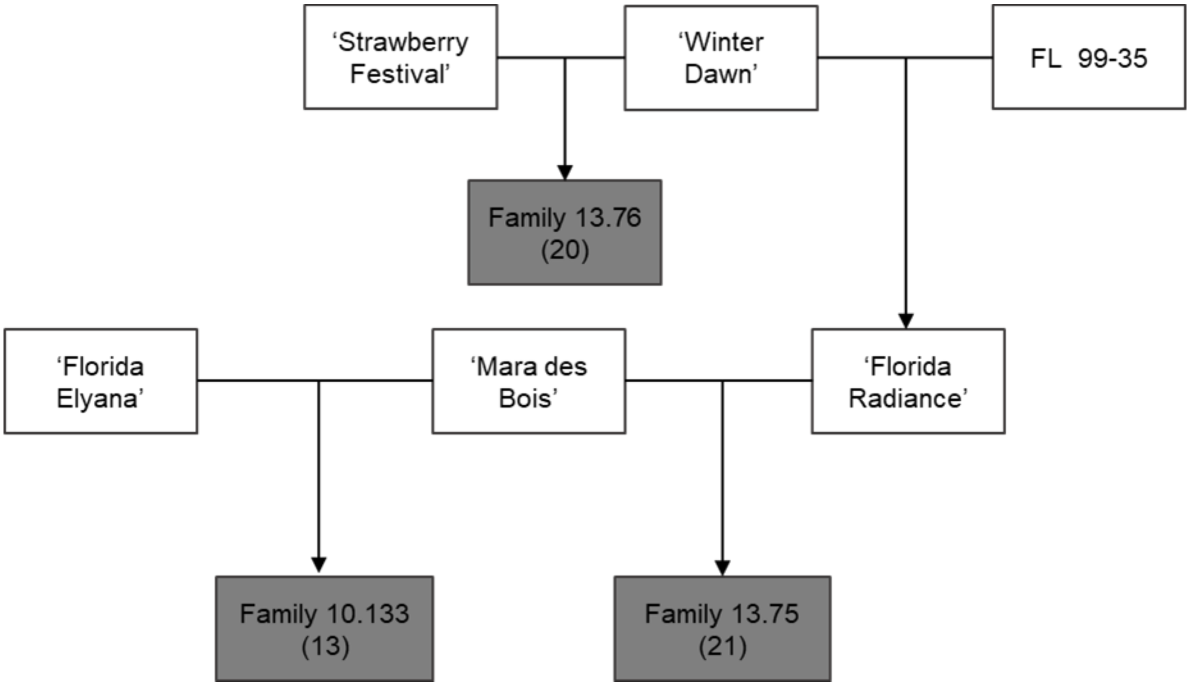

Figure S1. Pedigrees of three strawberry families used in RNAseq and eQTL analysis (dark gray). The number of progeny used in the analysis is indicated in parenthesis. Parents were also utilized for this analysis.

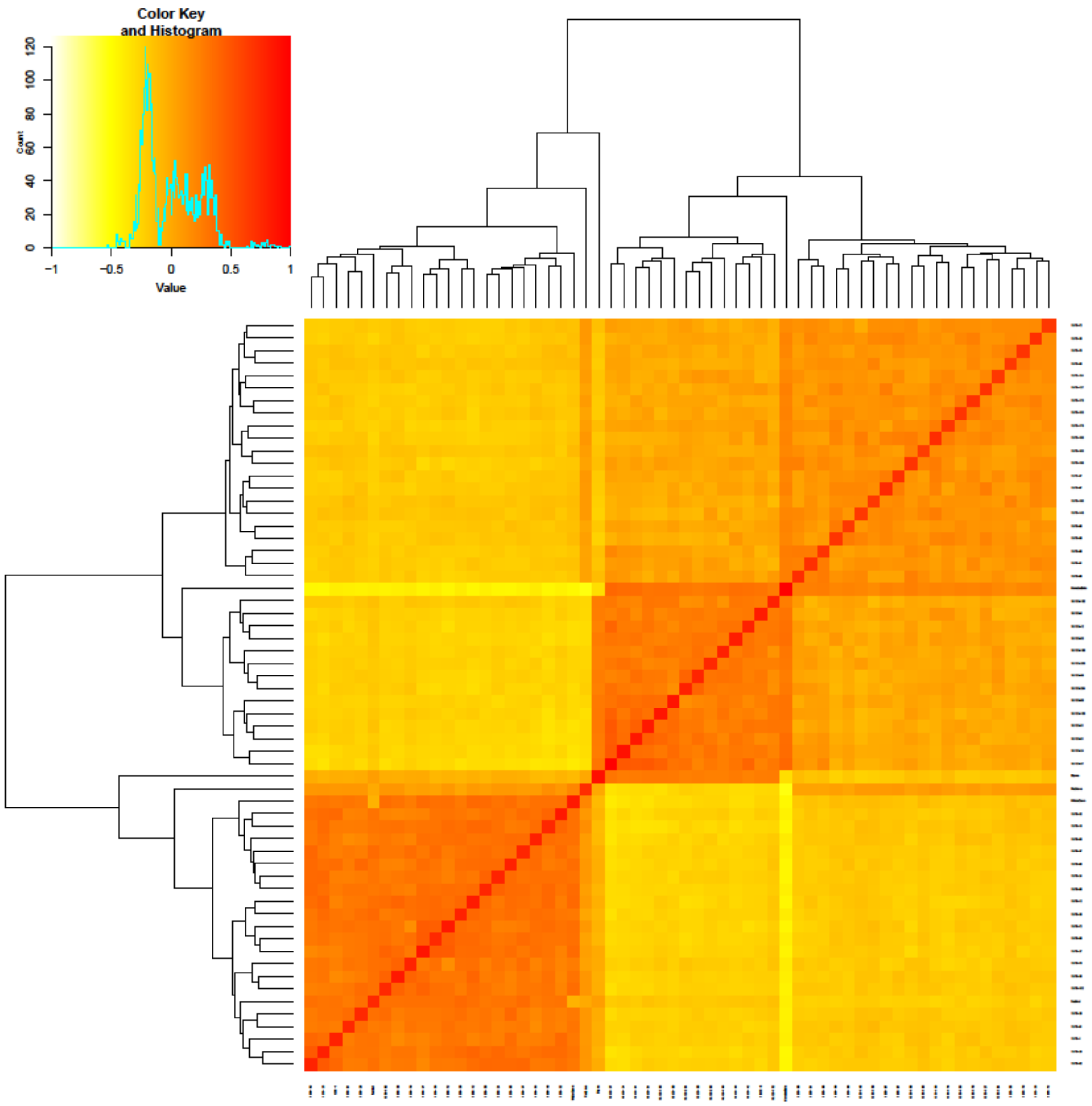

Figure S2. Kinship relatedness in the eQTL study populations. Genotype comparisons via VanRaden kinship method are shown from parents and progeny from ‘Florida Elyana’ × ‘Mara des Bois’, ‘Florida Radiance’ × ‘Mara des Bois’, and ‘Strawberry Festival’ × ‘Winter Dawn’.

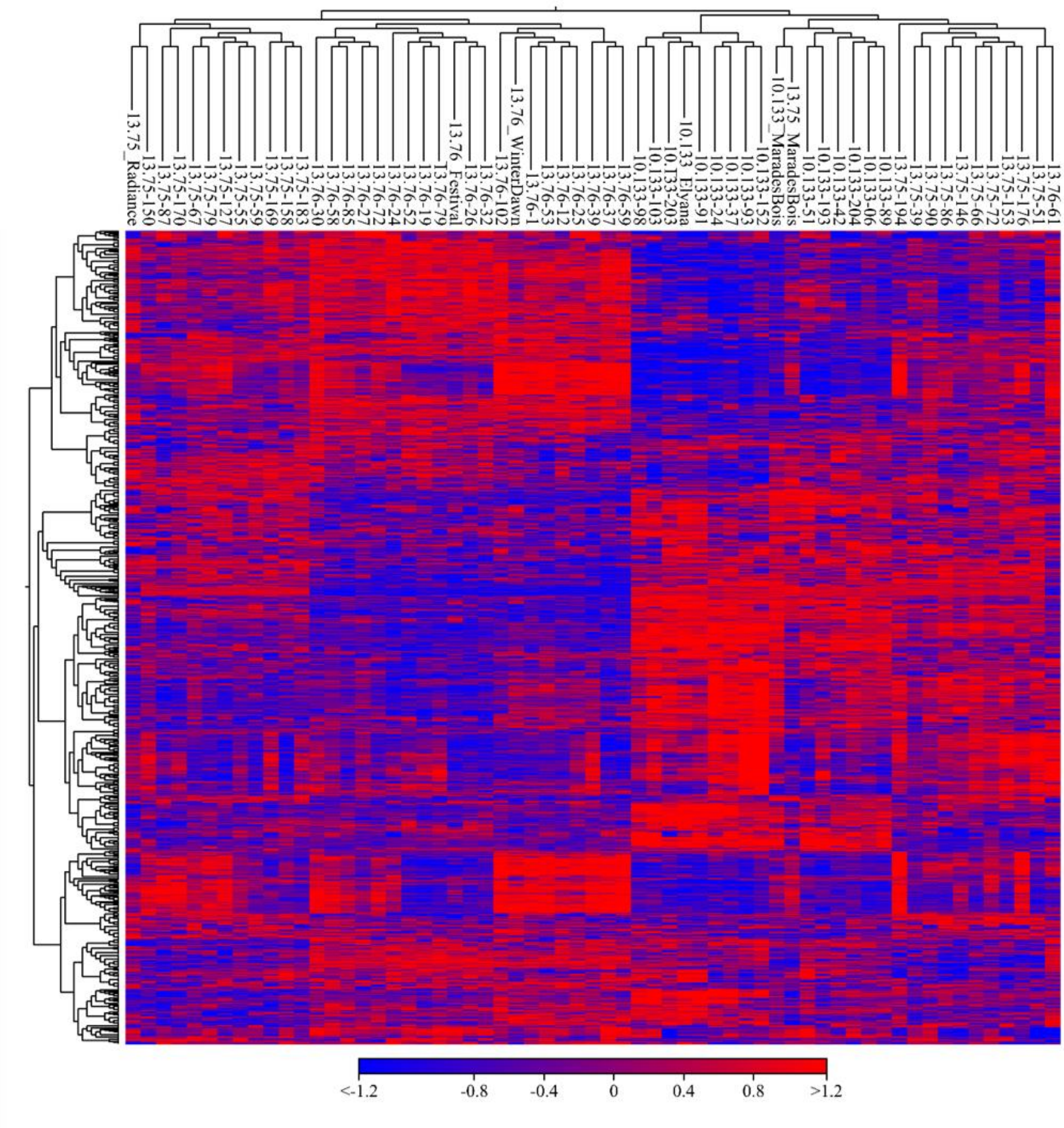

Figure S3. High-variance octoploid transcripts for eQTL analysis. Heatmap analysis of 2,000 octoploid *Fragaria ×ananassa* fruit transcripts (left dendrogram), with the highest coefficient of variance among 61 genotypes (top dendrogram). RNAseq reads were mapped to the octoploid 'Camarosa' genome and represent homoeolog-specific transcriptome assemblies. Blue-to-red legend indicates low-to-high expression, respectively, with values indicated in normalized log counts per million.

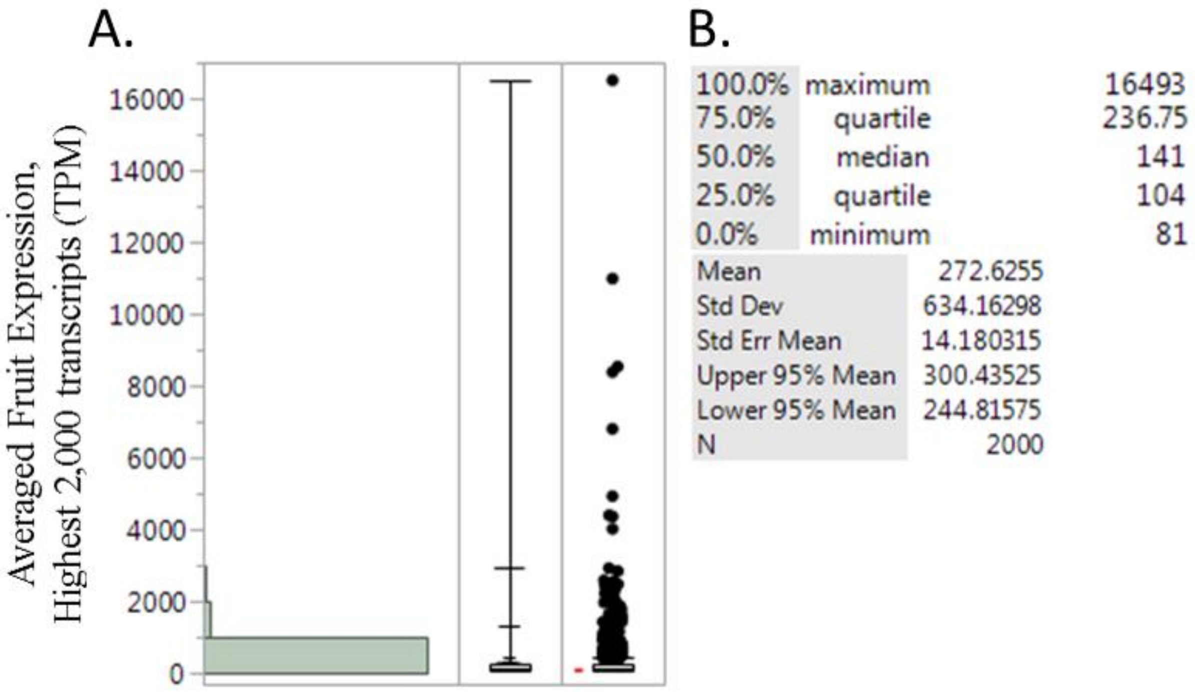

Figure S4. High-expression octoploid transcripts for eQTL analysis. The 2,000 octoploid *Fragaria ×ananassa* transcripts with the highest average expression among 61 genotypes are compared. A. Expression comparisons of highly expressed genes via barplot, boxplot and scatterplot analysis. B. Corresponding quartiles, means, errors, and minimum/maximums.
